# Comparative genomics of small RNA regulatory pathway components in vector mosquitoes

**DOI:** 10.1186/1471-2164-9-425

**Published:** 2008-09-18

**Authors:** Corey L Campbell, William C Black, Ann M Hess, Brian D Foy

**Affiliations:** 1 Arthropod-borne Infectious Diseases Laboratory; Microbiology, Immunology, and Pathology Department, Colorado State University, Fort Collins, Colorado, 80523, USA; 2 Department of Statistics and Center for Bioinformatics, Colorado State University, Fort Collins, Colorado, 80523, USA

## Abstract

**Background:**

Small RNA regulatory pathways (SRRPs) control key aspects of development and anti-viral defense in metazoans. Members of the Argonaute family of catalytic enzymes degrade target RNAs in each of these pathways. SRRPs include the microRNA, small interfering RNA (siRNA) and PIWI-type gene silencing pathways. Mosquitoes generate viral siRNAs when infected with RNA arboviruses. However, in some mosquitoes, arboviruses survive antiviral RNA interference (RNAi) and are transmitted via mosquito bite to a subsequent host. Increased knowledge of these pathways and functional components should increase understanding of the limitations of anti-viral defense in vector mosquitoes. To do this, we compared the genomic structure of SRRP components across three mosquito species and three major small RNA pathways.

**Results:**

The *Ae. aegypti, An. gambiae *and *Cx. pipiens *genomes encode putative orthologs for all major components of the miRNA, siRNA, and piRNA pathways. *Ae. aegypti *and *Cx. pipiens *have undergone expansion of Argonaute and PIWI subfamily genes. Phylogenetic analyses were performed for these protein families. In addition, sequence pattern recognition algorithms MEME, MDScan and Weeder were used to identify upstream regulatory motifs for all SRRP components. Statistical analyses confirmed enrichment of species-specific and pathway-specific cis-elements over the rest of the genome.

**Conclusion:**

Analysis of Argonaute and PIWI subfamily genes suggests that the small regulatory RNA pathways of the major arbovirus vectors, *Ae. aegypti and Cx. pipiens*, are evolving faster than those of the malaria vector *An. gambiae *and *D. melanogaster*. Further, protein and genomic features suggest functional differences between subclasses of PIWI proteins and provide a basis for future analyses. Common UCR elements among SRRP components indicate that 1) key components from the miRNA, siRNA, and piRNA pathways contain NF-kappaB-related and Broad complex transcription factor binding sites, 2) purifying selection has occurred to maintain common pathway-specific elements across mosquito species and 3) species-specific differences in upstream elements suggest that there may be differences in regulatory control among mosquito species. Implications for arbovirus vector competence in mosquitoes are discussed.

## Background

Small RNA-mediated gene silencing pathways are master regulators of critical cellular processes, from development to germ-line surveillance to anti-viral defense [1-5]. Although small RNA regulatory pathways (SRRPs) operate using distinctly different mechanisms, they share several common features. Small regulatory RNAs of 20 to 30 nucleotides (nts) are used as guide strands for target substrate recognition by an RNase H type nuclease of the Argonaute protein family. Target RNAs are subsequently degraded or otherwise prevented from being translated into protein. The three major pathways are the small interfering RNA (siRNA), microRNA (miRNA) and PIWI small RNA (piRNA) pathways; the general functions of each are summarized in Table 1. Due to the paucity of functional information from mosquitoes, we have relied on protein structure and functional information compiled from *Drosophila *spp., *Caenorhabditis elegans*, and mammals.

**Table 1:** Argonaute protein family functional groups

**Protein Subfamily**	**Protein**	**Small RNA class, length**	**Putative Function **(referenced mosquito studies)
Argonaute	Ago1	miRNA, 20–22 nts	Regulation of endogenous mRNAs during development; control of Plasmodium infection [15,54]
Argonaute	Ago2	siRNA, 20–22 nts	Anti-viral defense [1,25-27]
PIWI	Ago3 PIWI (Ago4) Aub (Ago5)	piRNA, rasiRNA, 24–30 nts	Germline surveillance, telomere maintenance, anti-viral defense [1], and maintenance of retrotransposon silencing

The Argonaute protein family contains the Argonaute and PIWI protein subclasses. All proteins in this family contain PAZ (PIWI, Argonaute, Zwille) and PIWI domains [6]. The PAZ domain is a small RNA binding domain; the PIWI domain is an RNase-H type domain which relies on divalent cation binding to facilitate dsRNA-guided cleavage of ssRNA (reviewed in [7]). Argonaute 1 (Ago1) and Argonaute 2 (Ago2) are in the Argonaute protein subclass and are functionally distinct from PIWIs, in that they rely on Dicer proteins to cleave long dsRNAs into small RNAs, which are then passed to the Agos for further processing and recognition of target RNAs. These proteins physically interact with Dicers at the PIWI domain and use small RNAs formed from double-stranded templates [8]. Ago1 and Ago2 each participate in different gene silencing pathways.

**Figure 1:**
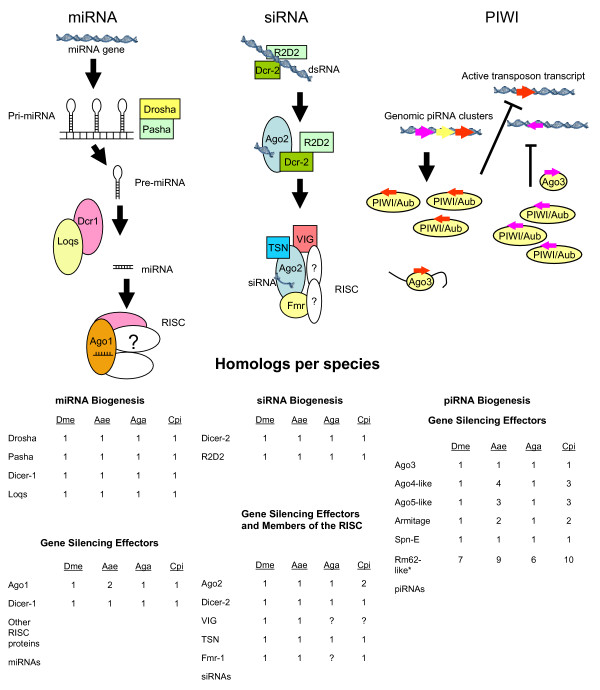
**Small RNA Regulatory Pathways**. Schematic and functional categories as known from mechanistic studies in model organisms. The number of homologs for each mosquito species is compared to those of *D. melanogaster*. "*", the Rm62-like RNA helicase family is large and complex in diptera; only DmeRm62, NM_169118, has been associated with RNA interference. The PIWI pathway model was adapted from previously published diagrams [35,94].

MiRNA pathways of invertebrates have been best characterized in *D. melanogaster *and *C. elegans *(reviewed in [9,10]). In *D. melanogaster*, developmental and housekeeping gene expression is regulated by Ago1 and the small RNA subclass, miRNAs (20–22 nts) [11]. In general, Ago1 acts with Dicer-1, microRNAs (miRNAs) and accessory proteins to control gene expression of housekeeping genes (Figure 1) [12-14]. In *An. gambiae*, the miRNA pathway helps defend against *Plasmodium bergei *infection [15]. Gene expression can be controlled by a variety of possible silencing mechanisms, including translational repression, decreased mRNA stability or altered mRNA localization [3,16,17]. These small RNAs are genome-encoded as primary transcript miRNAs (pri-miRNAs) up to 2 kilobases (kB) in length and processed into miRNA precursor (pre-miRNAs) hairpin loops of about 65 nts by Drosha and Pasha (DGCR8) [18,19]. Dicer-1 cleaves pre-miRNAs to form mature miRNAs, which are transferred to Ago1 for silencing of target mRNAs [3,20,21]. In mammals, some miRNAs are encoded in the introns of the genes they target [22].

The siRNA pathway provides defense against RNA viruses in the degradation of double-stranded viral RNAs [1,8,23,24]. SiRNAs, generated from longer dsRNA molecules by the Dicer-2/R2D2 complex, serve as guides for Ago2 to identify and degrade target RNAs. Key proteins in the anti-viral RNA interference (RNAi) pathway, including Ago2 and Dicer-2, are functional in vector mosquitoes and influence the ability of mosquitoes to serve as competent vectors of human virus pathogens [1,25-27]. Further, several recent reports of *Drosophila *have shown that endogenous siRNAs control retrotransposons and mRNAs in somatic cells in an Ago2/Dicer-2 dependent manner [28-30].

In *Drosophila melanogaster*, the PIWI protein subclass includes PIWI, Aubergine (Aub), and Argonaute 3 (Ago3). These proteins contain the signature PAZ and PIWI domains, but have remained enigmatic members of the Argonaute family. They have been implicated in a variety of functions specific to germ-line tissue in *D. melanogaster*, including the suppression of retrotransposon mobilization, maintenance of telomeres, prevention of double-stranded DNA breaks during meiosis, and mRNA silencing in germ-line cells [4,5,31-34]. Ago3, Aub, and PIWI use PIWI-associated RNAs (piRNAs) (24 to 30 nts) in these pathways. The mechanisms for production of this class of non-coding RNAs are not well understood. Models have been proposed, suggesting they are processed from piRNA cluster transcripts and amplified by a "Ping Pong" amplification loop between these transcripts and those derived from active transposons (Figure 1) [35].

Three mosquito species, *Anopheles gambiae*, *Aedes aegypti*, and *Culex pipiens quinquefasciatus *are important vectors of human and animal pathogens [36-41]. The goal of this study is to increase understanding of the three small RNA pathways in vector mosquitoes using comparative genomics, with special emphasis on the key catalytic enzymes of the Argonaute protein family. We performed phylogenetic analyses of Argonaute family proteins and demonstrated expansion of *Ae. aegypti *and *Cx. pipiens *Argonaute and PIWI subfamilies. In addition, we determined whether *cis*-acting regulatory elements upstream of SRRP genes are conserved across mosquito species or among components of specific RNA regulatory pathways, as has been successfully demonstrated in other invertebrates [42]. To this end, previously validated mosquito transcription factor binding sites (TFBSs) and other elements in upstream control regions (UCRs) of all suspected SRRP components were identified. Further, we used motif discovery algorithms to identify novel upstream regulatory sequences. The identification of common *cis*-acting regulatory features lends insight into species-specific differences in regulation of SRRPs and provides a basis for future studies of pathogen defense in vector mosquitoes.

## Results and discussion

**Figure 2:**
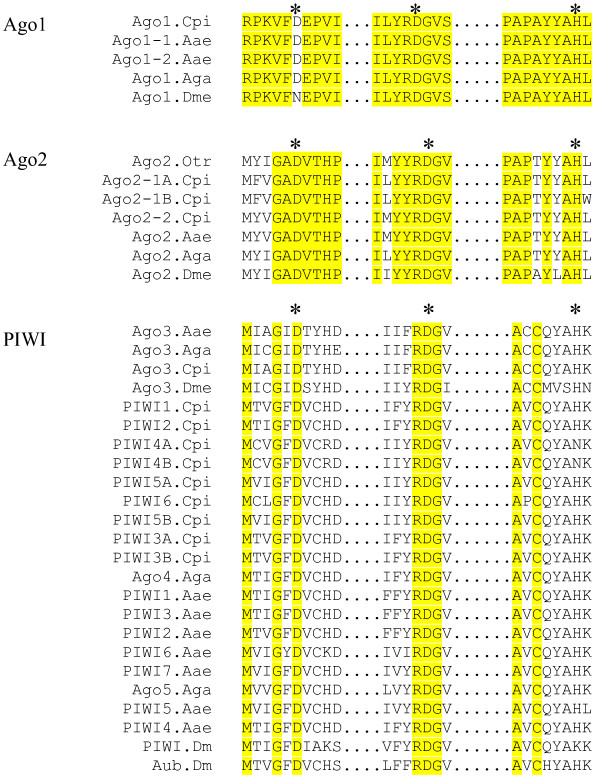
**Conservation of Argonaute Family Protein Key Catalytic Residues**. Mosquito PIWI and Argonaute orthologs retain key catalytic residues [7]. Stars indicate key residues. Yellow highlight indicates identical amino acids among species. Gene accession numbers: *Oc. triseriatus*, [Genbank: EU182829] or in Additional File 1A.

The *An. gambiae*, *Ae. aegypti*, and *Cx. pipiens *genomes encode components of the three major RNA regulatory pathways previously identified in *D. melanogaster *(Figure 1 and Additional File 1A) [1,8,11,12,24,26,43-53]. There are gene expansions in some categories; these are described below. Additional File 1A depicts, in gray highlight, the genes for which expression has been confirmed, either experimentally or in EST libraries [1,26]. Importantly, key catalytic residues of the RNase-H domains are conserved in all mosquito Ago1s, Ago2s and PIWI subfamily proteins (Figure 2) [7]. Although a few studies have been done in *Ae. aegypti *and anopheline mosquitoes [1,15,25,54], experimental verification will be required to confirm the roles played by most of these putative orthologs in RNA regulatory pathways.

### Argonaute protein subfamily

*Ago1 *is represented at a single genomic locus in *An. gambiae*, *Cx. pipiens *and *D. melanogaster *but seems to be present in two copies in *Ae. aegypti *(Figure 1, Additional File 1A). The flanking sequences of about 1.2 kilobases (kB) upstream and the introns were found to be unique.

**Figure 3:**
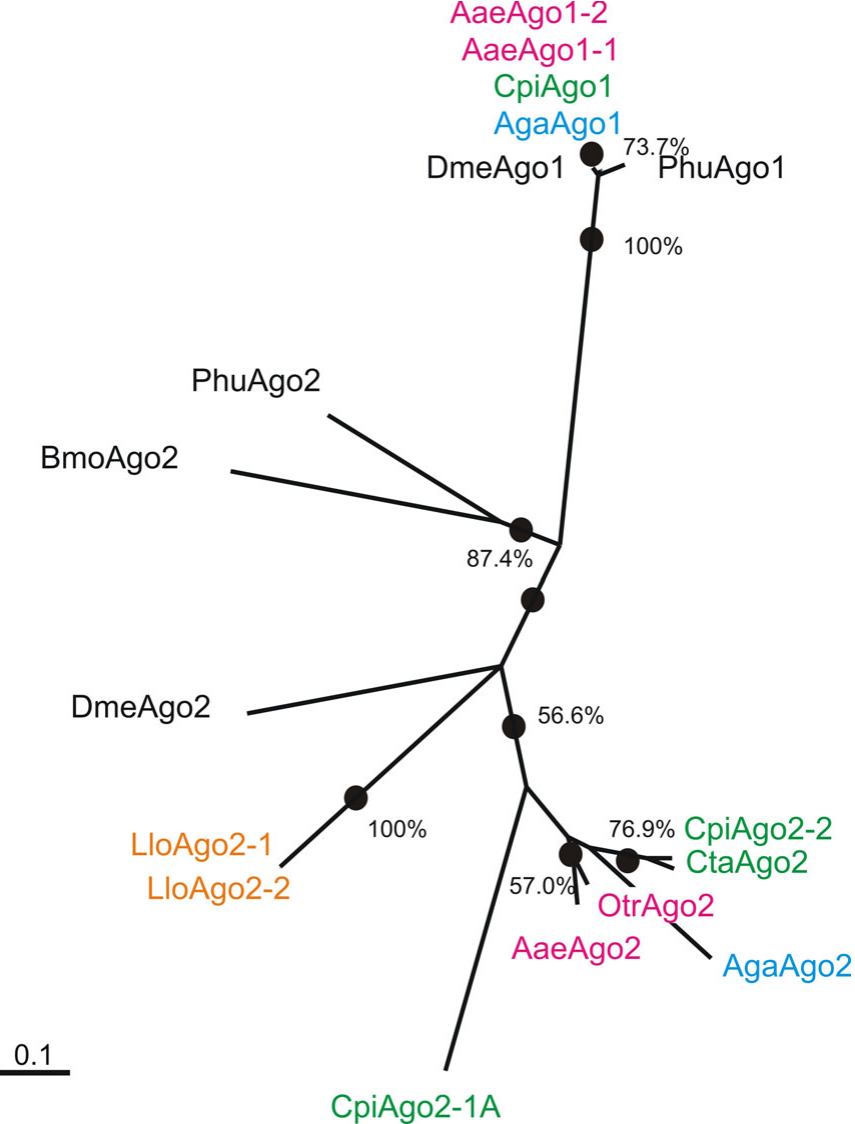
**Argonaute Subfamily Tree**. Argonaute protein subfamily maximum likelihood tree with bootstrap values. *Oc. triseriatus*, [Genbank: EU182829]; *Cx tarsalis*, [Genbank: EU182828]; *Lutzomyia longipalpis*, [Genbank: AM094709.1, AM094708.1]; Phum, *Pedicularis humanus*, [Vectorbase: phum002582]. All other accession numbers are listed in Additional File 1A. Bar equals 0.1000 amino acid substitution probability.

Ago1 of the human head louse, *Pediculus humanus *was used as an outgroup for the analysis of Argonaute subfamily proteins. Ago2 homologs from the silkworm, *Bombyx mori*, and *D. melanogaster *were also included in the analysis. In addition, orthologs from the important pathogen vectors, *Lutzomyia longipalpis, Culex tarsalis*, and *Ochlerotatus triseriatus *were included. Based upon the Jones-Taylor-Thornton probability model, the probability of amino acid substitutions among Ago1 proteins was 0.0247 (coefficient of variation (CV) = 100 * (standard deviation/mean) = 100.0%) while among Ago2 proteins the average probability was 0.9411 (CV = 30.1%) (Figure 3) [55]. Thus, the probability of amino acid substitutions among Ago2 proteins is about 38-fold greater than among Ago1 proteins. Because of this high rate of change among Ago2 proteins, there was only weak bootstrap support (56.6%) for the proteins from mosquitoes. However, among the two genera of the tribe Aedini it is interesting that between *Ochlerotatus triseriatus *and *Ae. aegypti *the substitution probability is only 0.1306, while within and among *Culex *species the probability is 0.3798 (CV = 68.9%). This pattern suggests that anti-viral pathway components are evolving more rapidly in *Culex spp. *than among subgenera of Aedine mosquitoes. An interesting parallel was found among drosophilid species, wherein key anti-viral RNAi component genes, such as *DmeAgo2 *and *DmeDicer-2*, were found to be evolving at a higher rate than miRNA pathway components or other immune response genes [56]. Synapomorphies that distinguish Ago1s from Ago2s can be found in the amino acids surrounding the first catalytic residue (Additional File 2).

Ago1 protein conservation indicates an evolutionary trend of purifying selection, probably due to conserved mechanisms in developmental pathways. This hypothesis is supported in the conservation of some miRNAs between drosophilids, mosquitoes, and humans [57]. Although many mosquito miRNAs have not been experimentally verified, their presence suggests conservation of developmentally regulated gene silencing pathways. Alignment of Ago1 proteins further illustrates the degree of conservation with overall amino acid identity of 82 to 89% among all mosquito orthologs and that of *D. melanogaster *(Additional File 1B). This is in contrast to the 30 to 43% amino acid identity among all mosquito Ago 2 proteins compared to DmeAgo2 (Additional File 1C).

*Ago2 *is a single locus in *Ae. aegypti*, *An. gambiae*, and *D. melanogaster*, but is present in two copies in *Cx. pipiens *(Figure 1). Fragments of two distinct mRNAs have also been isolated from *Cx. tritaeniorhyncus*, suggesting that a gene duplication occurred early in the evolution of *Culex spp. *(Campbell, unpublished). This first evidence of multiple *Ago2 *loci in Dipterans suggests that differential regulation of RNAi anti-viral defense could occur in *Culex spp. *vectors. Although anti-viral RNAi activity has not yet been reported for *Culex spp*., this pathway has been established as an important facet of anti-viral defense in *An. gambiae *and *Ae. aegypti *against alphaviruses and dengue viruses (DENV), respectively [1,25,26].

### PIWI pathway gene expansions

**Figure 4:**
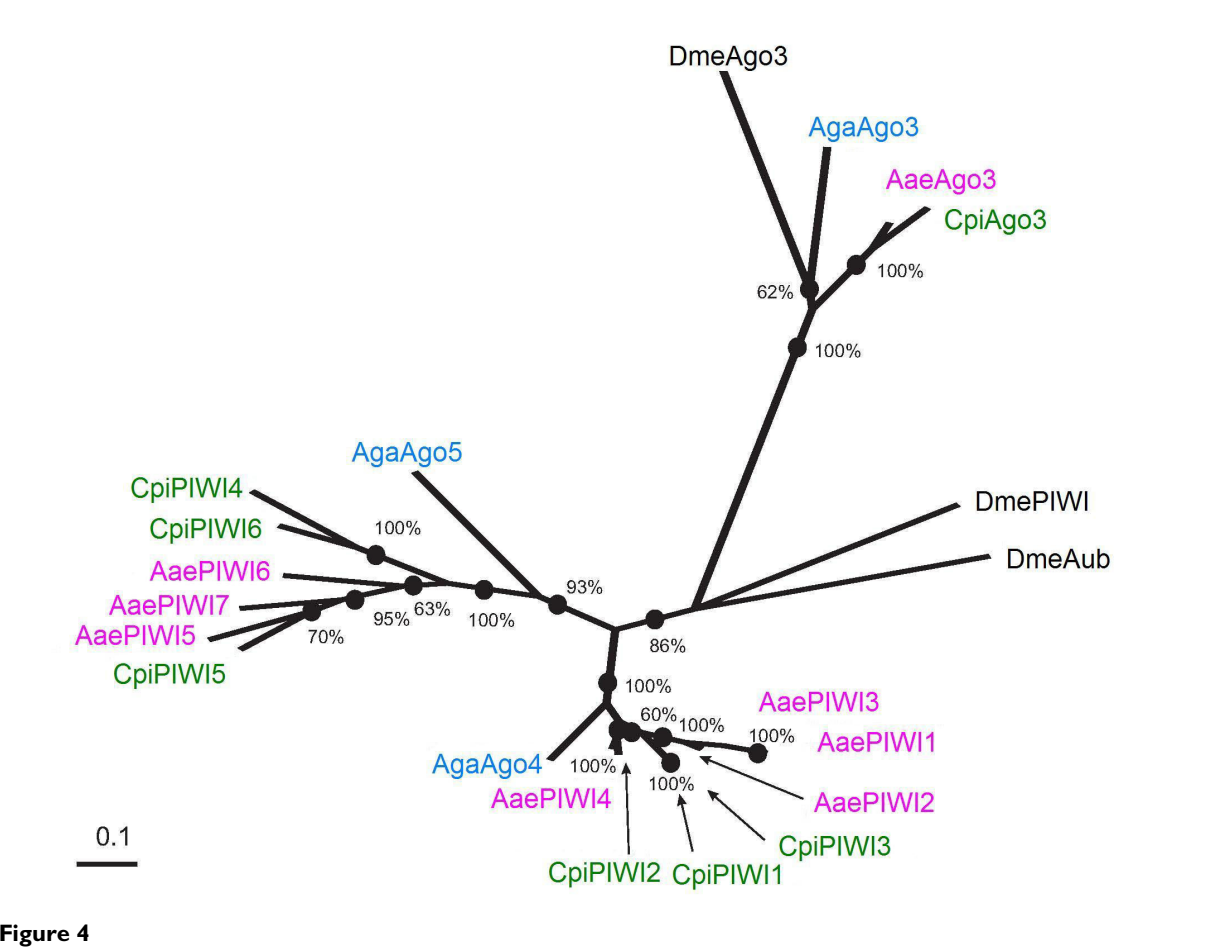
**PIWI Subfamily Tree**. PIWI protein subfamily maximum likelihood tree with bootstrap values. All accession numbers are listed in Additional File 1A. Bar equals 0.1000 amino acid substitution probability.

A significant gene expansion has occurred in PIWI subfamily proteins in both *Ae. aegypti *and *Cx. pipiens *(Figure 1) [48,51]. Phylogenetic analyses of PIWIs used *D. melanogaster *Aub and PIWI as outgroups (Figure 4). Three well-supported clades are evident. Ago3s form a single clade with *D. melanogaster*. In contrast, none of the mosquito PIWIs formed a clade with the drosophilid proteins, rather, two clades arise based on similarity to *An. gambiae *Ago4 or Ago5.

The first clade has 100% support and contains the Ago3 proteins. Ago3s can be readily distinguished from other Argonaute family proteins by the amino acids surrounding the first conserved catalytic residue (Figure 2 and Additional File 1D). The average rate of change among these is high 0.6872 (CV = 40.7%). The Ago3 proteins among the Culicidae are monophyletic with 100.0% support, and the average rate of change among these is also high 0.4615 (CV = 44.6%).

The Ago4-like clade has 100.0% bootstrap support and contains AgaAgo4, CpiPIWI1, CpiPIWI2, CpiPIWI3, AaePIWI1, AaePIWI2, AaePIWI3 and AaePIWI4. The amino acid substitution probability among these is low relative to the other 2 clades, (0.1943; CV = 39.5%). Members of the Ago4-like group were distinguished by the synapomorphy "ETGIQVLNLILRRAMNGLNLQLVGRNLY" within the first 260 amino acids (Additional File 2). This synapomorphy is maintained in DmePIWI, but not in DmeAub. Members of the Ago5-like group have a variable sequence in the corresponding region.

AgaAgo5 is basal to the Ago5-like clade, which has 93.0% bootstrap support and contains CpiPIWI4, CpiPIWI5, CpiPIWI6, AaePIWI5, AaePIWI6 and AaePIWI7. The amino acid substitution probability is high (0.3422; CV = 37.8%). These analyses indicate that the expanded PIWI protein subfamily comprises several different gene families with some resulting from recent gene duplication events (Figure 4, Additional File 1E). Expansion of Argonaute family proteins has occurred in other organisms, as well. One example is in *C. elegans*, in which a gene expansion has occurred, evidently to aid in sequential amplification of siRNA signal. A key difference between the *C. elegans *and mosquito Argonaute family gene expansions is that the *C. elegans *secondary Agos (SAGOs) lack the key metal binding residues required for RNase H catalytic activity and so are thought to play a secondary role in RNAi [58], whereas, these key catalytic residues are present in mosquito PIWIs (Figure 2).

In drosophilids, PIWI pathway components have been implicated in the control of both retrotransposons and the long terminal repeats of telomeres [31,33]. Our analysis suggests the PIWI subfamily gene expansion initially occurred in ancestral Culicinae mosquitoes before the divergence of *Ae. aegypti *and *Cx. pipiens *ancestors [59,60]. Expansions in PIWI pathway components may have been adaptive for controlling the increased burden of retrotransposons in *Ae. aegypti *and *Cx. pipiens *relative to *An. gambiae*. Although retrotransposons are present in the *An. gambiae *genome, the percentage of the genome bearing TEs is much less than that of *Ae. aegypti *[46,61-63]. About 47% of the *Ae. aegypti *genome harbors both class I and class II transposable elements (TEs), whereas the *An. gambiae *genome contains about 16% TEs [46,51]. TEs are also present in *Culex spp*. genomes, however the percentage of the genome occupied has not yet been reported [63,64]. Retrotransposons, also known as Class I TEs, account for at least half of the TE load in both anopheline and aedine genomes. Therefore, the aedine genome carries more than twice the retrotransposon load than anophelines carry.

In a related context, DmePIWI has also been implicated in maintenance of heterochromatin. DmePIWI associates directly with chromatin and heterochromatin protein 1a (HP1a) [65]. DmePIWI-HP1a interactions occur through conserved PIWI protein motifs, PxVxV or PxVxM. Of the mosquito PIWIs, only CpiPIWI4A, CpiPIWI4B, AaePIWI5 and AaePIWI6 contain conserved PxVxV sites, and no proteins bear PxVxM motifs (data not shown). Importantly, all four of these proteins are in the Ago5-like protein class. In contrast, neither AgaAgo4 nor AgaAgo5 carry these motifs.

### Accessory Proteins

The RNA helicases of *Drosophila *and mosquitoes are clearly a large and complex family. Other than a genetic connection to anti-viral defense and retrotransposon maintenance in drosophilids, little is known about Rm62 in Dipterans. Using a high stringency search cut-off E value of E = 10^-80^, multiple mosquito genes were identified that are homologous to *DmeRm62 *RNA helicase (Figure 1 and Additional Files 1A, F). Although reciprocal genome searches identified 7 homologous RNA helicase genes in *Drosophila*, only DmeRm62 has been linked to RNA interference [24,66]. Phylogenetic analysis demonstrated that there are independent orthologous groups of Rm62-like proteins (Additional File 3).

Rm62 and Armitage-like proteins carry predicted DEAD box ATP-dependent helicase domains. DmeArmitage is a transcriptional repressor of specific mRNAs during male germ-line development and has been shown in a genetic screen to be involved in anti-viral defense [24,67]. Interestingly, the Armitage gene duplication in *Aedes *and *Culex *has resulted in two different types of multi-domain helicases (Figure 1). Although both aedine Armitage-like paralogs carry a type III DNA restriction enzyme domain, as does DmeArmitage, only one of the two *Culex *paralogs carries this domain (CPIJ001247). Instead, the second paralog is missing the restriction enzyme domain and carries a predicted DNA helicase domain (CPIJ001245) (data not shown). In contrast, AgaArmitage has two RNA helicase domains, in addition to the DNA restriction enzyme domain. This interesting diversity in protein domains among mosquito species suggests that, if Armitage participates in anti-viral defense as in *D. melanogaster*, it may be pivotal to species-specific differences.

### Upstream Control Regions

We took a conservative approach to determine whether cis-acting elements are conserved among SRRP component upstream regions. Our goals were two-fold: 1) to identify previously validated mosquito TFBSs and 2) novel upstream regulatory control elements in regions corresponding to -1000 nts to +100 nts. This region corresponds to the upstream genomic sequence, the 5' un-translated region of the transcript, the translation start site and some coding nucleotides in the first exon. Consensus sequences for the upstream motifs are listed in Table 2. We determined whether elements in each of these categories are conserved within each pathway or across mosquito species.

**Table 2:** UCR elements used in this Study

GATA	See Additional File 4	[78,79]
NFkB1	GGKGATYYAC	Aedine consensus [72]
NFkB2	KGGGAWHMMM	Anopheline consensus [72]
NFkB4	KGKGAWHHMM	Cross-species consensus
NFkB7	GGGAWHM	Subset of NFkB-related TFBSs, analyzed with higher stringency
JAK-STAT	TTCTAGGAA, TTTCTAAGAAA	[69,95]
BR-C Z1	TAAWWRACAARW	[96]
BR-C Z2	TTWWCTATTT	[96]
BR-C Z3	WAAACWWRW	[96]
BR-C Z4	RKAAASA	[96]
M1	MSMYCCACCCMCTCC	Aedes-specific
M2	SMCWCCCACCYMCYC	Aedes-specific
M7	RCMRCARCARCARCA	Anopheles-specific
M8	SCRMMRCAGCARCAGCARCM	Anopheles-specific
M9	KTGTGTGTGTGTKYG	Anopheles-specific
M12	AAGAATTTAAGAATT	Culex-specific
M13	TTYTTAAATTYTYAA	Culex-specific
M17	AACTTTTT	Culex-specific
M19	CCCMCCCCCCCCCYYY	miRNA pathway-specific
M21	SRGBSGCSGKRSSGG	miRNA pathway-specific
M24	CCRCMRCARCMGCWRCMRCM	siRNA pathway-specific
M26	TYTGAG	siRNA pathway-specific
M30	CGAGCKRCTSSRASYWGGTT	PIWI pathway-specific

IUPAC consensus codes used, wherein "K" is T or G; "Y" is T or C; "W" is T or A; "R" is A or G; "S" is C or G; "M" is C or A; "H" is A, T, or C; "V" is A, C or G; "B" is C, T, or G; "D" is A, T, or G.

Within the last 150 million years, *An. gambiae *and *Ae. Aegypti *became distinct species, and more recently, *Cx. pipiens *diverged. One might expect that among-species comparisons would yield fewer common elements than within-species elements. However, non-coding DNA is subject to the same stabilizing selection as protein coding regions, and, in drosophilids, non-coding DNA is actually less polymorphic than protein coding regions at synonymous sites [68]. If purifying selection has occurred in UCRs of mosquito SRRP genes, common elements could be conserved across species, thus adding support to the functional significance of upstream regulatory elements, regardless of whether they were unique across the entire genome. Furthermore, selective pressures on non-coding DNA can be significant and therefore exacerbate the search for TFBSs [68]. Therefore, to maintain a conservative line of inquiry, we chose to examine only those TFBSs that have been experimentally confirmed in mosquitoes. The search for novel motifs was expected to yield conserved cis-acting regulatory elements.

### Transcription factor binding sites: NF-kappa B related sites

**Figure 5:**
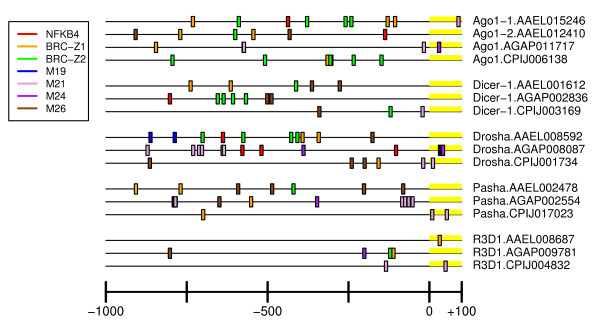
**miRNA pathway UCR Schematic**. Graphical representation of key cis UCR elements of miRNA pathway components. Scale is 1000 bases upstream to about 100 bases downstream of the beginning of the transcript.

**Figure 6:**
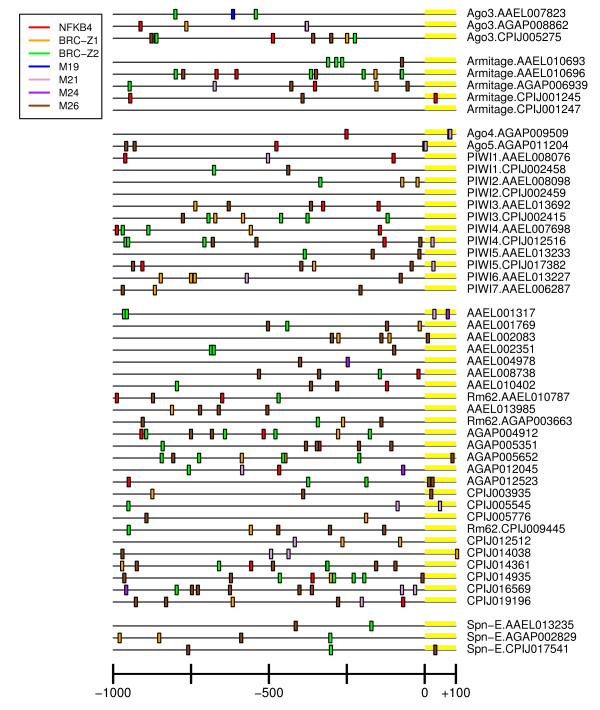
**PIWI pathway UCR Schematic**. Graphical representation of key cis UCR elements of PIWI pathway components. Rm62-like genes list the accession number without any additional annotation. Scale is 1000 bases upstream to about 100 bases downstream of the beginning of the transcript.

**Figure 7:**
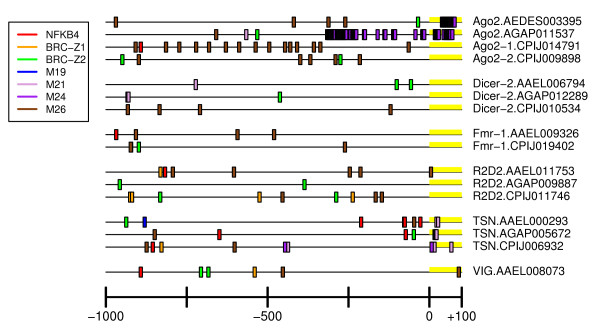
**siRNA pathway UCR Schematic**. Graphical representation of key cis UCR elements of siRNA pathway components. Scale is 1000 bases upstream to about 100 bases downstream of the beginning of the transcript.

There is empirical evidence that RNA viruses activate innate immunity pathways in Dipterans. The Toll and JAK-STAT (janus kinase/signal transducers and activators of transcription) pathways are activated in drosophilids upon infection with *Drosophila *C virus or *Drosophila *X virus [69,70]. A search for STAT binding motifs (Table 2) among all pathway component UCRs yielded no matches, thus suggesting that the JAK-STAT pathway does not activate transcription of effectors of mosquito miRNA, siRNA, or PIWI pathways. Mosquito NF kappa B-related proteins, REL1 and REL2, participate in transcriptional induction of immune effectors during bacterial and fungal infections (summarized in [71]). REL1 stimulates downstream effectors via the Toll pathway, while REL2 stimulates downstream effectors via the immunodeficiency (Imd) pathway [72]. During Sindbis (SINV) infection of *Ae. aegypti*, *REL1 *transcripts are enriched early in infection [73]. Identification of REL TFBSs in UCRs of SRRP genes would suggest a link between small regulatory RNA pathways and the innate immune response cascade. Consensus NF kappa B-related binding motifs in *Ae. aegypti *and *An. gambiae *are "GGKGATYYAC" (NFkB1) and "KGGGAWHMMM" (NFkB2), respectively. A cross-species consensus is "KGKGAWHHMM" (NFkB4) [72]. These REL TFBS consensus motifs could be bound by either AaeREL1 or AaeREL2. Search of all mosquito SRRP components for NFkB1 yielded hits on only two Rm62-like UCRs, and NFkB2 revealed few hits across all species (Additional File 4). The cross-species 10 nt consensus, NFkB4, revealed more hits, some of which are redundant to those identified by NFkB2 (Figures 5, 6, 7). To identify putative motifs under more relaxed conditions, a shortened version of the NFkB2 consensus, "GGGAWHM" (NFkB7), was used. In this case, the relaxed search was followed by a more rigorous requirement of ≥ 2 motifs per UCR. Proportions of UCRs containing these motifs are listed in Table 3.

**Table 3:** Selected UCR Motifs

				**Proportion of UCRs with Motif**			**Median Motifs per UCR**		
Motif	FET p value	BH	Enriched over genome?	miRNA	siRNA	PIWI	miRNA	siRNA	PIWI
NFkB2	not sig.^	not sig.	No	0.188	0.250	0.100	1.0	1.0	1.0
NFkB4	not sig.	not sig.	No	0.313	0.438	0.460	1.0	1.0	1.0
NFkB7*	not sig.	not sig.	No	0.188	0.375	0.280	1.0	1.0	1.0
BRC_Z1	not sig.	not sig.	No	0.813	0.313	0.540	1.0	1.0	1.0
BRC_Z2	not sig.	not sig.	No	0.563	0.688	0.620	1.0	1.0	1.0
Pathway-specific							miRNA	siRNA	PIWI
M19 (miRNA)	not sig.	not sig.	Yes	0.188	0.100	0.033	4.5	1	1
M21 (miRNA)	not sig.	not sig.	Yes	0.219	0.100	0.067	2	2	1
M24 (siRNA)	not sig.	not sig.	Yes	0.063	0.400	0.200	1	14	1
M26 (siRNA)	not sig.	not sig.	Yes	0.031	0.250	0.033	2	3	2
M30 (PIWI)	not sig.	not sig.	Yes	0.000	0.000	0.060	NA	NA	5
Species-Specific				Aae	Aga	Cpi	Aae	Aga	Cpi
M1 (Aedes)	not sig.	not sig.	Yes	0.188	0.100	0.033	1.5	1.5	1
M2 (Aedes)	not sig.	not sig.	Yes	0.219	0.100	0.067	2	2	1
M3 (Aedes)	not sig.	not sig.	Yes	0.094	0.000	0.033	9	NA	1
M7 (Anopheles)#	not sig.	not sig.	Yes	0.063	0.400	0.200	10.5	1.5	1
M8 (Anopheles) #	not sig.	not sig.	Yes	0.031	0.250	0.033	10	1	1
M9 (Anopheles)	< 0.001	0.002	No	0.000	0.400	0.300	NA	6	1
M12 (Culex)+	not sig.	not sig.	Yes	0.031	0.000	0.067	1	NA	21
M13 (Culex)+	not sig.	not sig.	Yes	0.031	0.000	0.067	1	NA	21
M17 (Culex)	< 0.001	0.002	No	0.094	0.100	0.533	1	1	1

"^", see Additional File 4 for details. "*", for NFkB7 only, significance scores were based on the presence of 2 or more hits per UCR. "#", enriched in an siRNA pathway specific manner. "+", enriched in an miRNA pathway specific manner. "BH", Benjamini-Hochberg test, cut-off = 0.05. "FET", Fisher's Exact test, cutoff = 0.01. "NA", not applicable. "Aae", *Ae. aegypti*, "Aga", *An. gambiae*, "Cpi", *Cx. pipiens*.

Schematics showing distribution of NFkB4 motifs are shown in Figures 5, 6 and 7. Hits were detected in key component UCRs of all three mosquito species and SRRPs. For example, *AaeDrosha*, *AgaDrosha*, *AaeTSN, CpiTSN *and *AgaTSN *UCRs share NFkB4 sites, and all three species have NFkB4 sites upstream of PIWI pathway component UCRs. Although there were no significant pathway-specific or species-specific differences in the presence of NF kappa B-related binding sites, the presence of these motifs in key UCRs suggests a possible link to the innate immune response or development.

The drosophilid ortholog of Tudor staphylococcal nuclease (TSN) is a component of the RISC and has multiple functional attributes, such as transcriptional co-activation activity, non-specific ssRNA cleavage, cleavage of hyper-edited dsRNA substrates, and an un-defined role in anti-viral defense [43,74]. Microarray analysis of *Ae. aegypti *transcripts during a SINV infection, showed early enrichment of *AaeREL1 *transcripts at 1 day post-infection (dpi), followed by stimulation of *AaeTSN *at 4 dpi [73]. Analysis of anti-viral RNAi component transcript levels in *Ae. aegypti *infected with alphaviruses or flaviviruses showed periodic enrichment of *TSN *transcripts in a virus-dependent manner [25](Campbell, unpublished). Importantly, *TSN *UCRs from all three species contained NFkB4 motifs (Figure 7, Additional File 4). This evidence further suggests that a link exists between the anti-viral pathway and the innate immune response pathway of mosquitoes.

### Broad Complex motifs

Broad complex (BRC) transcription factors act during ecdysone-dependent regulation of gene expression in invertebrates [75]. Ecdysone, a steroid hormone, is of key importance in embryogenesis and development in insects. Of the four types of BRC TFBSs, BRC_Z1 is the most abundant in miRNA and piRNA pathway component UCRs, however, the enrichment is not statistically significant (Table 3). In mosquitoes, BRC_Z1 is thought to be a transcriptional repressor that ensures proper temporal gene expression control for the yolk protein precursor gene, vitellogenin [76]. The presence of a BRC_Z1 motif upstream of nearly all of the miRNA pathway components and many PIWI pathway components suggests the requirement for temporal control of these pathways, as well. BRC_Z2 is required for 20-hydroxyecdysone-mediated transcriptional activation [76]. BRC_Z2 motifs were identified on key genes of all three pathways, sometimes in tandem with BRC_Z1 motifs (Figures 5, 6, 7 and Additional File 4).

### GATA factors

GATA factors control tissue- and temporally-specific gene expression. GATA binding sites are upstream of a variety of genes in mosquitoes, including lysosomal protease, gut trypsin, and vitellogenin genes [77-79]. Expression of gut trypsins and vitellogenin genes are tightly linked to bloodfeeding. GATA factors or GATA repressors bind these sites to either activate or repress transcription. Multiple GATA sites were identified in all UCRs, indicating no obvious species-specific or pathway-specific variation (Additional File 4). Therefore, this element was not analyzed further.

### Novel Elements

Novel UCR elements could serve as genomic structural elements, mRNA stability elements or TFBSs. We identified features in upstream non-coding regions that may have been selectively maintained over the course of evolution. To accomplish this, we compared UCRs across mosquito species and across small RNA regulatory pathways for all three mosquito species. This focused approach allowed us to use a subset of each mosquito genome as background. The presence of *cis *upstream elements across all three mosquito species for a single pathway would suggest that they may be important regulatory features of that pathway. Although some components, such as those of the RISC, may act in multiple pathways; for our analysis, we categorized the groups according to the outline in Figure 1. The sequence motif search programs MEME (Multiple EM for motif elicitation), MDScan, and Weeder were used [80-83]. These programs identify over-represented sequence patterns or motifs in a given dataset compared to all other datasets. The top two elements discovered for each species and pathway were then identified among all UCRs, and significant differences among pathways or species were noted. Selected motifs are listed in Table 3, along with the proportion of UCRs with at least one species-specific or pathway-specific hit, and full descriptions are in Additional File 4. Fisher's Exact test was used to determine whether the UCR elements or TFBS motifs were enriched in a pathway-specific or species-specific manner. The Benjamini-Hochberg (BH) multiple testing adjustment was used to correct for false positives, thus, for any BH score over 0.05, the Fisher's Exact test score was considered insignificant. In addition, we performed full genome searches for all novel elements and removed those that did not show enrichment in SRRP UCRs over the rest of the genome (Additional File 4). Together, these methods allowed identification of *cis*-elements that could be important regulatory features of SRRP pathways.

Identification of species-specific *cis *elements could provide an important foundation for future characterization of species-specific differences in regulation of pathogen defense pathways. By convention, strong regulatory sites are those represented in tandem repeats (Figures 5, 6, 7, Additional File 4) [84]. Due to the experimental design, elements that were identified in the species-specific search might also be enriched in a pathway-specific manner. For example, the *An. gambiae *elements M7 and M8 are enriched over the remainder of the genome in an siRNA pathway-specific manner for both *An. gambiae *and *Ae. Aegypti *(Additional File 4). In addition, elements M12 and M13 are enriched in *Cx. pipiens *in a miRNA pathway-specific manner over the remainder of the genome, even though Fisher's Exact test did not indicate the *Cx. pipiens *has significantly more of these elements than *Ae. aegypti *or *An. gambiae *(Table 3). In contrast, the M1 and M2 elements of *Aedes aegypti *are strongly enriched in all SRRPs over the remainder of the aedine genome (Additional File 4). M9 (*An. gambiae*) and M17 (*Cx. pipiens*) were enriched in a species-specific manner over all other SRRP UCRs according to Fisher's Exact test (Table 3), however, they were not enriched in SRRP upstream regions compared to the remainder of the genome. Therefore, they are not likely to be important to regulation of small RNA metabolism.

Pathway-specific upstream elements were also identified. Of the five elements identified, M24 was the most interesting, because, it was enriched among the siRNA pathway UCRs across all three species. Conservation of within-pathway features across species supports the hypothesis of purifying selection in UCRs of small RNA regulatory components.

### Implications for Vector Competence

Of the Argonaute protein subfamily members, Ago2, the anti-viral RNase H-type nuclease, is significantly more diverse among mosquito species than the miRNA pathway nuclease, Ago1. This finding suggests that anti-viral defense effectors are evolving at a faster rate than those involved in housekeeping functions. Anti-viral defense systems likely evolved to protect against entomopathogenic viruses. In turn, some entomopathogenic viruses probably evolved into arboviruses. When considering these pathways, it is important to keep in mind that evolutionary pressure exists on both the arbovirus and the vector mosquito to modulate the immune response.

Differences in UCR element motif patterns suggest that there are likely to be species-specific differences in transcriptional regulation of siRNA pathway components that could affect arbovirus vector competence. Loosely categorized, *An. gambiae *primarily transmits malaria parasites, *Cx. pipiens *transmits encephalitic arboviruses, and *Ae. aegypti *transmits important hemorrhagic arboviruses. For a given arthropod, low vector competence could arise from an effective immune response that clears the pathogen and prevents pathogen escape to the salivary glands. With this model in mind, we hypothesize that *An. gambiae *has a more effective antiviral immune response than either *Cx. pipiens *or *Ae. aegypti*. Characterization of these putative regulatory differences awaits further exploration in each species. The demonstrated ability of some mosquito species to serve as competent arbovirus vectors in spite of the presence of anti-viral RNAi components, begs the question of what mechanisms are used by arboviruses to evade anti-viral RNAi defense.

This report brings to bear both the importance of PIWI family gene expansion in vector mosquitoes and the need for research focus in this area. A few exploratory studies have found evidence that PIWI proteins could be important in anti-viral defense. Transient silencing of *Ago3 *by dsRNA injection increased O'nyong-nyong infection of *An. gambiae *[1]. However, characterization of PIWI pathway activity in mosquito anti-viral defense is clearly required before additional conclusions can be drawn. In *Drosophila*, an apparent paradox is found, wherein PIWI pathway proteins are reportedly expressed only in ovaries and are restricted to germline maintenance, however, PIWI pathway components DmeAub, DmeArmitage and DmeRm62 have been implicated in anti-viral processes [5,24,67,85]. Recent reports describing the presence of endogenous siRNAs (endo-siRNAs) are beginning to shed light in this area [29,30]. Small RNAs were found to control retrotransposons in somatic tissue in *D. melanogaster *in a Dicer2/Ago2 dependent manner.

## Conclusion

This genomics study provides important contextual information for both vector biologists and the RNAi community and highlights important differences between vector mosquitoes and model organisms, such as *Drosophila*. Together, these data suggest a need to further investigate the relationship between vector competence and specific small RNA pathways across mosquito species, as well as potential arbovirus strategies for evasion of these defense mechanisms. However, it is still unclear what aspects of mosquito biology are driving evolution and gene expansion of Argonaute family genes.

## Methods

### Putative ortholog identification

Mosquito Argonaute/PIWI family genes were identified by tblastx search of the *Ae. aegypti *and *Cx. pipiens quinquefasciatus *genomes at Vectorbase.org, using the *An. gambiae *RNAi orthologs [86]. Putative orthologs of other SRRP components were identified by tblastx search of the databases using drosophilid RNAi genes. Argonaute and Rm62 family hits with E values of 0.0 -10^-80 ^were included in the analyses. Many other putative orthologs did not allow cut-offs with this high stringency, therefore, for all other groups, top hits with E values of < 10^-40 ^were used. Gene expression in each species was corroborated by the presence of a mosquito EST in the public database, as determined by blastn search, E value < 10^-40^. Putative isoforms from *Cx. pipiens*, based on alternate gene predictions at the same locus, were designated with a letter following the allele number (Additional File 1A).

The conservative naming conventions used for the PIWI genes could not be followed for Ago1 and Ago2. The gene names "Argonaute 1" and "Argonaute 2" are understood in the broad scientific community to be associated with the miRNA and siRNA pathways, respectively. To use a different numbering scheme for the expanded "Argonaute 1" and "Argonaute 2" gene families would have further added to confusion, therefore, we chose to assign the newly identified genes as paralogs rather than orthologs, even though the genes were not located on the same super-contig. The high level of sequence similarity provides additional support for this decision. For example, the open reading frames of AaeAGO1-1 and AaeAGO1-2 have 99.1% nucleotide identity but different upstream flanking sequences and introns. Similar searches were done for CpiAGO2-1A and CpiAGO2-2. Importantly, CpiAGO2-1A and CpiAGO2-2 share only 48% nt identity but both are classified as 'Ago2-type' proteins according to amino acid sequence similarities and synapomorphies.

### Phylogenetic analyses

Full-length PIWI subfamily proteins were compared. The C-terminal 250 amino acids of Argonaute subfamily proteins were compared. Partial Ago sequences were used, because full-length sequences were not available for some species. A Gonnet matrix was used in a CLUSTALW alignment; this weighted matrix is based on the supposition that any given amino acid substitution is influenced by neighboring amino acids, and thus provides a basis for characterizing protein families [87]. The alignment was used to generate a maximum likelihood phylogenetic tree using PROML (PHYLIP [88]). Phylogenetic relationships among proteins were estimated using a maximum likelihood analysis of amino acid sequences with the Jones-Taylor-Thornton probability model of amino acid changes [55]. This method assumes that taxa evolve independently, that each amino acid position evolves independently and that substitutions at each amino acid site occur with a probability specified in the PAM 250 matrix. In addition, all amino acids are included in the analysis rather than just the phylogenetically informative sites. A bootstrap analysis was done with 1,000 pseudoreplicates.

### Motif Searches and Analyses

Genomic sequences corresponding to approximately -1000 to +100 was extracted from genomic, 5' untranslated region, and partial coding region of each gene as listed at Vectorbase.org [86]. Motif searches were performed using MEME, Weeder, and MDScan [81-83,89-91]. Searches of the upstream control regions were performed separately for each of the following groups: (1) *Ae. aegypti*, (2) *An. gambiae*, (3) *Cx. pipiens*, (4) miRNA genes across species, (5) siRNA genes across species and (6) PIWI genes across species. The top two consensus sequences from each of the searches were included in subsequent analyses.

String analysis (R Biostrings, version 2.4.8) was used to locate and count the number of matches for each of the novel motifs and the previously described GATA, BRC Z1–Z4 and NFkB motifs [92]. The consensus sequences and the number of mismatches allowed for each motif are shown in Additional File 4. Motifs present multiple times in all UCRs were removed from further analysis. In addition, NFkB1 was removed from further consideration because it was not contained in any UCRs.

Full genome searches were performed to ensure that each element is enriched in the pathway or species of interest over the rest of the genome. This validation method provided support for the hypothesis that the particular motif was enriched in the pathway-specific or species-specific manner in which it was originally discovered. Each species genome was searched for the given sequence using the same number of mismatches denoted in Additional File 4. The output number was adjusted to the number of occurrences per supercontig length. The 95^th ^percentile of this "mod count" was used as a cut-off to determine which motifs were enriched in each category. Motifs reported as species-specific were enriched at or above the 95% percentile for the species in which it was identified.

Motifs reported as pathway-specific were enriched at or above the 95% percentile for the pathway in which it was identified for at least two of the three species. Experimentally identified motifs are interesting for biological reasons, therefore, significance by Fisher's Exact test or genome search was not required.

Fisher's Exact test adds further support and highlights motifs that are over-represented in a species or pathway-specific manner among all UCRs tested. The median number of hits was calculated using only those UCRs which contained at least one hit. Fisher's exact test was used to test for a relationship between each pathway (or species) versus the absence or presence for each motif, using a lower cut-off of one hit per motif. The Benjamini-Hochberg (BH) multiple testing adjustment method, which controls the False Discovery rate, was also performed, using a cut-off of 0.05 [93]. This cut-off ensures that 5% or fewer should be false discoveries or false positives and was used to identify motifs with significantly different proportions among pathways or species. The BH adjusted p-values as well as the gene proportions for which each motif was observed at least once are shown in Table 3.

## Authors' contributions

CLC designed the study, performed bioinformatics analyses and wrote the manuscript. WCB4 performed the phylogenetic analyses and contributed to the manuscript. AH performed the UCR searches, summarized the results and carried out the statistical analyses. BDF edited the manuscript. All authors read and approved the manuscript.

## Supplementary Material

Additional File 1 **SRRP components in vector mosquitoes compared to drosophilids**. SRRP homologs, Argonaute Family and Rm62-like Protein alignments. Click here for file

Additional File 2 **Argonaute Protein Family synapomorphies**. Signature protein features of protein classes. Click here for file

Additional File 3 **Rm62-like Protein Tree**. Maximum Likelihood tree of Mosquito Rm62-like proteins compared to Drosophila Rm62-like helicases. Click here for file

Additional File 4 **UCR motif statistical analyses**. Full genome search data; locations of elements in each UCR. Click here for file
