# Supplementary material for: Comparative genomics of small RNA regulatory pathway components in vector mosquitoes
Source: BMC Genomics. 2008 Sep 18;9:425. doi: 10.1186/1471-2164-9-425 (PMC2566310; doi:10.1186/1471-2164-9-425)
Supplement: Additional File 1 — SRRP components in vector mosquitoes compared to drosophilids. SRRP homologs, Argonaute Family and Rm62-like Protein alignments. [file 1471-2164-9-425-S1.pdf]

Additional File 1A. SRRP components in vector mosquitoes compared to drosophilids

Pathway	Gene Name	D. melanogaster	Chr	Protein^, a.a.	An. gambiae	Chr	Protein
miRNA	Dicer-1	NM_079729[b]	3R	2249	AGAP002836 [a]	2R	2256
	Loqs (R3D1)	NM_001038814	2L	383	AGAP009781	3R	341
	Drosha	NM_058088	2L	1327	AGAP008087	3R	1404
	Pasha	NM_143622	3R	642	AGAP002554	2R	648
	Ago1	NM_166020	2R	985	AGAP011717	3L	981
siRNA	Dicer-2	NM_079054	2R	1722	AGAP012289	3L	1672
	VIG	NM_078848	2L	490	[c]		
	TSN	NM_138177	3L	926	AGAP005672	2L	919
	R2D2	NM_135308	2L	311	AGAP009887	3R	324
	Fmr-1	NM_137801	3R	684	[c]		
	Ago2B	NM_140518	3L	1214	AGAP011537	3L	933[d]
	Ago2C	NM_168626	3L	1217	n/a		
PIWI	Armitage	NM_139559	3L	1274	AGAP006939	2L	1223
	Spindle-E	NM_057393	3R	1434	AGAP002829	2R	1379
	Rm62-like	NM_169118	3R	578	AGAP005652	2L	728
		NM_132792	X	1224	AGAP003663	2R	746
		NM_132196	X	945	AGAP004912-PA	2L	679
		NM_139805	3L	818	AGAP005351	2L	640
		NM_165103	2L	661	AGAP012045	3L	910
		NM_141510	3R	703	AGAP012523	UKN	720
		NM_140752	3L	594			
	Ago3	EF211827	n/a	687	AGAP008862	3R	930
	PIWI	NM_057527	2L	843	Ago4 AGAP009509	3R	889
	Aub	NM_057386	2L	866	Ago5 AGAP011204	3L	843

Additional File 1A continued

Pathway	Gene Name	Ae. aegypti	Super-Contig	Protein, a.a.	Cx. pipiens quinquefasciatus	Super-Contig	Protein a.a.
miRNA	Dicer-1	AAEL001612	1.37	2193	CPIJ003169	3.43	2270
	Loqs (R3D1)	AAEL008687	1.342	329	CPIJ004832	3.76	390
	Drosha	AAEL008592	1.334	1374	CPIJ001734	3.25	835
	Pasha	AAEL002478	1.58	564	CPIJ017023	3.875	683
	Ago1	(Ago1-1) AAEL015246	1.1658	825	CPIJ006138	3.109	964
		(Ago1-2) AAEL012410	1.693	947	n/a		
siRNA	Dicer-2	AAEL006794	1.221	1658	CPIJ010534	3.278	1665
	VIG	AAEL008073	1.299	419	[c]		
	TSN	AAEL000293	1.5	921	CPIJ006932	3.137	922
	R2D2	AAEL011753	1.608	318	CPIJ011746	3.344	314
	Fmr-1	AAEL009326	1.389	717	CPIJ019402	3.1587	738
	Ago2	SUPP_AEDES003395	1.89	992	(Ago2-1A) CPIJ014791 [d]	3.588	864
					(Ago2-1B) CPIJ014791 [d]	3.588	924
					(Ago2-2) CPIJ009898 [d]	3.250	974
PIWI	Armitage	AAEL010693	1.497	1048	CPIJ001245	3.15	989
		AAEL010696	1.497	699	CPIJ001247	3.15	638
	Spindle-E	AAEL013235	1.809	1374	CPIJ017541	3.940	1396
	Rm62-like	AAEL001317	1.29	799	CPIJ003935	3.61	409
		AAEL001769	1.42	718	CPIJ005545	3.114	942
		AAEL002083	1.48	699	CPIJ005776	3.100	709
		AAEL002351	1.54	639	CPIJ009445	3.239	579
		AAEL004978	1.137	638	CPIJ012512	3.379	935
		AAEL008738	1.345	911	CPIJ014038	3.24570	572
		AAEL010402	1.476	741	CPIJ014361	3.538	798
		AAEL010787-PA	1.508	594	CPIJ014935	3.584	686
		AAEL013985	1.976	1029	CPIJ016569	3.896	663
					CPIJ019196	3.1491	538
	Ago3	AAEL007823	1.284	944	CPIJ005275 [d]	3.94	847
	Ago4-like	PIWI1 AAEL008076	1.300	674	PIWI1 CPIJ002458 [d]	3.32	881
		PIWI2 AAEL008098	1.300	875	PIWI2 CPIJ002459 [d]	3.32	870
		PIWI3 AAEL013692	1.896	882	PIWI3A CPIJ002415 [d]	3.32	865
		PIWI4 AAEL007698	1.275	860	PIWI3B CPIJ002415 [d]	3.32	883
	Ago5-like	PIWI5 AAEL013233	1.809	883	PIWI4A CPIJ012516 [d]	3.379	880
		PIWI6 AAEL013227	1.809	888	PIWI4B CPIJ012516 [d]	3.379	871
		PIWI7 AAEL006287	1.199	945	PIWI5A CPIJ017382 [d]	3.997	857
					PIWI5B CPIJ017382 [d]	3.997	802
					PIWI6 CPIJ017381 [d]	3.997	905

Additional File 1A. "^", Predicted translation product. **"[a]",** Accession numbers are from Vectorbase.org [89]. "[b]", Accession numbers are from Genbank. "**[c]**", ortholog not found. "[d]", The accession number refers to the genomic locus, however, alternate coding sequences were used. These have been submitted to Vector Base and will be added to a future genebuild; they may be referenced by referring to the gene name and genome location (Additional File 1G). "n/a", not applicable. Gray shading indicates presence of mosquito EST in public database, as determined by blastn search, E value <-10^40. Gray-shaded CpiAgo transcripts were confirmed by RT-PCR. Loci with alternate splice variants were designated with a letter suffix (ie., PIWI3A).

Additional File 1B-F. Argonaute Family Protein alignments

1B. Ago1 protein alignment

```
Ago1aga    MSTERELTPGTTQQLHPLSYSDMATHIQLNG--VIMGKSFNESPWTSSPPRPPSPSQSQTSFDTLSRKLP
Ago1cpi    .TMN-------------.GP..ITSTEHAVD--AFL.--------------------TT.ELTD.TIQ.D
Ago1-1aae  ----------------------------------------------------------------------
Ago1-2aae  ----------------------------------------------------------------------
Ago1dme    .......A..GPA....HTLPLTFPDL.MTSTVG.I..VY-..Q..P..T..Q....A.......TSPPA

Ago1aga    ALSSCLPTAIKNGSAALSLPRFSPPAGATVNPTTVATTTGTQG--AQALGVVPATPPAPPDLPVFTCPRR
Ago1cpi    .TTQRR-D..RKERK.K---.AA.........-.PT..S.A.NVATS.......................
Ago1-1aae  ----------------------------------------------------------------------
Ago1-2aae  ----------------------------------------------------------------------
Ago1dme    PG..VN...VTSP..QNVAAGGATV...AATAAQ..SAL.ATTG-SVTPAIAT...ATQ..M........

Ago1aga    PNLGREGRPIVLRANHFQITMPRGFVHHYDINIQPDKCPRKVNREIIETMVHAYSKMFGALKPVFDGRNN
Ago1cpi    ......................................................................
Ago1-1aae  --------------------..................................................
Ago1-2aae  --------------------..................................................
Ago1dme    ..................V.....Y...............................I..V..........

Ago1aga    LYTRDLLPIGNDRVELEVTLPGEGKDRVFRVTIKWVAQVSLFNLEEALEGRTRQIPYDAILALDVVMRHL
Ago1cpi    .....P................................................................
Ago1-1aae  .....P................................................................
Ago1-2aae  .....P................................................................
Ago1dme    .....P.....E.L.............I.......Q..................................

Ago1aga    PSMTYTPVGRSFFSSPDGYYHPLGGGREVWFGFHQSVRPSQWKMMLNIDVSATAFYKAQPVIEFMCEVLD
Ago1cpi    ......................................................................
Ago1-1aae  ......................................................................
Ago1-2aae  ......................................................................
Ago1dme    ................E.............................................D.......

Ago1aga    IRDINEQRKPLTDSQRVKFTKEIKGLKIEITHCGTMRRKYRVCNVTRRPAQMQSFPLQLENGQTVECTVA
Ago1cpi    ......................................................................
Ago1-1aae  ......................................................................
Ago1-2aae  ......................................................................
Ago1dme    ..................................Q...................................
```

```
Ago1aga     KYFLDKYKMKLRYPHLPCLQVGQEHKHTYLPLEVCNIVAGQRCIKKLTDMQTSTMIKATARSAPDREREI
Ago1cpi     ......................................................................
Ago1-1aae   ......................................................................
Ago1-2aae   ......................................................................
Ago1dme     .......R..............................................................

Ago1aga     NNLVRRADFNNDAYVQEFGLTISNNMMEVRGRVLPPPKLQYGGRVSS-MSGQLLSGPQNKVSLALPNQGV
Ago1cpi     ....................A...S......................-.....P................
Ago1-1aae   .S..................A...S......................-......................
Ago1-2aae   .S..................A...S......................-......................
Ago1dme     ....K.......S...........S.....................TGLT..Q.FP........S.....

Ago1aga     WDMRGKQFFTGVEIRVWAIACFAPQRTVREDALRNFTQQLQKISNDAGMPIIGQPCFCKYATGPDQVEPM
Ago1cpi     ......................................................................
Ago1-1aae   ......................................................................
Ago1-2aae   ......................................................................
Ago1dme     ...............I......................................................

Ago1aga     FRYLKSTFSHLQLVVVVLPGKTPVYAEVKRVGDTVLGMATQCVQAKNVNKTSPQTLSNLCLKINVKLGGI
Ago1cpi     .....N..NQ............................................................
Ago1-1aae   .....N..NA............................................................
Ago1-2aae   .....N..NA............................................................
Ago1dme     .....I..PG............................................................

Ago1aga     NSILVPSIRPKVFDEPVIFLGADVTHPPAGDNKKPSIAAVVGSMDAHPSRYAATVRVQQHRQEIIQELSS
Ago1cpi     ......................................................................
Ago1-1aae   ......................................................................
Ago1-2aae   ......................................................................
Ago1dme     .............N........................................................

Ago1aga     MVRELLIMFYKSTGGFKPHRIILYRDGVSEGQFPHVLQHELTAIREACIKLEADYKPGITFIVVQKRHHT
Ago1cpi     ......................................................................
Ago1-1aae   ......................................................................
Ago1-2aae   ......................................................................
Ago1dme     ...............Y....................................PE.R..............

Ago1aga     RLFCADKKEQSGKSGNIPAGTTVDVGITHPTEFDFYLCSHQGIQGTSRPSHYHVLWDDNHFESDELQCLT
Ago1cpi     ......................................................................
Ago1-1aae   ......................................................................
Ago1-2aae   ......................................................................
Ago1dme     .....E.......................................................D........
```

```
Ago1aga    YQLCHTYVRCTRSVSIPAPAYYAHLVAFRARYHLVEKEHDSGEGSHQSGCSEDRTPGAMARAITVHADTK
Ago1cpi    ......................................................................
Ago1-1aae  ......................................................................
Ago1-2aae  ......................................................................
Ago1dme    ......................................................................

Ago1aga    KVMYFA
Ago1cpi    ......
Ago1-1aae  ......
Ago1-2aae  ......
Ago1dme    ......
```

1C. Ago2 protein alignment

```
Ago2-1Acpi  ------------------------MESAKP-------------------KGNYRGKKK------------
Ago2-1Bcpi  ------------------------......-------------------.........------------
Ago2-2Bcpi  --------MGNSMEVVATETAPEVE.E..AKVVAVARRVNSSSNSPNMV.V.S.D.E.GSTSKQKQQQQQ
Ago2aae     -------MILNARYLYIPCRQKQQQQQQ.QQQQQHQQKQQSQQQQQQQQQQQQ.S.EQGS------QQQQ
Ago2aga     ----------------------------------------------------------------------
Ago2Bdme    MGKKDKNKKGGQDSAAAPQPQQQQKQQQQRQQQPQQLQQPQQLQQPQQLQQPQQQQQQQPHQQQQQSSRQ

Ago2-1Acpi  -PQSGD----------------------------------------------------------------
Ago2-1Bcpi  -.....----------------------------------------------------------------
Ago2-2Bcpi  Q..AQP----------------------------------------------------------------
Ago2aae     R..QQA----------------------------------------------------------------
Ago2aga     ----------------------------------------------------------------------
Ago2Bdme    Q.STSSGGSRASGFQQGGQQQKSQDAEGWTAQKKQGKQQVQGWTKQGQQGGHQQGRQGQDGGYQQRPPGQ

Ago2-1Acpi  ----------------------------------------------------------------------
Ago2-1Bcpi  ----------------------------------------------------------------------
Ago2-2Bcpi  ----------------------------------------------------------------------
Ago2aae     ----------------------------------------------------------------------
Ago2aga     ----------------------------------------------------------------------
Ago2Bdme    QQGGHQQGRQGQEGGYQQRPPGQQQGGHQQGRQGQEGGYQQRPSGQQQGGHQQGRQGQEGGYQQRPPGQQ

Ago2-1Acpi  --------------------------------------------------QDVPEGTS------------
Ago2-1Bcpi  --------------------------------------------------........------------
```

```
Ago2-2Bcpi  --------------------------------------------------PSE.A.G.QQYGDFLQKMQQ
Ago2aae     --------------------------------------------------.QQQPSQQ------------
Ago2aga     ----------------------------------------------------------------------
Ago2Bdme    QGGHQQGRQGQEGGYQQRPSGQQQGGHQQGRQGQEGGYQQRPPGQQQGGH.QGRQ.QEGGYQQRPPGQQQ

Ago2-1Acpi  ------------GQTAGPPQGQQQKQQKSGQGGVGKYKQKQLLKQQQQQDLQQQTGE-------------
Ago2-1Bcpi  ------------.............................................GTQILLPTTSNAP
Ago2-2Bcpi  QQQQVAQPAPAPA.AQAE.PK.A.G..QK.G..G.......QQ...E..E.EK.LAAQRPQQPPAAASAA
Ago2aae     ------------Q.QSQKQ.HP..Q..QRP.KQQQQFQ.D.RPQ.....LQK..QQQGQSWRPQSHDPSP
Ago2aga     --------------------------------------MRIAKEKIRRT..RP-----------------
Ago2Bdme    GGHEQGRQGQEG.YQQR.SGQ..GGH.QGR..QE.G.Q.RPSGQ..GGHQQGR.GQ.GGYQQRPSGQQQG

Ago2-1Acpi  --------------------PIEKSLAKVKLD------------FVRPKN------YGVAGTPVKLEVNY
Ago2-1Bcpi  TP---------------PSQ............------------......------..............
Ago2-2Bcpi  QSRSTASPEGSLSPTHGAIQRV.ED.SGM.I.KG-SGKSALRQVLI..GA------H.RR.KVT......
Ago2aae     ASG--SHSHSSSPSHAAALERV.EDFS.I.I.KQKIHSSALLPVLM..NA------H.TR.RAI.V....
Ago2aga     -------------------------------------------VL..RGA------H.KR.E..SV.A.F
Ago2Bdme    GHQQGRQGQEGGYQQRPPGQQPNQTQSQGQYQSRGPPQQQQAAPLPL.PQPAGSIKR.TI.K.GQVGI..

Ago2-1Acpi  LALNLDKLPAKAYHYDVDIQPAASRKWQRACFSGFRAEALPNRLIAYDGHKNAYTMQPMDQMD-KVGVAV
Ago2-1Bcpi  ...............................................................-......
Ago2-2Bcpi  IP.M.E.MVST..........M........A.KR.EV...AGQP..F..N.....AKKLKLDHY.KE.VA
Ago2aae     IQ.L.ER.IPT..................LA..E.TKQMF..HGF.F.......AARRLQADVYEQE.K.
Ago2aga     FR.L....KGT......A.E.ERPK.FY.PV.AQ.CR.NY.GAML.F..Q.....TRKLSDKKA..VFQP
Ago2Bdme    .D.D.S.M.SV......K.M.ERPK.FY.QA.EQ..VDQ.GGAVL....KASC.SVDKLPLNSQNPE.T.

Ago2-1Acpi  SL-DNRERRFTVSVKLA--NVVDLRSLKG--------------------------------------SCY
Ago2-1Bcpi  ..-..............--..........GNEH------NQAPAKQCLEVVFGTASDRDPRLIRFKR...
Ago2-2Bcpi  RE-...D.K..ITM.E.--A.FRK-------------------------------------------.I.
Ago2aae     RD-EG.....K.AM.E.--A.L.MTC..TYMNNGS-TLDKPMSAIQCLDIVLRTAYENNPRFIKFKK.I.
Ago2aga     DD-GGKQ.EY..Q..E.--AQL..GV..TYMKSNEETMAKPMSAIQCLDVVLRSAYENNPNFVR--RAV.
Ago2Bdme    TDRNG.TL.Y.IEI.ETGDSTI..K..TTYMNDR--IFDKPMRAMQCVEVVLASPCHNKAIRVGRSFFKM

Ago2-1Acpi  FAPSKRIDVGRNHELWYGLHQSLILGSKLFLNIDVAHKAFPSGVPVLDVVGDLARRRWNDSPNVPERIDD
Ago2-1Bcpi  ......................................................................
Ago2-2Bcpi  VV.T.PE.I.A........F..AL....P......S.....R.G......AS.N.N------SL.TTLAG
Ago2aae     VK.DRPD.I.S........F..AL..ARP......S.....T.G...RILV.MN.G------Q..D.VT.
Ago2aga     AV.RQS..I..G....F..F..AL...RPY..V..S.....M.A...K.I..FN.G-------QVDQVSG
Ago2Bdme    SD.NN.HELDDGY.ALV..Y.AFM..DRP...V.IS..S..ISM.MIEYLERFSLK----AKINNTTNL.

Ago2-1Acpi  TLAFKLHNFLKGLEVSYTGPSS---VKKVFKYNSL-RGPASSQLFKREDGTKMTVAAYFT-QQGYRLRHP
```

```
Ago2-1Bcpi  ......................---..........-........................-.........
Ago2-2Bcpi  W..QQV.DY...M..V....NG---.G.T......-K....T.K..L...SES......Q-K..V...F.
Ago2aae     WMSRD..D....M.L.....DG---.S.L.....I-KS..NQ.K..L.N..E..IDQ..R-SKNKQ..Y.
Ago2aga     WVQQE..S....MD.V..N.TTR--MA.RMRC.G.-.E...Q.M..L....RLS..D..ARKLNF...Y.
Ago2Bdme    YSRRF.EP..R.IN.V..P.Q.FQSAPR.YRV.G.S.A....ET.EHD-.K.V.I.S..H-SRN.P.KF.

Ago2-1Acpi  ELPVMHVGSIVRNIMLPMELCQILPGQALNKKHPDECTAQIIKRAATDAPTRKRKIMELRDQISYSNCPI
Ago2-1Bcpi  ......................................................................
Ago2-2Bcpi  D........TI....V.....A.P.....I.....Q..QM..R.S...TV...G...DIFN..D.N..KT
Ago2aae     S...L....L...V...I...S.P...........Q..QF..RKS...TA.......D.FN..G.N.A.T
Ago2aga     N...L....T..SVYV.A...DA.P...ARGEKIP.QR.V.R.EGKN..VPSTELSK.P...R.......-
Ago2Bdme    Q.HCLN...SIKS.L..I...S.EE.....R.DGATQV.NM..Y...STNV......N.LQYFQHNLD.T

Ago2-1Acpi  IKEFGIGVGKDFEVIDGRIIAPPLIEYKNRRTVLPEHGQWSADNEGFITS----NQRELRWIILNLDSYD
Ago2-1Bcpi  ..................................................----................
Ago2-2Bcpi  ..D..F...NS....V....G..SVV.R.NV.IT.SR...R...AS..QI----.PQP...R.....DR-
Ago2aae     .....VS..NN..TV....LD..ELS.R.D.R.K.MR.V.R...MN..IPSTEITR...S.T.....GR-
Ago2aga     LLD...T..NE..KVPA...DA.P...ARGEKIP.QR.V.R.EGKN..VPSTELSK.P...R.......-
Ago2Bdme    .SR...RIAN..I.VST.VLS..QV..HSK.FTMVKN.S.RM.GMK.LEP----KPKAHKCAV.YC.PRS

Ago2-1Acpi  ---TRQRDVDSFGNNVFNESRKKGMQLEPFSMQNNYYEPRNTRMNMKQLETELENSLGYFKKQQLDFVIV
Ago2-1Bcpi  ---...................................................................
Ago2-2Bcpi  ---..PAGIQQ..Q.I.QV...H.I........QT.....DL.YAIREVDSIF.D----L...RM.....
Ago2aae     ---..PDAI.E..R.IYQM.L.Q.V..QQ...K..F....DM.FAV.D.DNIFDE----L..RKI.L.F.
Ago2aga     ---.NEAT.KK..EMLQSQAMRCNV.M...D.AKT.VLV.DM.NCLRDIG.L.Q.----I.REEPAVT..
Ago2Bdme    GRKMNYTQLND...LIISQGKAVNIS.DSDVTYRPFTDDE------RS.D.IFAD----L.RS.H.LA..

Ago2-1Acpi  VIPGIG---DHYSRLKQKAELVL----------------------------YSQINGKTNGTNHVVQSPD
Ago2-1Bcpi  ......---.............VG---VLTSCVKGNTVKNTRSPLTVVNNILLK................
Ago2-2Bcpi  ..S.M.---.Q..KV..R....TG---LLTQCIKGDTVFKKAGDMSTINNIWLK..A........LK-.E
Ago2aae     ...SP.RDG.V.AKV......CVG---LLTQCIKSFTLDKKRGDMSTISNIWLK..A....S...LA-KN
Ago2aga     .L.SR.---.A.AKV......ASERIGLLTQCVKGMTVAKKGTDMSTLNNIMLK..A.......CIS-QV
Ago2Bdme    I..QFR---IS.DTI......QHG---ILTQCIKQFTVERKCNN-QTIGNILLK..S.L..I..KIK-D.

Ago2-1Acpi  PKIPLIKKRIMFVGADVTHPSPEQSTIPSVVGVVASFDRNGFRYKPHFQLQDPKKEMIHGLEAIMQAMLN
Ago2-1Bcpi  ......................................................................
Ago2-2Bcpi  S.P...R..V.Y............TN.......A..Y.LE....NCCYR..N..D...RD..N.IKKQ.L
Ago2aae     F.P.IAR.TV.Y............TN.......A..Y.LE....NCCYR..G..D...RD.QN.VIKQ.R
Ago2aga     AVP..GRGKV.YI.......LS.--NE......A.LY.LT....NCSVR..GARD...RD..N.V.RQ.L
Ago2Bdme    .RL.MM.N-T.YI.........D.RE.......A..H.PY.AS.NMQYR..RGAL.E.EDMFS.TLEH.R
```

```
Ago2-1Acpi  NYKNKNNQQLPEMILYYRDGVSDGQFSQVLDIELNAINRAVAAMNPPSKINVTFVVVQKRHHTRFFPGP-
Ago2-1Bcpi  .....................................................................-
Ago2-2Bcpi  QF.TC.G-A..DL.M.......E....EI.T......QS...STS.GV.VA...I.......A....TRG
Ago2aae     QF.QT.Q-S...L.M.......E...QE..T...R.MQA.A.SVQQGY.P.I..I.......A....TA-
Ago2aga     L..QY.G-A...R.M...........AEI.T...Q.LHA.I.RVE.GY.PA...I............Q.-
Ago2Bdme    V..EYR.-AY.DH.I...........PKIKNE..RC.KQ.CDKVG--C.PKICC.I.V.........SG-

Ago2-1Acpi  KCPKEGRNQNVPPGTIVDRYITTPKHFQFFLTSHRAVEGVAKPSKYTVLHDDEQWDPDRLQAITYALCHM
Ago2-1Bcpi  ......................................................................
Ago2-2Bcpi  TIEV......L....V..KH..A.NQY....I..Q..Q.....T..C..Y..VNS...E..SV.......
Ago2aae     N..T....N..Q..........A.NQY....V..A..Q.....T..C..Y...NCN..Q...L..Y....
Ago2aga     G..T..K.G.........SE....DRYE.Y.V..A..Q.....T..V..Y..SNCH..S...L..N...L
Ago2Bdme    DVTTSNKFN..D...V...T.VH.NEM...MV..Q.IQ.T...TR.N.IENTGNL.I.L..QL..N....

Ago2-1Acpi  YARCNRSVSYPAPTYYAHWVAARGKVYIQG-YWNRVTSENSPDLVRKRRRCQHCGQKHREPMPTARKKGP
Ago2-1Bcpi  ..............................RTL.MAELDRENS.L.I.----------------PEII.E
Ago2-2Bcpi  F.....A...........LA.F..R...KDRRL.MNDLAGEYRKMQIK----------------PEIIDG
Ago2aae     FT....A...........LA.Y..R...KDRPL.MNNLTKEYERMQI.----------------TEIQDG
Ago2aga     F.....A...........LA.Y..R...KE----------------------------------------
Ago2Bdme    FP...........A.L..L.....R..LT.-TNRFLDLKKEYAKRTIV----------------PEFMKK

Ago2-1Acpi  GSSDQI
Ago2-1Bcpi  R.MFF.
Ago2-2Bcpi  HPMFFV
Ago2aae     HPMFFV
Ago2aga     ------
Ago2Bdme    NPMYFV
```

1D. Argonaute 3 protein alignment

```
Ago3cpi   MSSRLNLVRTLLSQSSSS---EDRGSGGTSAEAS---ADTGFQT--RTPETTPNSQRKIIGRGQPAGSVP
Ago3aae   .........AF....G..GDAAE.TKAPS.G..PSEGV.S....--....S..TT...G.....LTSDAG
Ago3aga   ....KDFF.SIIES...IG-----..NND..N.TSQNL.S.YR.GVT.SGEE.AAPLPVQ...R-----A
Ago3dme   ..G.G..LSLFNKNAGNMG---------K.ISSKDHEI.S.LDFN----NSESSGE.-------------

Ago3cpi   -TIGTSGLDSSESDNQASVVSSNVGPTLSGRGRAQFIQGLLKQPLESGRSIPTVADDSASTISSAPAVSI
Ago3aae   RAT....A......DKQ.SIG.-AM.S...........A.IR..F.PAP.V--.S...S.MV.AR--..Q
Ago3aga   WALSSL.VTMAPPADA.APLVT-PMEN.VMG..GR.L.K..S.AAPTAATVEPP..TTVPIS.AT-----
```

```
Ago3dme  -LLSSHNIETDLITTLQH.NI.------V.....RL.DT.KTDDHT.NQF.TSESKENITKKTKG-----

Ago3cpi  LGAGRGRFMQQLMNTRADEPSVSDESK---TQLTEQMSQITIAETVQTVEREPVKRMGTKGTPVQLMTNY
Ago3aae  IAC.....I...L..A..AE.IETQ.NGKHDE.S.AV..V......E...KA..IKT.SS....R.....
Ago3aga  --S.A.----TASIKDS.AR..ASRQA---SDF.KPEDSFVLR.CKEQG.--..I.I.KE.N.LH..G.F
Ago3dme  ----------------PESEAIAS.NG----------------------LFF.DLIY.S..SS.NIYC..

Ago3cpi  IRIACDPDRGLYEYEVRFNPEVDSKVARSKYINQHREVIGNAKTFDGVKLFLPKKLPNVETIL--QSENE
Ago3aae  ..L.......I.......H.L....AI.AR..A..KD.L..................QA.LT.--..K.P
Ago3aga  MELK.E....IFL.T.D.S.PI.A.RT.QRC.DS.KD.F.T.Y....HI.L...A.EQERFK.--T.QHP
Ago3dme  LKLTT.ESK.VFN.....F.PI..VHL.I..L.D.KDKL.GT.....NT.Y..IL...KM.VF--I.K--

Ago3cpi  VDGHTVTIRLMYKRKQRMSENIQFYNILFQRIMKVLKMIEMGRKNFDPSAPKLIPQHRLEIWPGYVSAVD
Ago3aae  ....E...KII...........................V...............................
Ago3aga  TE.TP..LSVVFRVQ.Q..Q.VA...K..R...YM.QLS.....H...TQARI....K......F.T..N
Ago3dme  AEDVELQ..IL..K.EE.RNCT.L.....D.V....NYVKFD..Q....R..I..LAK..V.....T...

Ago3cpi  EYEGGLMLNLDVSHRVLLQTTVLDHIRTIAKSNPSDWKNLVTKSLLGAVILTRYN-NKTYRIDDIIFDQN
Ago3aae  ............................L.RA..Q.Y..MA..............-.........L....
Ago3aga  .F..........T....M....YT..KML.QARGGQFREN.L.......V.....-K.......VC..T.
Ago3dme  ..K.....CC.....I.C.K...EMLVDLYQQ.VEHYQESAR.M.V.NIV.....-.R..K.N..C....

Ago3cpi  PTMTFMSSNRQQQISYVEYYKQQYNITIHDLKQPLLIHRKERRIAGQDKPQELMMCLIPEISYLTGLTDE
Ago3aae  .....EANG--.P...LQ........D..........N.....VS.....M.MI.......C........
Ago3aga  .LS..RYGE--RD........E..GLE.K.HQ....LN.S...VV.KTE.E.MIV..V...CF.......
Ago3dme  ..CQ.EIKT--GCT........YH..N.K.VN...IYSI.KS.GIPAER-EN.QF.....LC.....R..

Ago3cpi  MRNDFKVMRDIAAFTRVSPNQRLNSMRQFCYNVNQNKEAREILEVWGLKLDMEPLVMKGRAFEEEKVIFG
Ago3aae  ..S...........................R..E..............T...NA.M.E..C.K..Q.N.S
Ago3aga  ....H.......TY..IT.....VA.....E...K.EA..KL.AS...E.MIT.K..M..QLPP.NITS.
Ago3dme  V.S.N.L..E..T.........QMALNK.YE..SNTPA.Q...NS...S.TNNSNKIS..QMDI.QIY.S

Ago3cpi  NAQVG----VGKAGDFNRAVTSSNMLSAIPIRKWLLVHTNKDMKIAKSFMDCVQRSCRPMGIEVDTPAIE
Ago3aae  GVN..----...S.........N.V.Q.VN..N......A..TR.........E........QIGP....
Ago3aga  GGV..S---A.PNV.YT.Q..NNP..EIVH..Q.M.LY.QR.EQH.SV.R...K.T.KLL.L..AQ.QV.
Ago3dme  KIS.S----A.RSAE.SKHAVTNE..KVVHLS..III.LRNYRQA.T.LL.NMKQA.ESL.MNISN.TMI

Ago3cpi  VLPTDKTELYVQLLRTKIRQDTQIVVIICPTSRDDRYAAIKRICCSEIPVPSQVINARTLANEA--KNRA
Ago3aae  ..QA................E.......................................S...--....
Ago3aga  ...Q.A.N....A...R..PG......................L.T.V.I.............K--R..S
Ago3dme  S.DH.RIDA.I.A..RN.TMN..M..C..HNR.........K.......I.......K..Q.DL--.I.S
```

```
Ago3cpi  IVQKILLQMNCKLGGTLWSIKIPFQNVMIAGIDTYHDPKQKSNSVSAFVASLNG----EYTRWYSRACIQ
Ago3aae  .....I..............R...D.............................----D...........
Ago3aga  V.L.V..........S..GVRV.IKRT..C......EA..R....A...G..DA----AF.H.....TV.
Ago3dme  V....V.........S..TV....K....C...S....SNRG...A.....I.S----S.SQ...K.VV.

Ago3cpi  SKKEEFMNGLCASLEKSLRAYE-KLNGVLPQKIIIFRDGVGDGQLRMCSEYEIPQLMEACKLV--EPNYC
Ago3aae  ......I.....M....K..Q-.A.CQ..K.........................Q.S....--..D.N
Ago3aga  ER...IL....V....T.Q..Q-RR.CH..DR........S.E.TNI.LD......QA.....--Q.D.E
Ago3dme  T.R..IV...S..F.IA.KM.R-.R..K..TN...Y...I.....YT.LN.....FEMV.G-------NR

Ago3cpi  PELAFIVVQKRINTRMFRIDGQ----SNLDNPNPGTVLDHTVTRRNHFDYFLVPQSVRQGSVSPTHYIVV
Ago3aae  ..IT.................-----N..E...........I............................
Ago3aga  .KIT........I..L.TMSRGGGDGDGQA.AP......N.....YM..F...S.T.QM.T.T......L
Ago3dme  IKISY..........I.SGS.-----IH.E..L....V.QHI.KS.MY.F...S.L....T.T....V.L

Ago3cpi  HNQPDYSPDVLQRLSYKLCYLYYNWPGSVRVPACCQYAHKMAYLIGQSVKRNPDEVLNDKLFYL
Ago3aae  ...SNH...I.............................................A........
Ago3aga  RDDSKF...I..Q....M..M....T.TI...........L...V......M.A.S........
Ago3dme  RDDCN.G..II.K......F.....A.T..I....MVS.NYHLIFFK.T-MITQDTI..FTRA.
```

1E. PIWI protein alignment

```
DmePIWI   --------MADDQGRGRRR-----------------------PLNEDDS--STSRGSGD------------
DmeAub    MNLPPNPVI.RGR....KP-----------------------NNV.ANRGFAP.L.QKS------------
AgaAgo4   ---------...GRREPPPYGRDRIR----------------GSLGSN.PYEGG..G.--HPSMRGGYPP-
AaePIWI2  ---------...R----QPVRRARAR----------------GYAAVSESHESRQ.R.--QPPVRGSGVA-
AaePIWI1  -----------------------------------------------------------------------
AaePIWI2  ---------.S.R----QSQGRARAR----------------GYTAVNLSHESRE.R.--HAPVRGSGVG-
AaePIWI4  ---------.S.R----YSQGRYRAR----------------GYT.VG.PHEGRGSPS--SRPAYSS----
CpiPIWI2  ---------.S.RE---HD.DRPRAR----------------GYTDS---HEGRGS.SGHSRPAYSSE---
CpiPIWI3  ---------.S.R----QAQGRARAR----------------GYTAVNLSHEGHE.R.--QAPVR------
CpiPIWI1  ---------.S.R----QAQGRARAR----------------GYTAVNLSHEAHE.R.--QAPVRGSGVPV
AgaAgo5   -------------------------------------------PVWPAPCAASPPSPDR------------
AaePIWI5  ---------.CVNKA..HTSGQNWPS---------------LGQQQPQQQFQGWKQPQQ------------
CpiPIWI5  ---------...R.Q..N.-----------------------GRGRARG-FAGAGRG.Q------------
AAePIWI7  ---------..EYRP..G.GGNNQARGNVGGEGSFPPLNGNNGHRSSNGTYRNDGQWKQSNGNGGGSNRDW
AaePIWI6  ---------...NPQE.SSGGRIRARG-------------YVGGSSSRGGYNGGSSR.AWP----------
```

```
CpiPIWI4  --------.S.YPQP.SSSD--------------------TGRIRARG-FVGASRG.RNG----------
CpiPIWI6  --------.S...QQPEFS---------------------GGRIRARG-FVGASRG.RNGNGRPSGG---
BmoPIWI   --------...PGKGRG.SLALLQALKKSQMMDSPSQSESQSPESTPEQSTAPSTIASATPSTSGVSIG-

DmePIWI   ------------------------------GPRVKVFRGSSSGDPR----------------ADPRIEAS
DmeAub    ------------------------------D.SHSEGNQA.G.NGGG---------------G.AQVGP.
AgaAgo4   ---------------------HGGPGGAGG.AGASYQTQERRPTW.N---------------PS.GPSE.
AaePIWI2  ------------------VSGPRPSFQHPGAEGRA.TYHEG.AGRGA---------------VSASTSGG
AaePIWI1  ----------------------------------------------------------------------
AaePIWI2  ------------------ISGPRPTFQHPGAEGRAMTHRDA.AGRG----------------.SSSTSGN
AaePIWI4  -----------------------------RDEGRSREPREHHRE--------------------SSAGGR
CpiPIWI2  ----------------------------GRDRGEGRGSREH.QSRG----------------NGNGGGDR
CpiPIWI3  -----------------------------A.EGRAMVHREH.AGRG----------------.SSSTGNG
CpiPIWI1  S-----------------GQGPRPSWGPGA.EGRAMVHREH.AGRG----------------.SSSTGN-
AgaAgo5   -----------------------------RTRSRPRN.RPPRTI.-------------------.PPPPA
AaePIWI5  -------------------KPPQQPQQPAPVQQQPQQQPQQQPQHQPQKQQ----------PQQQPPPQT
CpiPIWI5  -------------------GQGQG-------HYQAEHAPVQQHRQEPSTSR----------PQQQ-----
AAePIWI7  KESGGGYANRKDANEGVRNGNGRQFHNGGRNHNQSGGDRVNN.RQFVDHQRNSNERYGEDRRQKHHDGNP
AaePIWI6  -------------------GSDNGGGSYRGDH.Q.PYDRPEQSS.SIAVKEEMS--------.LESDIKK
CpiPIWI4  -------------------GGGYQHGDQ-R.QVGSFQHRQQYFQ----GRAPY---------QQA.GGPA
CpiPIWI6  -------------------GGGWPGSEQHR.QIGSFQHRQQQFQNYGAGRSPG---------PQSPYGGG
BmoPIWI   ------------------GRGRAAALMLAKMQQKPGSTTPAIFV.PSSTSAPTAGTGRGFKLLQSLQASQ

DmePIWI   RERRALEEAPRR---------------------EGGP-TERKPW------GDQYDYLNTRPAELV-SKKG
DmeAub    I.KSS.SAVQMHKS-------------------..D.RGSVRGR------RLIT.LVYS..PGMT-....
AgaAgo4   .DAVPSTS.APQPSSSG-----------------L.NIGRPLHRAAGPRRRPLA.T.R..GPDAP-..H.
AaePIWI2  GNGNGNGGDGNGNGAAA-----------------VASRGAMRGR------RPVG.T.R..ALDAP-..H.
AaePIWI1  ----------------------------------------------------------------------
AaePIWI2  GNGNGS------SGSAA-----------------.TSRGAMRGR------RGVA.T.R..ALDAP-..H.
AaePIWI4  ERS-GNGNGGD.PGG---------------------ERG..RQRSR----RGVG.T.R..ALDAP-..H.
CpiPIWI2  DRE.GGGGGG..EGGGG-----------------RDERGG.RGR-R----RTYG.T.R..G.DAP-..H.
CpiPIWI3  NGDNGNGSSAQSSSAAG-----------------A.SRGAMRGR------RTIG.T.R..ALDAP-..Q.
CpiPIWI1  -GDNGNGSSGQSSAATG-----------------A.SRGAMRGR------RTIG.T.R..ALDAP-..Q.
AgaAgo5   ATG.RVWR..GAGDGP-------------------SGRGAMRGS------RYVPEVVV..LPNAP-..H.
AaePIWI5  ESTG.VQRRGATGGEA-------------------PGRGGVRNQ------RVLQ.IVRS..LDSKVV.Q.
CpiPIWI5  .GGGGPA..GAAG-----------------------GRGAMRGN------RFVP.V.R...VNSTV..H.
AAePIWI7  SSSNPIV.K.VHSTETP-------------QTSS.VARGGMRGN------RAIAEIVR...IDTTIT.Q.
AaePIWI6  ERTYDRG.RGG.GGSR-------------------.GRGGLRGN------RYLPEIVQ..-NDMSVV.Q.
CpiPIWI4  EAASPVG.VSILVT-------------AQRGSGSVRGRGGMRGN------RAIPEVVV..MPGELA..Q.
CpiPIWI6  EAG-PV..VSALVRQSSPSVHRSSERAFQRGGGAA.GRGGMRGN------RYLPEIVI..KPGDAQ..Q.
BmoPIWI   KASSQVASSQVTSSAQS-------------DIKDLTEKMSETSVSAQTSSVAKNK.FREVKDTPPVV...
```

```
DmePIWI   TDGVPVMLQTNFFRLKTKPEWRIVHYHVEFEPSIENPRVRMGVLSNHANLLGSGYLFDGLQLFTTRKFEQ
DmeAub    VV.THITV.A.Y.KVLKR.N.T.YQ.R.D.T.DV.AT.L.RSF.YE.KGI..-..I...TNM.CINQ.KA
AgaAgo4   .T.Q.LQ..S.Y.K.LKRVD.TLY..Q..MV.PCAS..LMQSLVNE.KK...-.FV...V....AC.LSN
AaePIWI2  .A.Q.LQ..A.Y.K.LKHI..TLYQ.R.D.A.ACDSI.LMQ.LI.Q.KKTF.-......T...MVN.LRS
AaePIWI1  ----------------------------------------------------------------------
AaePIWI2  .T.Q.LQ.HA.Y...RKHI..TLYQ.R.D.A.TCDSI.LMQ.LI.Q.KKIF.-......T...MVN.LKS
AaePIWI4  .T.QALQ.NS.Y.K.LKHI..TLYQ.RLD.S.QCASM.LMQ.LVNE.KKIF.-.F....T...MVN.LRS
CpiPIWI2  .T.QALQ.HS.Y.K.LKHI..TLYQ.R.D.S.QCASS.LMQ.LINE.KKIF.-.F....T...MVN.LRS
CpiPIWI3  .M.Q.LQ.TS.Y.K.LRHI..TLHQ.R.D.A.QCASA.LMQ.LIKE.KKTF.-.F....T...MVN.LRS
CpiPIWI1  .M.Q.LQ.TS.Y.K.LRHI..TLHQ.R.D.A.QCASA.LMQ.LIKE.KKTF.-.F....T...MVN.LRS
AgaAgo5   .L.TKLL.K..Y...TR.EDSV.FQ.RID.D.VV.DQ..VHAL.RTQGP.F.-P.I...TM..LYN.LR.
AaePIWI5  KT.T.I..K..Y..VQRRDDEA.FQ.R.D.N..V.SIKLMHSIIQ.LKGTI.-..I...T....RH.LRS
CpiPIWI5  .K.KQIL....Y..VGR.DNES.FQ.R.D.N.VV.SS.LMSALVF.LKPVI.-......T....RHRLRS
AAePIWI7  .S.RQI.....Y..VAR.DDEC.FQ.R.D.N.PV.SS.LLRSLVYGLKPTI.-..I...T....RH.LRS
AaePIWI6  NS.R.I.....Y..VLK.EDE..F..R.D.S..V.AN.KMRALIFQIKPSI.-..V...T....RT.L.K
CpiPIWI4  .S.K..V....Y.HV.RLEDL.LFQ.Q.D.N.PV.TRK.SGAIIA.LKPQI.-.N..R.A..YSRN.LCD
CpiPIWI6  KL.T..I....Y.KVIRQGDE.LYL.R.D.T.PV.TRKAQNSIMFTLKPQI.-......T....RN.LCD
BmoPIWI   ET...IEVTC.YIY.NF.EN-IVFE.E.K...DQDYKHL.FKL.NE.IEHFK-EKT...TT.YVPHELPD

DmePIWI   EIT-----VLSGKSKLDIEYKISIKFVGFISCAEPRFLQVLNLILRRSMKGLNLELVGRNLFDPRAKIEI
DmeAub    VQDSPYVLE.VT..RAGENIE.K..A..SVQSTDAEQF.........A.E..D.K..S.YYY..Q...NL
AgaAgo4   DELV----LH.QHDRTGDK.ELH.QR.AVVDMTDETGI.........A.N....Q......Y.AA...P.
AaePIWI2  .QLS----LECRHERTGEV.QVK.VHT.TVDMTNETGI.........A.G....Q......Y.AA...A.
AaePIWI1  ------------------------------MTNETGI.........A.G....Q......Y.AA...A.
AaePIWI2  .QLS----LECRHERTGEV.Q.K.AHT.TVDMTNETGI.........A.G....Q........AA...A.
AaePIWI4  DQLT----LQ.RHERTGDV.QLR.VHT.SVDMTNEAGI.........A.A....Q......Y.AA...A.
CpiPIWI2  DQLT----LQ.RHERTGDV.QLR.IHT.SVDMTNETGI.........A.A....Q........AA...A.
CpiPIWI3  .QLT----LT.KHERTGDV.Q.K.VHT.TVDMTNETGI.........A.G....Q........AA...A.
CpiPIWI1  .QLT----LT.KHERTGDV.Q.K.VHT.TVDMTNETGI.........A.G....Q........AA...A.
AgaAgo5   DEVE----LQVKDPRT.LL..LR.RR..TVDMTSEKAFLI...MH.QA.GS.K.LPIN.YY...L...S.
AaePIWI5  .EVE----ITTKE.TTGND.IVKLRK..VVDGTNEMAFVIF...N..A.A..K.QMI...Y...V..VA.
CpiPIWI5  DEVE----YPTKDPISGQS.I.KLRK..T.DGTTEMAFMIF...N..A.N..K.T.I...Y..GQ...S.
AAePIWI7  DEVE----ITTKD.TS.Q..I.KLRR..V.DGTNETA.MIY...N.KA.G....Q.I...F...A..VTV
AaePIWI6  DPME----FTTRDKMTEEDHI.TLRY..DVTGTDEKAFV.F...N.HA.A..K.Q.I..SF...E..VS.
CpiPIWI4  KEIE----YNTVYKATNEH..VKLRR..EVDGTNEVAF..Y...N.MA.E..K.Q.I....Y..A.M.RL
CpiPIWI6  .EIQ----YNTKYNATNDD.TVKLRR..VVEGTNEAAF..Y..MN.KA.G..K.Q.I...F...D..VNL
BmoPIWI   AVRN------LVSTNPYDQS.VNVSIIFRRTRRLSEMIHIY.VMFKCI..D.K.IRF..QHYNEH.A.Q.

DmePIWI   REFKMELWPGYETSIRQHEKDILLGTEITHKVMRTETIYDIMRRCSHNP-ARHQDEVRVNVLDLIVLTDY
DmeAub    EN.R.Q.....Q.......N....CS..C........L.N.LSDAIRDS-DDY.STFKRA.MGMVI....
```

```
AgaAgo4   ..YHI......I.......NEV.VCC..A..T..MQ.C...L.E.QRHD-RNFK.AF.RA.IGCV...G.
AaePIWI2  .D.HI......V.......Q...VCC..A..T..MQ.C..VL.D.RT.D-RNYK.AFTRS..GVV...G.
AaePIWI1  .D.HI......V.......Q...VCC..A..T..MQ.C..VL.D.RN.D-RNYK.AFTRS..GVV...G.
AaePIWI2  .DYQI......V.......Q...VCC..A..T..MQ.C...L.E.QRHD-RSYK.AFTRS..GVV...G.
AaePIWI4  ..YQI......I.......Q...VCC..A..T..MQ.C...L.D.QKHD-RNYK.SFKRA..GVV...G.
CpiPIWI2  ..YQI......I.......R...VCC..A..T..MQ.C...L.D.QKHD-RNYK.SFKRA..GVV...G.
CpiPIWI3  ..YQI......V.......Q...VCC..A..T..MQ.C...L.E.QRHD-RNYM.SFKRA..GVV.....
CpiPIWI1  ..YQI......V.......Q...VCC..A..T..MQ.C...L.E.QRHD-RNYM.SFKRA..GVV.....
AgaAgo5   PQYGL..F...V.........V..CV....R....D.V.KTLQNMQRQ.-GAFRENFVKM.VGSQ.MCT.
AaePIWI5  SQYGI..Y...V.......Q.V.MCA....R....D.C.TMFKQ.ANQG-GNWR.NYKRAI.GSV.MAT.
CpiPIWI5  QQYGID.Y...V.......Q.V.MCA.L..R....D.C.MMFKT.LNQG-.NWR.NYKRM..GTV.MAT.
AAePIWI7  SQYGI..Y...V.......N.V.MCA.L..R....D.C.TL.KQ.MNHG-GNWK.NFKRMI.GSV.MAT.
AaePIWI6  .QYGID.Y...L.......Q.V.MCA.L..R....D.C.SLFEH.MNQR-GNFK.NYKRM..GT..MST.
CpiPIWI4  Q.H.LD.Y...L.......Q.V.MCV.L..R......CL.LLLA.VNFR-GNF..NF.RQ.IGT..M.T.
CpiPIWI6  AQY.ID.Y...I.......T.V.MCA.L.......D.CF.LFEK.M.QR-GNF..NYKREIIGST.M.T.
BmoPIWI   PQH.L.V....V.AVDEY.GGLM.TLDS..R.L..Q.VLSLIKEVVQTEG.NWKRKMTDILIGAS.M.T.

DmePIWI   NN-RTYRINDVDFGQTPKSTFSCKGRD----ISFVEYYLTKYNIRIRDHNQPLLISKNRDKALKTNA--S
DmeAub    ..-K....D....QS..LCK.KTNDGE----..Y.D..KKR...I...LK...VM.RPT..NIRGGN--D
AgaAgo4   ..-K..T.H..T.ET..E...DT.NGK----...LD..KN...L....SY..M.L.RAKK.DTRSGD--.
AaePIWI2  ..-K..T.H..S.ET..S...ET.NGK----.......QQ........P...M.L.RAKKRD.RAGG--N
AaePIWI1  ..-K..T.H..S.ET..S...ET.NGK----.......SRVH-.....RQRSKK.ETTIKHQVNFQFP-I
AaePIWI2  ..-K..T.H..S.DT..S...ET.NGK----...ID..QQ........P...M.L.RAKKRD.RAGG--N
AaePIWI4  ..-K..T.H..T.ET..E...DT.AGK----T..I...KQ........PH..M.L.RAKKRD.RAGG--.
CpiPIWI2  ..-K..T....T.DT..E...DT.AGK----T......KQ........PH..M.L.RAKKRD.RAGG--.
CpiPIWI3  ..-K..T....S.DTN.Q...ET.NGK----T......QQR.......AQ..M.L.RAKKRD.RAGG--C
CpiPIWI1  ..-K..T....S.DTN.Q...ET.NGK----T......QQR.......AQ..M.L.RAKKRD.RAGG--C
AgaAgo5   .Q-KS.KVT....NT..S.S.TSREGEN---.T.M...RKA...T...PK..M.V.TPSQRM.RSG.--.
AaePIWI5  GRNN..T.A..E.NTS.E.A.DAA.TR----VT.MQ..KDR...T...PR..M.V.RAKQRDIRAGK--.
CpiPIWI5  GKNN..T....E.NT..E.S.ETSNGK----.T.LQ..KER...I...PR..M.V.RAKLRDVRAGK--P
AAePIWI7  GSN...T....EYST.AE.S.QTSNGQ----....Q.FRER...I...PR..M.V.RSKS.DIRAGL--P
AaePIWI6  GKNN..TVS..E.DVN.E.S.ET.KGP----.T.MQ.FKE....T...PR..M.V.RSKARDIRAGQ--P
CpiPIWI4  GSNK..T......SI..E...ET.TGP----...LQ..RDR..VT.S.RR..M...RAKARDIRAGM--P
CpiPIWI6  GSNK..VV.....SMS.Q...ET.NGP----.T.LQ.FRDR..VT.K.KG..M...RSKARDIRAGM--P
BmoPIWI   .K-KLF.VDTI.DKMS.R...EKTEKGETVQ...ID..KKN.G.E.M.WD......RDTKRMPGSDTPTD

DmePIWI   ELVVLIPELCRVTGLNAEMRSNFQLMRAMSSYTRMNPKQRTDRLRAFNHRLQNTPESVKVLRDWNMELDK
DmeAub    QAIMI....A.A..MTDA..AD.RTL....EH..L..DR.IE...M..K..KSCKQ..ET.KS..I...S
AgaAgo4   ..MA.V....QM...TDS...D.KM....ADH..L..DR.IE..ET..R...TS...QE.FKV.Q....R
AaePIWI2  ..MA.V....QM...TDQ..ND.RM.....D...L..DR.IE..ET..R...T....ME.FKV.Q.....
AaePIWI1  DSAEIY.CFL.NFLVIHSSK.RYSM.....D...L..DR.IE..ET..R...T....ME.FKV.Q.....
```

```
AaePIWI2  ..MA.V....QM...TEQ...D.RM.....D...L..DR.IE..ET..R...T....ME.FKV.Q.....
AaePIWI4  ..MA.V....QM...TDQ...D.RM....ADH..L..DR.IE..ET..R...TS...ME.FKT.Q....R
CpiPIWI2  ..MA.V....QM...TDQ...D.RM....ADH..L..DR.IE..ET..K...TS...ME.FKT.Q....R
CpiPIWI3  ..MA.V.........TDQ...D.RM.K...DH..L..DR.IE..NT..N...TC...AD.FKI.Q....R
CpiPIWI1  ..MA.V.........TDQ...D.RM.K...DH..L..DR.IE..NT..N...TC...AD.FKI.Q....R
AgaAgo5   GPIL.V.....L..ITDD..RD.N...SIADQ..IGADK.IE..QR..E...E..A.RE.FTF.KT...R
AaePIWI5  ..IY.V...V.A..ITD...K..N...TLADH..LT.DK.IQ..EH..L...QSK..SEIFQF.KT...R
CpiPIWI5  ..IY.....V.A..ITD...R..N...TLAD...LT.DK.IQ..ET..Q...SCE..AD.F.F.QT...R
AAePIWI7  ..IY.V...V.A..ITD...R..N...TLADH..LT.DK.IE..EV..R...DSK..TEIFQF.KT...R
AaePIWI6  ..IY.....S.I..ITDD..RD.H....IADH..L..DK.IQ..ET..R...QSKD.SD.FKF.KT...R
CpiPIWI4  ..II.V...S.I...SD.N.RD.R...DLAGH..LS.DR.IV..EQI.D..KKCKD.ADIF.F.KT...R
CpiPIWI6  ..II.V...S.I...SD...RD.....GLAEH..LA.DR.IA..ET..Q...YCKD.ADIF.F.KT...R
BmoPIWI   FMIC......QL...TDDQ....R..KDVAT...IT.N..HAAFKKYIESVMKNETAKSR.AG.GLSIAP

DmePIWI   NVTEVQGRIIGQQNIVFH----NGKVPAGEN-ADWQRHFRDQRMLTTPSDGLDRWAVIAPQRNSHELRTL
DmeAub    ALV.IPA.VLPPEK.L.G----.Q.IFVCDAR...TNE..TCS.FKN--VHIN..Y..T.S..LR.TQEF
AgaAgo4   RLV.LP..LLP.EM.F.S--TTSNG.Q....-...TA....NP.FA.--VR.S..YL.V.S.CQR.AGDF
AaePIWI2  RLV.LP..LLP.EM.Q.S--TTSTG....D.-...TM...NNP..S.--VR..H.YI.V.S.AQR.ANEF
AaePIWI1  RLV.LP..LLP.EM.Q.S--TTSTG....D.-...TM...NNP..S.--VR..H.YI.V.S.AQR.ANEF
AaePIWI2  RLV.LP..LLP.ET.F.S--TTAAG....D.-...TM...NNP.FA.--IR..H.F.VV.N.VQR.ANDF
AaePIWI4  RLIDLP..LLP.EM.F.S--TTANG.Q...Q-...TA...NNP.FA.--VR.T..YL.V.N.A.R.ANDF
CpiPIWI2  RLV..P..LLP.EM.F.S--TTANG.Q...Q-...TA...NNP.FA.--VR.N..YL.V.N.ATR.ANDF
CpiPIWI3  RLV.LP..MLP.EL.F.S--PSA.GF.S..Q-...SI...NNP.FS.--VR.NH.YL.V.N.ANR.ASDF
CpiPIWI1  RLV.LP..MLP.EL.F.S--PTA.GF.S..Q-...SI...NNP.FS.--VR.NH.YL.V.N.ANR.ASDF
AgaAgo5   RLV..PA.LLA.ET.M.R--ADG.G....PE-...NQA..NNH.FK.--VP.Q..FIVCERKAEQLT.DF
AaePIWI5  RLV..PA.VLRPEE.F..PSQE.Y..T..DV-....MA..NNP.YIS--IP.VN.YF.V.AGSEKLMVDF
CpiPIWI5  RLV..P..VLAEEE.L.N-ANETN..L..PQ-....MA..NNP.YMS--VP.RN.F.VVSAGDQNLVGSF
AAePIWI7  RLV..PA.VLQPET.F..PEQP.YA.A...M-.E..MA..NNP.YHS--VA.TK.FAVV.KGSERLITDF
AaePIWI6  RLV..PA.VLPPETVF..PEQDQC..L...M-.E..MA..NNP.YL.--VA.TN.Y.VV.GGSERLIVDF
CpiPIWI4  RLV..R..VLPTEM.LL.PVETKARTS..DK-....AA..EKP.YL.--VA.KH..IVV.A.ETRNIGNF
CpiPIWI6  RLV.....VLPPET.F..PESEQY.MS..DT-.E..MA..NNP.FL.--VS.TN..IVI.GN.QKNVNDF
BmoPIWI   ETVNLTA.TLPPETLY.G---D.VR..GKP.-.E.NSEVTKHSVMQA--VDIM..VLLFT..DKQVAMDF

DmePIWI   LDSLYRAASGMGLRIRSPQEFIIYDDRTGTYVRAMDDCVRSDPKLILCLVPNDNAERYSSIKKRGYVDRA
DmeAub    VQMCI.T..S.KMN.CN.IYEE.P...N...SQ.I.NAAAN..QIVMVVMRSP.E.K..C....TC...P
AgaAgo4   .NCMIT..R..RFE.SNCEMVPMP..SP...I.TL.AIINR..QM.M.V.S.SKSD..TA...KCC.E..
AaePIWI2  .GC.MQ..Q..RFDVKRCEFVT.P..SP.....ML.NV.NK..Q..M.V.T.QK.D..TA...KCC....
AaePIWI1  .GC.MQ..Q..RFDVKRCEFVT.P..SP.....ML.NV.NK..Q..M.V.T.QK.D..TA...KCC....
AaePIWI2  .GC.MQ..R..RFE..QCEFVA.P..NP.....ML.NL.NK..Q..M.V.T.QK.D..TA...KCC....
AaePIWI4  .GCMIQ..R..RFE.SNCEVVT.P..NP.....TL.NLLNK..Q.VM.V.T.NK.D..TA...KCC....
CpiPIWI2  .GCMIQ..R..RFE.SNCEIVT.P..NP.....TL.NILNK..Q..M.V.T.NK.D..TA...KCC....
```

```
CpiPIWI3  .GC.IQ..R..RFE.DQ.EMVA.P..NPA....TL.NV.NR..QM.M.V.S.NK.D..TA...KCC....
CpiPIWI1  .GC.IQ..R..RFE.NQ.EMVA.P..NPA....TL.NV.NR..QM.M.V.S.NK.D..TA...KCC....
AgaAgo5   VGAMMQVSN...FKVAQ..ILYLQN.SGAS.TN.INELMNK..Q.VM.I....R.D..KA...KCC...P
AaePIWI5  MQC.KQ..R..RFQ.ED.NRVV.QN.SPAV..ESLNQV.QR..Q.VM...S..K.D..AA...KSC....
CpiPIWI5  MNCIHDV.R..RFE.SK..FEV.QN.SPAV..DCLNRL.QQ..QM.F.V.S..K.D..AA...KTC.E..
AAePIWI7  MQC.RQ..R..RFQ.ED..LIV.PN.SPAV.IDSLNSI.QR..QM.M.V.T..K.D..AA...KCC....
AaePIWI6  MSC.KQ...Q.YFQ.DE.RRVS.PN.SPVV..EQLSQI.QR..Q..M...T..K.D...A...KCC....
CpiPIWI4  .NC..QVSRK.TFM.NQ.HFVE.SN.SPNV..ETLEQLCNR..Q..M.V.TD.K.D..AA...KCC.N..
CpiPIWI6  .SC..Q.SRQ.HFLVEE..FVP.HN.SPVV.METLNQL.QR..Q..M.I.T..KVD..AA...KCC....
BmoPIWI   .ST.K.NCRP..IMVSDAELVPLAN...D...L.LKK.IT.SVQ.VVAICSTKRDD..AA...VCCA.NP

DmePIWI   VPTQVVTLKTTKNRS-----LMSIATKIAIQLNCKLGYTPWMIELPLSGLMTIGFDIAKSTRDRKRAYGA
DmeAub    ..S......VIAP.QQKPTG.......VV..M.A..MGA..QVVI..H....V...VCH.PKNKNKS...
AgaAgo4   I...IMVQ..ITPK.GNVRT...V...VV..M.....GV..KVKI..N........VCHDSK.KSKSF..
AaePIWI2  .....ICQ..ITPKGGNVRT...V...VV..M.....GV..KVKI....M......VCHD.N.KSKS...
AaePIWI1  .....ICQ..ITPKGGNVRT...V...VV..M.....GV..KVKI....M......VCHD.N.KSKS...
AaePIWI2  .....ICQ..ITPKGGNVRT...V...VV..M.....GV..KVKI..N....V...VCHD.N.KSKS...
AaePIWI4  I....MVQ..ITPKGGNVRT...V...VV..M.....GV..KVKI..N........VCHDGK.KSKSF..
CpiPIWI2  I....MVQ..ITPKGGNVRT...V...VV..M.....GV..KVKI..N........VCHDAK.KSKSF..
CpiPIWI3  I....MVQR.ITPKGGNVRT...V...VV..M.....GV..KVKI.......V...VCHD.K.KSKSF..
CpiPIWI1  I....MVQR.ITPKGGNVRT...V...VV..M.....GV..KVKI.......V...VCHD.K.KSKSF..
AgaAgo5   L.C..IKAR.ITPKNQNLRA.LT....VM........GI..IVKN...SV.VV...VCHDAS.KSLS...
AaePIWI5  .A...IKAR.ITPKGGNVRT...V...V.........GI..VLKN..TSV.V....VCHD...KSKSF..
CpiPIWI5  I....IKAR.ITPKGGNVRT...V...V...M.....GI..VLRN..TSV.V....VCHD...KSKS...
AAePIWI7  .A...IKTR.ITPKGGNVRT...V...V...V.....GI..ILKN...SI.V....VCHD...KSKS...
AaePIWI6  .....LKTR.ITPKGGNVRT...V...V...M.....GI..V.KS..ASV.V..Y.VC.DSK..SKG...
CpiPIWI4  I....IKSR.ITP.HGN--MML.V...V....S...GI..VVKVLMESV.CV...VCRD.K.KNIC...
CpiPIWI6  .....IKC..ITPKGGNPRT...V...V.........GI..IVKS..LSV.CL...VCRD.K..NKT...
BmoPIWI   ..S..INAR.LM.TN----KIR..TQ..LL.......G.L.S.SI.FKSA.IV.I.SYHDPSR.N.SVCS

DmePIWI   LIASMDLQQNS---TYFSTVTECSAFDVLANTLWPMIAKALRQYQHEHRK--LPSRIVFYRDGVSSGSLK
DmeAub    FV.T..QKESF---R.....N.HIKGQE.SEQMSVNM.C...S..EQ..S--..E..L.F....GD.Q.Y
AgaAgo4   MV.TL.HDNRG-TPKF....SHH.SGEEIS.Y.PLNTV...NE.RR.FGE--..K..I.......D.Q.Q
AaePIWI2  MV.TF.HDNRG-TPKF....SQHRHGEEIC.Y.PLNTI...NE.RK.YNE--..K..F......GE.Q.H
AaePIWI1  MV.TF.HDNRG-TPKF....SQHRHGEEIC.Y.PLNTI...NE.RK.YNE--..K..F......GE.Q.H
AaePIWI2  MV.TF.HDNRG-TPKF....SQHGHGEEIC.Y.PLNTV...NE.RK.YNE--..K..F......GE.Q.H
AaePIWI4  MV.TL.HDNKG-TPKF....SQHTHGEEIS.Y.PINTV...NE.RK.FGE--..K..I......GE.Q.H
CpiPIWI2  MV.TL.HDNKG-TPKF....SQHTHGEEIS.Y.PINTV...NE.RK.FGE--..K..I......GE.Q.H
CpiPIWI3  MV.TF.YENKG-VPK.....SQHTHGEEIS.Y.PLNTV...DE.RK.YGE--..K..I......GE.Q.H
CpiPIWI1  MV.TF.YENKG-VPK.....SQHTHGEEIS.Y.PLNTV...DE.RK.YGE--..K..I......GE.Q.H
AgaAgo5   .V.T.YAAKHI-EPK...VIERHQRGEE.SSF.SSN.V....K..EQFGEGV..R..LV.....GD.Q.G
```

```
AaePIWI5  MV...YGGGCK-HPKF....NPHTSGEE.S.FMAQNVI...HS.RN.FAG-T..Q..IV.....GE.Q.Q
CpiPIWI5  MV...YGAGQK-HPKF....NHH.SGEE.S.FMAQNVI...HS.SNDFGG-A..Q..II.....GE.Q..
AAePIWI7  .V...YGAGCR-HPK.....NHH.NGEE.S.FMAQN.I..VHS.RADFGN-A..E..IV.....GE.Q..
AaePIWI6  .V...YGGGVK-HPK.Y...NQHAYGEE.S.Y.ALNVI..I.A..SSFGN-I..Q...I.....GE.E.G
CpiPIWI4  .V.A.NHGKHRQHSEFY...NQH.YGAD.SDS.GLN.V....SF.GAFEN-N..E..II.....GD.E..
CpiPIWI6  .V.A.YHGKHRYHPNFY...NQHANGAE.SDS.ALNVV....A..KQFEN-N..G...V.....GD.D.Q
BmoPIWI   FV..YNQSMTL----WY.K.IFQEKGQEIVDG.KCCLVD..TH.LRSNGQ--..D..II.....GD.Q..

DmePIWI   QLFEFEVKDIIEKLKTEYARVQLSP-PQLAYIVVTRSMNTRFFLNGQ----NPPPGTIVDDVITLPERYD
DmeAub    .VVNS..NTLKDR.DEI.KSAGKQEGCRMTF.I.SKRI.S.Y.TGHR----..V...V............
AgaAgo4   YIYDH..RSLV...GQI.KSAGIEQDVL.TFFI.NKRI.....DHRL----..R...V..N.V...Q.T.
AaePIWI2  YVY.H...S.VD..NEI.KSAGAEQDVMFTF.I.SKRI.....DRK.----..R...V....V.N...T.
AaePIWI1  YVY.H...S.VD..NEI.KSAGAEQDVMFTF.I.SKRI.....DRK.----..R...V....V.N...T.
AaePIWI2  YVY.H...A.VD..NEI.KSAGAEQDVMFTFFI.NKRI.....DRK.----..R...V....V.....T.
AaePIWI4  YVY.H...S.V...NQI.KSAGIDQDVL.TFFI.NKRI.....DHR.----..R...V....V.....T.
CpiPIWI2  YIY.H...S.VD..NQI.KSAGIDQDVL.TFFI.NKRI.....DHR.----..K...V...MV.....T.
CpiPIWI3  YVY.H...S.V...GEV.KKFGNDQDVLFT.FI.SKRI.....ERRN----..K...V...IV.....T.
CpiPIWI1  YVY.H...S.V...GEV.KKFGNDQDVLFT.FI.SKRI.....ERRN----..K...V...IV.....T.
AgaAgo5   HVVDH..RA.K...TRV.E--NHEFKS..TV...NKRI...L.DG.R----......V...IV.....T.
AaePIWI5  YVYQH.ITAMK...NIAFK--DQPNASR.TFC..SKRI...L.QG..----..L..............N.
CpiPIWI5  YVY.H..GA.K...NMA.K--DQA-ASK.TFF..SKRIH..L.HQK.----..I.......I......N.
AAePIWI7  YVYDH..NA.K...LLACK--DRREAAR.TFF..NKRI...L.HQKR----..V..............N.
AaePIWI6  YVH.H..GAVK...EAA.K--AHEFQSK.TFF..NKRI...L.HDRR----..T..............N.
CpiPIWI4  YVH.H..GAVR.QVDRI.K--AYEKK.K..F...SKRI.....DAKS----..K...V..........N.
CpiPIWI6  YVH.Y..GA.RD.IESL.K--ACGLEAKFCF...SKRI...L.NRR.----..V..............N.
BmoPIWI   L.QQY.IP----QM.ICFTILGSNYQ.T.T.V..QKRI...I..KSRDGYD..N...V..HC..RRDW..

DmePIWI   FYLVSQQVRQGTVSPTSYNVLYSSMGLSPEKMQKLTYKMCHLYYNWSGTTRVPAVCQYAKKLATLVGTNL
DmeAub    .F....A..I..........ISDN...NAD.L.M.S...T.M...Y...I......H..H...F..AESI
AgaAgo4   ......S.K.......A...I.DTS..KIDHL.M.S..Q....................H..SF...QY.
AaePIWI2  ..I...S.........A.....DTS..KVDHL.M.S..Q....................H..SF.I.QFI
AaePIWI1  ..I...S.........A.....DTS..KVDHL.M.S..Q....................H..SF.I.QFI
AaePIWI2  ..I...S.........A...I.DTS..KVDHL.M.S..Q....................H..SF...QFI
AaePIWI4  ......S.........A...I.DTS..KIDHL.M.S..Q....................H..SF..AQF.
CpiPIWI2  ......S.........A...I.DTS..RIDHL.M.S..Q....................H..SF..AQF.
CpiPIWI3  ......S.........A...I.DTSN.KIDHL.M.S..Q....................H...F...QF.
CpiPIWI1  ......S.........A...I.DTSN.KIDHL.M.S..Q....................H...F...QF.
AgaAgo5   .F.I..S.............IFDES..K.DQL.VY.A.QT......C.SVA........H...F..SQFI
AaePIWI5  .F....S............I.RDES..TADQL.LY...QT.........VG........H...F.A.QH.
CpiPIWI5  .F....S............I.RDES..NADQL.LY.F.QT........SVG........H...F.A.QY.
AAePIWI7  ......S............I.KDES..NAD.L.LY.F.QT.M.......VG........H...A.A.QH.
```

```
AaePIWI6  ......S............I.KDES...ADRL.LY.F.QT.M.......VG........H...A.A.QY.
CpiPIWI4  ......SA..............NGSE.GADQL.VHS..QT.M....T..VQ........N...A...QF.
CpiPIWI6  ...I..N...............NGV..DVD.L.QYSF.QT.M.......VQ........H...A.A.QY.
BmoPIWI   .LI...K.T....T..H.V.V.DDS.IT.DQC.R............P..V....P....H..SY...QCV

DmePIWI   HSIPQNALEKKFYYL
DmeAub    NRA.SAG.QNQL.F.
AgaAgo4   .QT.S.M....L.F.
AaePIWI2  .QA.S.L....L.F.
AaePIWI1  .QA.S.L....L.F.
AaePIWI2  .QA.S.L....L.F.
AaePIWI4  .QA.S.L....L.F.
CpiPIWI2  .QA.S.L....LFF.
CpiPIWI3  .QS.S.L....L.F.
CpiPIWI1  .QS.S.L....L.F.
AgaAgo5   .NQ.H.M..GRL...
AaePIWI5  .QA.N.L....L...
CpiPIWI5  .QA.H.M....L...
AAePIWI7  .QA.NSL....L...
AaePIWI6  .QA.STW....L.F.
CpiPIWI4  .QP.HTR..R.LHF.
CpiPIWI6  .QP.STW....L.F.
BmoPIWI   .AQ.SDV.VD.LFF.
```

1F. Rm62 protein alignment

```
CPIJ010448  ----------------------------------------------------------------------
AGAP008601  ----------------------------------------------------------------------
CPIJ007987  ----------------------------------------------------------------------
AAEL010317  ----------------------------------------------------------------------
AGAP011084  ----------------------------------------------------------------------
AAEL013950  ----------------------------------------------------------------------
CPIJ008842  ----------------------------------------------------------------------
AGAP009808  ----------------------------------------------------------------------
AAEL008500  ----------------------------------------------------------------------
AGAP009135  ----------------------------------------------------------------------
CPIJ000753  ----------------------------------------------------------------------
AAEL013359  ----------------------------------------------------------------------
AGAP009863  ----------------------------------------------------------------------
```

```
AAEL014414  ----------------------------------------------------------------------
AGAP003089  ----------------------------------------------------------------------
AAEL009285  ----------------------------------------------------------------------
CPIJ008599  ----------------------------------------------------------------------
AGAP007511  ----------------------------------------------------------------------
AAEL011744  ----------------------------------------------------------------------
CPIJ015074  ----------------------------------------------------------------------
AGAP004711  ----------------------------------------------------------------------
CPIJ000951  ----------------------------------------------------------------------
CPIJ006204  ----------------------------------------------------------------------
AAEL001769  ----------------------------------------------------------------------
CPIJ016569  ----------------------------------------------------------------------
AGAP004912  ----------------------------------------------------------------------
AAEL002083  ----------------------------------------------------------------------
CPIJ014935  ----------------------------------------------------------------------
AGAP005652  ----------------------------------------------------------------------
AAEL008738  ----------------------------------------------------------------------
CPIJ012512  ----------------------------------------------------------------------
AGAP012045  ----------------------------------------------------------------------
AAEL002351  ----------------------------------------------------------------------
CPIJ003935  ----------------------------------------------------------------------
AGAP005351  ----------------------------------------------------------------------
AAEL010787  ----------------------------------------------------------------------
CPIJ009445  --------------------------------------------------------------------MF
AGAP003663  ------------------------------------------------------------------KRKR
AAEL004978  ----------------------------------------------------------MCDEWEDNDATG
CPIJ009286  -----------------------------------------------------------MSEWEDNDDAG
AGAP008578  --------------------------------------------------------MSGDGEWDDCD-EG
CPIJ014361  ----------------------------------------------------MDENLGG------CRFAG
AGAP003047  ----------------------------------------------------MSNAINQNGTGLEQQFAG
AAEL010402  ---------------------------------------MDECWDDDNYNSVCGNGGGGGGGGGITAARH
AAEL001317  ---------------------------------------------------------MSGNRGNFSFSMR
AGAP012523  ----------------------------------------------------------------------
CPIJ019196  ---------------------------------------------------------MSGNRGNFSFSMR
AAEL013985  MTRPGENGKDRRRSRSHS-PPDRKKRRSRSRERDRERTKTSFRFKDFGRRDRDRDRDREQRDREREKELE
CPIJ005545  ----------------------------------------------------------------------
AGAP010656  -TRPGENGKDRRRSRSKSNSPERKKKRSRSHDRSKSGASQRHR-----EEHRERERELWEREKERDRELE
CPIJ014038  ----------------------------------------------------------------------
dmeRm62     ----------------------------------------------------------------------

CPIJ010448  ----------------------------------------------------------------------
```

```
AGAP008601  ----------------------------------------------------------------------
CPIJ007987  ----------------------------------------------------------------------
AAEL010317  ----------------------------------------------------------------------
AGAP011084  ----------------------------------------------------------------------
AAEL013950  ----------------------------------------------------------------------
CPIJ008842  ----------------------------------------------------------------------
AGAP009808  ----------------------------------------------------------------------
AAEL008500  ----------------------------------------------------------------------
AGAP009135  ----------------------------------------------------------------------
CPIJ000753  ----------------------------------------------------------------------
AAEL013359  ----------------------------------------------------------------------
AGAP009863  ----------------------------------------------------------------------
AAEL014414  ----------------------------------------------------------------------
AGAP003089  ----------------------------------------------------------------------
AAEL009285  ----------------------------------------------------------------------
CPIJ008599  ----------------------------------------------------------------------
AGAP007511  ----------------------------------------------------------------------
AAEL011744  ----------------------------------------------------------------------
CPIJ015074  ----------------------------------------------------------------------
AGAP004711  ----------------------------------------------------------------------
CPIJ000951  ----------------------MSGVRKRR---SRSRERMDLDRDRGFPRDRPQQRDRNYGNDRDLDQRD
CPIJ006204  ----------------------MPRGGSRQGGGSSTALRTHARRDRGFPRDRPQQRDRNYGNDRDLDQRD
AAEL001769  ----------------------------------------------------------------------
CPIJ016569  ----------------------------------------------------------------------
AGAP004912  ----------------------------------------------------------------------
AAEL002083  -------------------MYGNRQQSSGGF---------------------------------------
CPIJ014935  -------------------MYGNRQPSAGGF---------------------------------------
AGAP005652  -------------------MYGNRPQPGNGFG--------------------------------------
AAEL008738  -----------------MQFQGNGQPHPGGFR-PRGDKPDFY----GG----------------------
CPIJ012512  -MYDNEAVCTAAASGIVMQFQGNGQPHPGGYR-PRSDKPDFYTGGAGG----------------------
AGAP012045  -----------------MQFQGTAQPHQGGFRGPRPEKNDFY----------------------------
AAEL002351  ----------------------------------------------------------------------
CPIJ003935  ----------------------------------------------------------------------
AGAP005351  ----------------------------------------------------------------------
AAEL010787  ----------------------------------------------------------------------
CPIJ009445  SSVVRRVLIAATSVCSSSTANSKSSLLCRRFTASLSN---------------------------------
AGAP003663  SEESHHQRSVGSTGGGHRKNRYREEKPSRRHEQQQPQPHRTSRKDRER----------------------
AAEL004978  KSFGQSNYG--GGDDAQDSGFAENRRGGGGFRGRGGRGGRGGRG--------------------------
CPIJ009286  KSFGQASYGDGGGDDGQENGYGERR--GGGFRGRGGRGRGGGRGR-------------------------
AGAP008578  RSFDQPKYD------ATENSVQDND--TNGFDNYQSNN--------------------------------
CPIJ014361  LDLQQQQQQH--QQLSAQDSANLKNTSGGRYVPPQLRSGRGGGGGGPE----------------------
```

```
AGAP003047  LDLQQKQQQLGGGGGSNPESGNPKHPAG-RYVPPQLRECADSGGDFQP----------------------
AAEL010402  TSVKRGGSGYDSGMSSGARSVGTRERTSFEVFTTKVPIIIGKGGVTIKRIRTESRAQVEIDDNTPGNGRS
AAEL001317  RPGQLSGAL-------RPGQQQA--QQQKSFSLNAVPPPSSLTGRGHGHPPPKFHHQ-------------
AGAP012523  ----------------------------------------------------------------------
CPIJ019196  RQGQLSASFPNASGGGRPGQQQQQQQQQKSFSLNAVPPPSSLCGRG-GYAPAKGFQQ-------------
AAEL013985  KERFKEAERKREREREREKLREQATKYAQSVSSSSGTSSTGFRQSKFDQKPPGYVEK-------------
CPIJ005545  -------------------MIYFSAQSVSSSGTGSSSGSSGFRQSKFDQKPPGFVEK-------------
AGAP010656  KEREKEAARKREREKEREKLREQATKYAQSVSSSG-------KQSKFDQMPPGMMAPPPLLPPTVVVVEK
CPIJ014038  ----------------------------------------------------------------------
dmeRm62     -----------------MMMMA---PHDRDFG--------------------------------------

CPIJ010448  ----------------------------------------------------------------------
AGAP008601  ----------------------------------------------------------------------
CPIJ007987  ----------------------------------------------------------------------
AAEL010317  ----------------------------------------------------------------------
AGAP011084  ----------------------------------------------------------------------
AAEL013950  ----------------------------------------------------------------------
CPIJ008842  ----------------------------------------------------------------------
AGAP009808  ----------------------------------------------------------------------
AAEL008500  ----------------------------------------------------------------------
AGAP009135  ----------------------------------------------------------------------
CPIJ000753  ----------------------------------------------------------------------
AAEL013359  ----------------------------------------------------------------------
AGAP009863  ----------------------------------------------------------------------
AAEL014414  ----------------------------------------------------------------------
AGAP003089  ----------------------------------------------------------------------
AAEL009285  ----------------------------------------------------------------------
CPIJ008599  ----------------------------------------------------------------------
AGAP007511  ----------------------------------------------------------------------
AAEL011744  ------------------------------MSADLPPVKRYRREEKDSDASDHEENDDKYVPYVP-----
CPIJ015074  -----------------------------MASAAPPPVKRYRRESKESAESDAED-DDKYVPYVP-----
AGAP004711  --------------------------------MSSPRVKRYRR---DSQKSEDEE-DNSFVPYVS-----
CPIJ000951  NRGRGHRSPPPLRGDRGDRGQDLGGRKRSRSRERERDDKRKRDERKSEADPDVEEVKAEVKPTVGGSKKE
CPIJ006204  NRGRGHRSPPPLRGDRGDRGQDLGGRKRSRSRERERDDKRKRDERKSEADPDVEEVKAEVKPTVGGSKKE
AAEL001769  -----------------------------------MNRRGGRDYDDRGGRGGGGS---------------
CPIJ016569  ----------------------------------------------------------------------
AGAP004912  ----------------------------------------------------------------------
AAEL002083  -------------------------------------RGGNQ----GGGRPMNGG---------------
CPIJ014935  -------------------------------------RGGRP----GGGAGGGGG---------------
AGAP005652  -------------------------------------RGGMH----GGGRP--GG---------------
AAEL008738  --------NGVPGGKPPHFMSKPGG-PPSIPPFNPNGFGGPKPMYGSGPNGPSGG---------------
```

```
CPIJ012512  --------AGGPGGKP-HYMNKPGGGPPSIPPFNPNGFGGPKPMYGNGPNGANAGGA-------------
AGAP012045  --------VGQPGTKPPHFMNKQGP--PSIPPFNANAFGGPKPMYQQNAINGVGP---------------
AAEL002351  ----------------------------------------------------------------------
CPIJ003935  ----------------------------------------------------------------------
AGAP005351  ----------------------------------------------------------------------
AAEL010787  ----------------------------------------------------------------------
CPIJ009445  --------------------------RLLRRPEVQSAAGSYSQLRFYNQQVPAPDNPEGSD---------
AGAP003663  --------ERERERERDRERERERERERDRERERERERDREREQREHRPEVTKRHSPIKNDSHGHLIYQP
AAEL004978  --------------------------GRGGGRSDFG--GGDNDGEYQNGYSRGGG---------------
CPIJ009286  -----------------------GGGGFGGGRS-YGENGDDNNGYGGSGNSYGGG---------------
AGAP008578  --------------------------GFGDEYQ-----SNDNGGYGGGDDGYGGG---------------
CPIJ014361  ------------NDQHRGGDYSGGHSGGGGGGGRYSDRGGDRGDYYNNRRGGGGGRF-------------
AGAP003047  ------------ADTGAG---VGPVSGGGIGGG-----GGRRGGYGDSNRNRGGGDF-------------
AAEL010402  TIHIEGFTQDVSRARDAIFGVINDNARGGGGGGGRGGSGRSSNSGWGGDGSRGGFGR-------------
AAEL001317  --HQQQAASGVSKHGYHTMDAIAAYANPASQYSLGKRRGKTEDDYFDDDDE-SGQQLDYIPAP-------
AGAP012523  ------------------MESLHQHSN-ASQYSIVKRRGRTEDEYFDEDDEPATQQLEYIPAP-------
CPIJ019196  --HQQQPASGVSKHGYHTMDAIAAYSNPASQYTLGKRRGKTEDDYFEDEEE-PAPQLDYIPAP-------
AAEL013985  --EIKKPIKEEPKEKEETFDLSVPMDKEEEQRRLEQEMVKRRERIERWRAERKRKEIEIKKPT-------
CPIJ005545  --EVKKPPKEEPKEEKESFDLAGPMDKEEEQRRLEQEMIKRRERIERWRAERKRKEVEIKKPG-------
AGAP010656  LAEEKKVFRERPKEEVYEVPVA-PMDKEEEQRRLEQEMTKRRERIERWRAERNKKEQEIKKPLPT-----
CPIJ014038  ----------------------MDTSDLFRKLTCGVKFSAKNNAFLKRKHNADKR---------------
dmeRm62     ---------------------HSGR----------GGRGGDR----GGDDRRGGG---------------

CPIJ010448  ----------------------MGRKNKQKIKPQLGAEKPTSNGKASVE---------------------
AGAP008601  ----------------------..K......S.VINGN--AK..H....---------------------
CPIJ007987  ---------MKKDKKSTSAKVVKKVAPALVPEEGKFDLIK.I.EDDE..---------------------
AAEL010317  ---------MKINKKAP-TKSVKKTADVVDK---KYDLIK.I.EDEE..---------------------
AGAP011084  ----------KLTH--------------------QLDFMK.LEDD.E..---------------------
AAEL013950  -------------------------------------MSSSESENGNLT---------------------
CPIJ008842  ----------------------------------------------------------------------
AGAP009808  ------------------------------------FG.MS.SESDGMS---------------------
AAEL008500  --------------------------MMTETLNSNNHLSQKGEN.MDDM---------------------
AGAP009135  ----------------------------IYSLFSEN-------N.VGDM---------------------
CPIJ000753  --------------------------MMTETLNSNNHLGQKG-E.VDDM---------------------
AAEL013359  ---------------------------------------------MGDN---------------------
AGAP009863  ---------------------------------------------MDDN---------------------
AAEL014414  ---------------------------------------------M.GR---------------------
AGAP003089  ---------------------------------------------M.GR---------------------
AAEL009285  ---------------------------------------MDTEDPNVLP---------------------
CPIJ008599  ---------------------------------------MDLDDPNVIP---------------------
AGAP007511  ---------------------------------------MSALALDE.P---------------------
```

```
AAEL011744  --------VKERKKQQLLKMGRIVQLTAEASNVGKSSSENEHDDENAE.---------------------
CPIJ015074  --------VKERKKQQLLKMGRIVQLTAEASNVGKSSSENEHDEENAE.---------------------
AGAP004711  --------VKERKKQQLLKLGRIVQLTAEASTVGKSSSENEHEEESTE.---------------------
CPIJ000951  PLSLEELLAKKKAEEEARSKPKFIT.EQRAAEALKRRQEEVAAM..AQA---------------------
CPIJ006204  PLSLEELLAKKKAEEEARSKPKFIT.EQRAAEALKRRQEEVAAM..AQA---------------------
AAEL001769  ------------------------------------------..LMRND---------------------
CPIJ016569  --------------------------------------------MRRND---------------------
AGAP004912  --------------------------------------------MRRND---------------------
AAEL002083  ---------------------------------------GFRG.DRPSG---------------------
CPIJ014935  ---------------------------------------GFRG.DRPSG---------------------
AGAP005652  ---------------------------------------.MGG.FRREG---------------------
AAEL008738  ---------------------PGRFNKFRPNMYGGP--TELGG.M.PKK---------------------
CPIJ012512  -------------------PGGG.FNKFRSNNFGGPGGVGMGG.MIPKK---------------------
AGAP012045  ----------------------G.FNKFGG---GGNRAFGGQP.TMPKK---------------------
AAEL002351  --------------------------------------MRKRG.YRRSR---------------------
CPIJ003935  --------------------------------------MRRRG.FRRSR---------------------
AGAP005351  ---------------------------------------MSR..RRRSR---------------------
AAEL010787  ---------------------------------MAPRFER--DDYKKDS---------------------
CPIJ009445  ------------------------------DFDMAPRFER--DDYKKDS---------------------
AGAP003663  GDVVHNRYKLLSTLGEGTFGRVVKA.DVE.EHTIALKIIRNVDKYRKTAKLEINVLEEIIAKDPAGRHLC
AAEL004978  -------------------------GGYGGDDDAN.H.NGFGG.DRGG----------------------
CPIJ009286  -------------------------G.GYGDND.D.G.NGYE..DRGG----------------------
AGAP008578  -------------------------GRG-------------GR.GRGG----------------------
CPIJ014361  -------------------------NDRKDNYNRG.GYNNRGG.GGGGGHPDQQQQQHLH----------
AGAP003047  -------------------------GRYGDRDYRG.DFNRR.G.GGGGRYQNDRRG--------------
AAEL010402  -------------------------DRDRDHDSRSFYNSGSGG.GGGGRDRGSNDFYNSG----------
AAEL001317  -----------------------.SPSASGSQAK------SDDEDDEDD---------------------
AGAP012523  -----------------------.SPTAADEI.KN-----I.DEEDEED---------------------
CPIJ019196  -----------------------.SPSASGSQSNRKGAGEDDDDEDDDD---------------------
AAEL013985  ---------VLTGVNIPKKWSLEDDEEDDE-DDIKDKAN-NNDEEDD.D---------------------
CPIJ005545  ---------GGSGINVPKKWSLEDDEEDDD-DDGE.KGS-AND.EDD.D---------------------
AGAP010656  --------IVPVSVSGAKKWSLEDDEEDDETEDAKDKADGAAEEEEEID---------------------
CPIJ014038  -------------------------EPSA.PA.EVKL.IKHEDDDSEN.---------------------
dmeRm62     ----------------------G.GNRFGG---------GGGG.DYHGI---------------------

CPIJ010448  ----------------------------------------------------------------------
AGAP008601  ----------------------------------------------------------------------
CPIJ007987  ----------------------------------------------------------------------
AAEL010317  ----------------------------------------------------------------------
AGAP011084  ----------------------------------------------------------------------
AAEL013950  ----------------------------------------------------------------------
```

```
CPIJ008842  ----------------------------------------------------------------------
AGAP009808  ----------------------------------------------------------------------
AAEL008500  ----------------------------------------------------------------------
AGAP009135  ----------------------------------------------------------------------
CPIJ000753  ----------------------------------------------------------------------
AAEL013359  ----------------------------------------------------------------------
AGAP009863  ----------------------------------------------------------------------
AAEL014414  ----------------------------------------------------------------------
AGAP003089  ----------------------------------------------------------------------
AAEL009285  ----------------------------------------------------------------------
CPIJ008599  ----------------------------------------------------------------------
AGAP007511  ----------------------------------------------------------------------
AAEL011744  ----------------------------------------------------------------------
CPIJ015074  ----------------------------------------------------------------------
AGAP004711  ----------------------------------------------------------------------
CPIJ000951  ----------------------------------------------------------------------
CPIJ006204  ----------------------------------------------------------------------
AAEL001769  ----------------------------------------------------------RNGGR-------
CPIJ016569  ----------------------------------------------------------RNGGR-------
AGAP004912  ----------------------------------------------------------RNGGR-------
AAEL002083  ----------------------------------------------------------GSRGG-------
CPIJ014935  ----------------------------------------------------------GFGG--------
AGAP005652  ----------------------------------------------------------GAGG--------
AAEL008738  ----------------------------------------------------------DFGGPKMFSTGA
CPIJ012512  ----------------------------------------------------------DFGGPKLYGGPS
AGAP012045  ----------------------------------------------------------DFGGPKMYGPG-
AAEL002351  ----------------------------------------------------------SRSPR-------
CPIJ003935  ----------------------------------------------------------SRSPR-------
AGAP005351  ----------------------------------------------------------SRSPR-------
AAEL010787  --------YRG----------------------------------------------VKRTGTEFK----
CPIJ009445  --------YRG----------------------------------------------VKRTGTEFK----
AGAP003663  ILMLDWFDYHGHICIAFEMLGQSVYDFMKDNKYQPFPMEQVRHMSYQLCFAVNFLHSIKLTHTDLKPENI
AAEL004978  ------------------------------------------------------FRGRGRGGRGGRGGR-
CPIJ009286  ------------------------------------------------------FRGRGRGGRGGRGGR-
AGAP008578  ------------------------------------------------------GRGRGRG----RGGR-
CPIJ014361  ----------------------------------------------------QELPPQDQLPPQEFAEN-
AGAP003047  ----------------------------------------------------GDYGNYGRGGGDRGYNN-
AAEL010402  ----------------------------------------------------SSYGGGGGGRDQNRGGND
AAEL001317  ----------------------------------------------------------------------
AGAP012523  ----------------------------------------------------------------------
CPIJ019196  ----------------------------------------------------------------------
```

```
AAEL013985  ----------------------------------------------------------------------
CPIJ005545  ----------------------------------------------------------------------
AGAP010656  ----------------------------------------------------------------------
CPIJ014038  ----------------------------------------------------------------------
dmeRm62     ----------------------------------------------------------RNGR--------

CPIJ010448  ---------------------DLEENFELEPEQTKADKRKKQKKRKNSE---------------------
AGAP008601  ---------------------.....Y.A.EATP.SKQKL.K.NKIPQQ---------------------
CPIJ007987  ---------------------N.S.ESDV.V.YQPSKLK.QKQGDFD.GFKFVSSVSEYNHDTWDDLMKF
AAEL010317  ---------------------..S.ESDV.V.YQPTKLKNQK.GDFDNDFKFVSSVSEYNHDTWDDLMKY
AGAP011084  ---------------------..S.ES.T.I.YQPTKQKNQKLTDFDGGFQFVSSVKEYNHDTWDDLMKF
AAEL013950  ---------------------....ENQVNAD-SDSNESQQ-----------------------------
CPIJ008842  ----------------------------------------------------------------------
AGAP009808  ---------------------.V..AARAAANGDDSSDQEE-----------------------------
AAEL008500  ---------------------GWKAKLK.P--PKDNR---------------------------------
AGAP009135  ---------------------GWKAKLKIP--PKDTR---------------------------------
CPIJ000753  ---------------------GWKAKLK.P--PKDTR---------------------------------
AAEL013359  ---------------------RQ.QTYDGPAGMQPDG---------------------------------
AGAP009863  ---------------------RQ.QTYDGPAGMNPDG---------------------------------
AAEL014414  ---------------------RMPA.ED----LSNVE---------------------------------
AGAP003089  ---------------------RVPA.ED----LSNVE---------------------------------
AAEL009285  ---------------------GFSIENADLDFDDDD.---------------------------------
CPIJ008599  ---------------------GFSVENVDLDYDDDEP---------------------------------
AGAP007511  ---------------------GFSLHKAEVDYDDEGN---------------------------------
AAEL011744  ---------------------AWGRK.NISLLDQHTELK.IAEAK.I.AVEKQLKEE-------------
CPIJ015074  ---------------------AWGRK.NISLLDQHTELK.IAEAK.I.AVEKQLKEE-------------
AGAP004711  ---------------------SWGRK.NISLLDQHTELK.IAEAK.I.AVEKQLKEE-------------
CPIJ000951  ---------------------SNVPK.GDV.VTALLNRE.RDPLE.YDRRERERERERERMERIRNRERE
CPIJ006204  ---------------------SNVPK.GDV.VTALLNRE.RDPLE.YDRRERERERERERMERIRNRERE
AAEL001769  ---------------------------------IEKRFD.QERNGE.----------------LRPVRWD
CPIJ016569  ---------------------------------VEKRFDRQERNGE.----------------LRAIRWD
AGAP004912  ---------------------------------VEKRFDRMERNGE.----------------LRSIRWD
AAEL002083  ----------------MGGGMGGGGG.GG----NRGTF-DR.ANNGA---------------TLRTLKWT
CPIJ014935  ------------------GGFGGG----A----NRGSFGDR.ANNGA---------------NLRSIKWT
AGAP005652  --------------------YGGG---------NGGSF-DR.SQNGK---------------NLRNVKWE
AAEL008738  PVGMGAMNGKPM-YDKPFDAFGGPKKYNT----MPNSYGN--RNGYG-PKPDFNNMSKEERAKIQSLKAK
CPIJ012512  FPG----NGNPR-FDKPMDSFGGPKKYNP----MPNSYGGGMRNSYG-PKPDYNNLTKEDRAKVQSLKAK
AGAP012045  -------GSKPYGDDKPFDTFGGPKKYPTTNPGMGGGYGMGNRNGYGGMRSDYNGFNKDDRAKIQSLKAK
AAEL002351  ---------------------------------RGGGGGT.RYDVS------------KAQLMLKPVNWN
CPIJ003935  ---------------------------------RGGGGGYQRA..SE----------YGKEMLLKAVNWR
AGAP005351  ---------------------------------GRGGLPGSRYEIG-------------KLQTLRPVQWS
```

```
AAEL010787  ---------------RPSGRDG-RDGGG--FR---GGAGGSRFGG---SSGGGRDKWTMESKPLQKINWS
CPIJ009445  ---------------RPAGRDGDRNGGGGGFRSGGGGGGGGRFGGGGRDGGGGRDKWSMDGKQLQKINWS
AGAP003663  LFVNSEYNTVVSRTTRKNRELRHVNCSDIRLIDFGNAIFDHEYHSTIVSTRHYRAPEWADAGGMTKIDWS
AAEL004978  -------------------GGGRS-D.GGGDNEGENGFGRGGGGGGFRSRNDDENNENGTDDQVKTEK--
CPIJ009286  -------------------GGGGGRD.GGGDNGDEGGFGRGGGGGGFRPRQDDNNNEDG---EVKIDK--
AGAP008578  -------------------DGGGG--.GGGGYGD-----RNGDGG----RPAYSGNSDPSMDQVKTDK--
CPIJ014361  -------------------GAG.PVG-.PGDD.FNDGVPQQHQQQPRGG-GNWNNGPRGGPRDFRDNR--
AGAP003047  -------------------GGGFQRGGDGGGY.NSEGFGGDRRDNNWGSYGGARGGPGGGPAAVGRDRPM
AAEL010402  FYN---------------SGSGFGGGRGGRSAAPVSNNFGQ.ENGRPKSPELIDWDALNKQCDEATARQW
AAEL001317  ------------------PLDAFMAGINAQV.RE.KKIPQPNVDP.KGTRGDIDDVDDEESYYRYMEENP
AGAP012523  ------------------PLDAFMAGI.AQVKME.NKIPQPHLEP.KGTRGDIDDEDDEESYYRYMQENP
CPIJ019196  ------------------PLDAFMAGI.AQV.RE.KTVPAPNVDP.KGTRGDIDDEDDEESYYRYMEENP
AAEL013985  ------------------PLDAFMKEVNE.VRKVN-KLPSSAP.Q-DGKAASNSGVTIITGVAKKNTETT
CPIJ005545  ------------------PLDAFMKEVNE.VRKVN-KITGPAP.AAEGKAATSSGVTIITGVAKKNTETV
AGAP010656  ------------------PLDAFMKEVNE.VRKVN-KLSNPLP.T-DGKASS-SGVTIITGVAKQKQE-T
CPIJ014038  ---------------------AAPIFPHSPLLLSEEEIKDEI.SEEEDDGDGDQNQQ-------------
dmeRm62     -------------VEKRRDDRGGGNR.GG-----GGGFGD--RRGGG-------------GGGSQDLPMR

CPIJ010448  -GEN--------VVKRPKNS-----DEEENDDAEDEN---------------------------------
AGAP008601  -E.DEQDVAVGRG.....DDSDDEDE.QQ.G.EA.S.---------------------------------
CPIJ007987  VKKKNRGKVDDKIANVI.GRSGKNAEVLNG..EV.PD---------------------------------
AAEL010317  VKKKNRGKVEDKIAGVI.GRSKENAEML.EA.DH.P.---------------------------------
AGAP011084  VKRKNRGEVNDKIANVIRDRTKENA.ALKD..E..DED--------------------------------
AAEL013950  -----------------------NGVSS.S..DS.N.---------------------------------
CPIJ008842  ----------------------------------------------------------------------
AGAP009808  -----------------------NGSGTDV.EEQ.GQ---------------------------------
AAEL008500  ------------------------IKTSDVT.TRGNE---------------------------------
AGAP009135  ------------------------VKTSDVT.TRGNE---------------------------------
CPIJ000753  ------------------------IKTSDVT.TRGNE---------------------------------
AAEL013359  ------------------------VI.SNWNETV.N----------------------------------
AGAP009863  ------------------------II.SNYSESY.N----------------------------------
AAEL014414  ------------------------FETS.DVEVLPT----------------------------------
AGAP003089  ------------------------FETS.DVEVLPT----------------------------------
AAEL009285  ------------------------VSGKKGKKKKGGG---------------------------------
CPIJ008599  ------------------------GKGGKKKKKNGGG---------------------------------
AGAP007511  ------------------------GFGGKKGKKKSGG---------------------------------
AAEL011744  ------------------------EKIL.SVAEKKALMGVAELAK-------------------------
CPIJ015074  ------------------------EKIL.SVAEKKALMGVAELAK-------------------------
AGAP004711  ------------------------EKIL.SVAEKKALMGVAELAK-------------------------
CPIJ000951  GKGKDGEGDDKVGRGAVVEENPVKDK.K.TEAIRERYLGIIKKKRRVRRLNDRKFVFDWDAAEDTSVDYN
CPIJ006204  GKGKDGEGDDKVGRGAVVEENPVKDK.K.TEAIRERYLGIIKKKRRVRRLNDRKFVFDWDAAEDTSVDYN
```

```
AAEL001769  QVK----------LEPF.KDFFTPASSVLERSRTEVCQ--------------------------------
CPIJ016569  QEK----------LDAFAKNFFKPASSVLDRSRAEV.A--------------------------------
AGAP004912  QVK----------LEAFQKNFFQPASSVLTRSRAEV.Q--------------------------------
AAEL002083  SE.----------LTPFEKDFYKPSEFIS.LSET.VKG--------------------------------
CPIJ014935  S.D----------LTPFEKNFYKPSEQIMALSET.F.A--------------------------------
AGAP005652  PED----------LTPFEKNFYQPSAGLMGLSVS.IDS--------------------------------
AAEL008738  FPGQGLVKPIWKDLEPFEKDFYVPHPNVMARTP.EVQA--------------------------------
CPIJ012512  FPGQTLTKPMWENLEPFEKDFYVPHPSVMARSVDEVQL--------------------------------
AGAP012045  YPGQNLMKPMWENLEPFQKDFYVPHPSVMGRA..EVQT--------------------------------
AAEL002351  HQK----------LESVTRLSYRPKVDFR-RSEREISE--------------------------------
CPIJ003935  DVK----------LEPVVR.TYR-AVGHR-RSERELSD--------------------------------
AGAP005351  QVK----------LDPIVREPYRSKATYR-RSEREISE--------------------------------
AAEL010787  KMQ----------LSPF.KDFYREHPAIK.RSQR.VER--------------------------------
CPIJ009445  KM.----------LVPF.KDFYREHPAIK.RSSR.VDR--------------------------------
AGAP003663  KMT----------LAPF.KDFYHENSIVR.RSQKEVDR--------------------------------
AAEL004978  ---PRELY------IP.APTENEDEMFGSGISSGINFD--------------------------------
CPIJ009286  ---PRELY------IP.PPTEDEDEIFGTGISSGINFD--------------------------------
AGAP008578  ---PRELY------IP.LPTEDESLIFGSGISSGINFD--------------------------------
CPIJ014361  --QPQNDR------WQEPPANGGGGGGGGYGGRGRDDR--------------------------------
AGAP003047  D.PPQNDR------WPEPGAAMGPPGPGSVGSGGGTGP--------------------------------
AAEL010402  AKCPKLIKN-FYNELPEVANMTPEEVS.FRCANNNIVV--------------------------------
AAEL001317  MAG-----LHD-DGSDAELEYDEDGNPIPPPRKREIDP--------------------------------
AGAP012523  HAG-----LID-EGSDAEMDYDEDGNPVPPQRRR.IDP--------------------------------
CPIJ019196  HAG-----LQDGDGSDGELEYDQDGNPIPPPRKR.IDP--------------------------------
AAEL013985  KKGELIEQNQDGLEYSSEEEQEDIK.TAA.LANKQKKE--------------------------------
CPIJ005545  KKGELIEQNQDGLEYSSEEEQEDIK.TAA.LANKQKKE--------------------------------
AGAP010656  KKGELIEQNQDGLEYSSEEEQEDIK.TAA.LANKQKKE--------------------------------
CPIJ014038  --------------..LRLMSPEKRKRY.AFRVKQLRN--------------------------------
dmeRm62     PVD-------FSNLAPF.KNFYQEHPNVA.RSPYEVQR--------------------------------

CPIJ010448  ----------------------------------------------------------------------
AGAP008601  ----------------------------------------------------------------------
CPIJ007987  ---------------------------------------SYNAEIDLSDDELKHDTMRTKEKKGRKR-EV
AAEL010317  ---------------------------------------SYHDEVDLSDDELKHDVMRVKEKKGRKR-EI
AGAP011084  ------------------------------------GNRAYNEEVDLSDDELKHDYMRVKERKGKKLTEA
AAEL013950  ----------------------------------------------------------------------
CPIJ008842  ----------------------------------------------------------------------
AGAP009808  ----------------------------------------------------------------------
AAEL008500  ----------------------------------------------------------------------
AGAP009135  ----------------------------------------------------------------------
CPIJ000753  ----------------------------------------------------------------------
```

```
AAEL013359  ----------------------------------------------------------------------
AGAP009863  ----------------------------------------------------------------------
AAEL014414  ----------------------------------------------------------------------
AGAP003089  ----------------------------------------------------------------------
AAEL009285  ----------------------------------------------------------------------
CPIJ008599  ----------------------------------------------------------------------
AGAP007511  ----------------------------------------------------------------------
AAEL011744  ------------------GIQYEDPIKTSWKPPRYILSRTDASHERVREKMRILVD--------------
CPIJ015074  ------------------GIQYEDPIKTSWKPPRYILARADVSHEKVRERMRILVD--------------
AGAP004711  ------------------GIQYEDPIKTSWTPPRYILSKPESRHEKIREKLRILTE--------------
CPIJ000951  NLYKERHHVQFFGRGNIAGIDIKEQKRKQSKFYGDLLEKRRTDAEKEQEKVRLKKVKKKEDKQKWDDRHW
CPIJ006204  NLYKERHHVQFFGRGNIAGIDIKEQKRKQSKFYGDLLEKRRTDAEKEQEKVRLKKVKKKEDKQKWDDRHW
AAEL001769  ----------------------------------------------------------------------
CPIJ016569  ----------------------------------------------------------------------
AGAP004912  ----------------------------------------------------------------------
AAEL002083  ----------------------------------------------------------------------
CPIJ014935  ----------------------------------------------------------------------
AGAP005652  ----------------------------------------------------------------------
AAEL008738  ----------------------------------------------------------------------
CPIJ012512  ----------------------------------------------------------------------
AGAP012045  ----------------------------------------------------------------------
AAEL002351  ----------------------------------------------------------------------
CPIJ003935  ----------------------------------------------------------------------
AGAP005351  ----------------------------------------------------------------------
AAEL010787  ----------------------------------------------------------------------
CPIJ009445  ----------------------------------------------------------------------
AGAP003663  ----------------------------------------------------------------------
AAEL004978  ----------------------------------------------------------------------
CPIJ009286  ----------------------------------------------------------------------
AGAP008578  ----------------------------------------------------------------------
CPIJ014361  ----------------------------------------------------------------------
AGAP003047  ----------------------------------------------------------------------
AAEL010402  ----------------------------------------------------------------------
AAEL001317  ----------------------------------------------------------------------
AGAP012523  ----------------------------------------------------------------------
CPIJ019196  ----------------------------------------------------------------------
AAEL013985  ----------------------------------------------------------------------
CPIJ005545  ----------------------------------------------------------------------
AGAP010656  ----------------------------------------------------------------------
CPIJ014038  ----------------------------------------------------------------------
dmeRm62     ----------------------------------------------------------------------
```

```
CPIJ010448  -------GHDDSEEQEEHPTVGSGDAYEVLLGSQEFESLKGK---VSDNTLKAITEMG------------
AGAP008601  -------.EGKEA....TVPSMTNS...II..NR..K..E..---...H.M...G...------------
CPIJ007987  SENDGKVEIE.ED.E--QQDFFEEAGGNE--EISS.YQMN-----L.RPLM...GFL.------------
AAEL010317  P-TDNKVEIE.E.DE--PQDFFEEAGGNE--EISS.YQMN-----L.RPLM...GVL.------------
AGAP011084  ENGGPTVEVKEDT.DGTKEDYFEEIEENANGEI.S.YQMD-----L.RPLM...GAL.------------
AAEL013950  ----------------------KNGNADSEEKQASW.DMG-----LI.TLCE.CRALK------------
CPIJ008842  --------------------------------------MG-----LI.TLCE.CKALK------------
AGAP009808  ----------------------AENGTD.S---KSW.D.G-----LI.TLCT.CRGLK------------
AAEL008500  -----------------------------------..EFC-----LKREL.MG.F.K.------------
AGAP009135  -----------------------------------..EFC-----LKRPL.MG.F.K.------------
CPIJ000753  -----------------------------------..EFC-----LKREL.MG.F.K.------------
AAEL013359  -----------------------------------.DDMH-----LKEQL.RG.YAY.------------
AGAP009863  -----------------------------------.DQMG-----LREEL.RG.YAY.------------
AAEL014414  -----------------------------------.N.MG-----LREEL.RGVYAY.------------
AGAP003089  -----------------------------------.N.MG-----LREEL.RG.YAY.------------
AAEL009285  -----------------------------------.QAMG-----L.MPI....LK..------------
CPIJ008599  -----------------------------------.QAMG-----LAMPV..G.LK..------------
AGAP007511  -----------------------------------.QAMG-----L.APV..G.LK..------------
AAEL011744  -------------------------GEN.PPPICS.REM.-----FPKAI.A.LEKRN------------
CPIJ015074  -------------------------GEN.PPPICT.REM.-----FPKSI.AGLEKRN------------
AGAP004711  -------------------------GEN.PPPLRT.REM.-----LPKAV.A.LAKRN------------
CPIJ000951  SEKEVDEMTERDWRIFREDYNVTIKGGKIPNPIRSWKESG-----FPKEV.EI.DKV.------------
CPIJ006204  SEKEVDEMTERDWRIFREDYNVTIKGGKIPNPIRSWKESG-----FPKEV.EI.DKV.------------
AAEL001769  ---------------YLDKNEITMIGKN.PAPIMQ.GESG-F---P.-VF.DEMGRQ.------------
CPIJ016569  ---------------YLDKNEITVIGKNIPAPILY..EGG-F---P.-SI.AE..RQ.------------
AGAP004912  ---------------YLDKNEITVYGKD.PAPIMH.HESG-F---PQ-YM.DEFQRQA------------
AAEL002083  ---------------YLAKLEITLKGRNIPRP.M...QGG-L---PD-YI.EEANKQ.------------
CPIJ014935  ---------------YLAKLEITLKGRDIPRPCIT.GDCG-L---PD-YI.EETVKQ.------------
AGAP005652  ---------------YLDKHQITLKGRD.PRP.M...DGG-L---PV-YIMEELKRQ.------------
AAEL008738  ---------------FRERMQITVMGNS.PHP..D..EGN-F---PD-FVMNE.NK..------------
CPIJ012512  ---------------FRENMQVTVMGNT.PHPT.T.DEGN-F---PE-FVINE.NKQ.------------
AGAP012045  ---------------FREQMQITVMGNN.PHPC.N..EGN-F---PE-YVMTE.KKQ.------------
AAEL002351  ---------------WRKTKEITTKGRD.PDPALT..EVG-F---PA-EIADEWRYAE------------
CPIJ003935  ---------------WRKSKEITIKGRDCPDPIFT..DSG-F---PA-EIVDEMRYA.------------
AGAP005351  ---------------WRRSKEITTKGHDIPDPIFT..ESG-F---PA-EIIDELRYA.------------
AAEL010787  ---------------FLEKHDITLIG-NCPKPIT..DEID-M---PD-YV.NE.EKQ.------------
CPIJ009445  ---------------FLEKHDITLIG-QCPRPIT..DEIE-M---PD-YV.SE.ERQ.------------
AGAP003663  ---------------YLAKHDITLIG-KCPKPIT..DEIE-I---PD-YVKRE.DRQ.------------
AAEL004978  ---------------KFDEIKVNVTGENPPSPIKS.GDSG-L---RD-YL.QN.RKSH------------
CPIJ009286  ---------------KFDDIKVNVTGENPPGPITS.NESG-L---RD-YL.TNVRKS.------------
```

```
AGAP008578  ---------------KFEEIQVRVSGENPPDHVES..RSG-L---RE-EVMTNVRKSS-------------
CPIJ014361  ---------------GMG-GRWNDRGGRG-ADVDFTQLTE-R---DE-RLEGELFQH.NTGINFSKYEDI
AGAP003047  ---------------G.G.GY.G.GGGSGRGQIDYTVLTE-R---DE-PLATQ.F.E-------------
AAEL010402  ---------------DRTFKDADKPSAPIPNPV.T..QAF-H---EYPEL.EE.KKQ.------------
AAEL001317  --------LPPIDHS.IDYDKFEKNF.NPHEDIVGLSLS.IN---ELR.K.G-VKVS.PA----------
AGAP012523  --------LPSIDHT.IDYLKFEKNF.IPHEDIVNLSQA.VQ---DLRL..G-VKVS.PM----------
CPIJ019196  --------LPVIYHS.IDYEKFEKNF.IPHEDIIALA.SQTQ---ELRHK.G-.KVS.PS----------
AAEL013985  --------LAKIDHSGINYMPFRKAF.VEVPEIAKMTHAEVG---.YKTE.EG..VK.KG----------
CPIJ005545  --------LAKIDHSGINYLPFRKVF.VEVPEIAKMTQTEVD---AYKAE.EG.NVK.KG----------
AGAP010656  --------LAKIDHSGINYMPFRKSF.VEVPEIARMTQTEID---AYKKE.EG.AVK.KG----------
CPIJ014038  ----------------VQQIKVKKG.IA.PDPVEG.QQ.A.EPYN..NQLI.NVS.C.------------
dmeRm62     ---------------YREEQEITVRG-Q.PNPI.D.SEVH-L---PD-YVM.E.RRQ.------------

CPIJ010448  ---------------------------------FTKMTEIQAKAIPPLLAGRDLIGSAKTGSGKTLAFLI
AGAP008601  ---------------------------------..........S.....E....................
CPIJ007987  ---------------------------------YIYP.P...AT..IA.M...VC.C.A..T...A.YML
AAEL010317  ---------------------------------YIYP.P...AT..IA.L...IC.C.A..T...A.YML
AGAP011084  ---------------------------------YIYP.P...ST..IA.M...IC.C.A..T...A.YML
AAEL013950  ---------------------------------WKAPSK..RE...LA.Q.K.V..L.E......G..AL
CPIJ008842  ---------------------------------WKAPSK..RE...LA.Q.K.V..L.E......G..AL
AGAP009808  ---------------------------------WKAPSK..RE...LA.Q.K.I..L.E......G..AL
AAEL008500  ---------------------------------WE.PSP..EA...IA.V.K.ILAR..N.T...G.YS.
AGAP009135  ---------------------------------WE.PSP..EA...IA.V.K.ILAR..N.T...G.YS.
CPIJ000753  ---------------------------------WE.PSP..EA...IA.V.K.ILAR..N.T...G.YS.
AAEL013359  ---------------------------------.E.PSA..QR..M.CIK.H.V.AQ.QS.T...AT.S.
AGAP009863  ---------------------------------.E.PSA..QR..V.CVK.S.V.AQ.QS.T...AT.S.
AAEL014414  ---------------------------------.E.PSA..QRS.L.IVK...V.AQ.QS.T...AT.S.
AGAP003089  ---------------------------------.E.PSA..QRS.Q.IVK...V.AQ.QS.T...AT.S.
AAEL009285  ---------------------------------YKVP.P..R.T..LI.E...VVAM........GC...
CPIJ008599  ---------------------------------YKVP.P..R.T..LI.E...VVAM........GC...
AGAP007511  ---------------------------------YKIP.P..R.T..II.D...VVAM........GC...
AAEL011744  ---------------------------------IR.PSP..VQG..AV.S......I.F.......V.VL
CPIJ015074  ---------------------------------IR.PSP..VQG..AV........I.F.......V.VL
AGAP004711  ---------------------------------IK.PSP..VQG..AV........I.F.......V.VL
CPIJ000951  ---------------------------------YKEP.P..RQ...IG.QN..I..I.E...........
CPIJ006204  ---------------------------------YKEP.P..RQ...IG.QN..I..I.E...........
AAEL001769  ---------------------------------.QEP.S...VGWSIAMS...MV.I..........YIL
CPIJ016569  ---------------------------------YKEP.Q...VGWSIATS...MV.I..........YIL
AGAP004912  ---------------------------------.KEP.F...VGWSIAMS...MV.I..........YIL
AAEL002083  ---------------------------------.S.P.A...QGM.IA.S...MV.I.Q........YIA
CPIJ014935  ---------------------------------...P.A...QGM.IAMT...MV.I.Q........YVA
```

```
AGAP005652  ---------------------------------.A.P.A...QGM.IA.S...MV.I.Q........YVV
AAEL008738  ---------------------------------.PNP.A...QGW.IA.S....V.I.Q........YML
CPIJ012512  ---------------------------------.PSP.A...QGW.IA.S...MV.I.Q........YML
AGAP012045  ---------------------------------.PRP.A..SQGW.IA.S...MV.I.Q........YML
AAEL002351  ---------------------------------..TP.P..SQGW.IAMS...MV.I.........SY.L
CPIJ003935  ---------------------------------..AP.P..SQGW.IA.S...MV.I.........SY.L
AGAP005351  ---------------------------------..TP.P...QGW.IA.S...MV.I.........SY..
AAEL010787  ---------------------------------.QRP.P...QGW.IA.S.LNMV.V.........GYML
CPIJ009445  ---------------------------------YQRP.P...QGW.IA.S.LNMV.V.........GYML
AGAP003663  ---------------------------------YKSP.P...QGW.IA.S.LNMV.V..........YML
AAEL004978  ---------------------------------Y..P.P..KY...IIMDK...MAC.Q......A...L
CPIJ009286  ---------------------------------YL.P.P..KY...IIMDK...MAC.Q......A...L
AGAP008578  ---------------------------------Y..P.P..RY...II.N....MAC.Q......A..ML
CPIJ014361  PVEATGDNVPPHINTFDDIELTEIIENNIKLANYDVP.PV.KY...IVMS...VMAC.Q......A...V
AGAP003047  ----------------------------------------------------------------------
AAEL010402  ---------------------------------.A.PSP...Q.W.V..K.E....I.Q..T.......L
AAEL001317  --------PPAPVTSFAHFGFDEQLMKAIRKSEY.QP.P...QGV.AA.S...I..I........A...W
AGAP012523  --------PPHPVTSFAHFGFDESLMKSIRKSE.STP.P...Q...AA.S...I..I........A...W
CPIJ019196  --------HAR-----------------------------SPQGV.TA.S...I..I........A...W
AAEL013985  --------CPKPIKTWAHCGVSKKEFDVLRKLG.E.P.P..CQ...AIMS......I...........IL
CPIJ005545  --------CPKPIRTWAHCGVTRKEFEVLRKLG.E.P.P..CQ...AVMS......I...........IL
AGAP010656  --------CPKPIKTWAHCGVSRKEFEVLRKLG.E.P.P..CQ...AIMS......I...........IL
CPIJ014038  ---------------------------------YRAP.PV.MQ...V..E.HP.HAC.P......A....
dmeRm62     ---------------------------------YKAP.A...QGW.IAMS.SNFV.I.........GYIL

CPIJ010448  PAVELIYKLQFKPRN-----GTGVLVISPTRELAMQIFGVLKELSAHHH------YTYGLLMGGASRHTE
AGAP008601  ......H..R.....-----.A..I...................MTY.C------Q..............
CPIJ007987  .TL.RLLYKPSAAQA-----V.R...LV.....GA.VYQ.T.Q.TQYTS------IEV.IAI..LDVKAQ
AAEL010317  .TL.RLLYKPSAAQA-----V.R...LV.....GA.VYQ.S.Q.TQFTS------IEV.IAI..LDVKAQ
AGAP011084  .TI.RLLYKPNVAQA-----V.R...LV.....GA.VYQ.A.Q.TQFTN------VDV.IAI..LDVKAQ
AAEL013950  .ILQALLENP----Q-----RYFAVILT......Y..SEQFEA.G.SIG------IKCCVIV..MDMVSQ
CPIJ008842  .ILQALLENP----Q-----RYFAVILT......Y..SEQFEA.G.SIG------VKCCVIV..MDMVSQ
AGAP009808  .ILQALLDNP----Q-----RYFAV.LT......Y..SEQFEA.G.TIG------VKCCVIV..MDLV.Q
AAEL008500  .VL.Q.DPTKDYIQ---------A.I.V......L.TSQICI..AK.M.------IRVMVTT..TNLKDD
AGAP009135  .VL.QVDPTKDYIQ---------A.I.V......L.TSQICI..AK.MN------IRVMVTT..TNLKDD
CPIJ000753  .VL.Q.DPTKDYIQ---------A.I.V......L.TSQICI..AK.M.------IRVMVTT..TNLKDD
AAEL013359  AILQQ.DTSIAECQ---------A.ILA......T..QK.VIA.GDYLG------AQCHACI..TNVRDD
AGAP009863  AILQQ.KTEIPDCQ---------A.ILA......S..QK.VIS.GDFLK------AQCHACI..TNVRDD
AAEL014414  AILQSMDTTLRETQ---------..CL.......V..QK.ILA.GDFMN------VQCHACI..TNLGED
AGAP003089  SILQSMDTTLRETQ---------..CL.......V..QK.ILA.GDFMN------VQCHACI..TNLGED
AAEL009285  .LF.KLKQREI.SG-------ARA..LT......I.T.KFI.Q.GKFTD------LKTI.VL..D.MDSQ
```

```
CPIJ008599  .LF.KLKQREI.KG-------ARA.IL.......I.T.KFI.Q.GKFMD------LKTI.VL..D.MD.Q
AGAP007511  .MF.RLKQREA.AGG-----.ARA.IL.......I.TYKFI.Q.GRFMD------LKAI.VL..D.MDSQ
AAEL011744  .I.MFSLEQ-ELRLPFISKE.PYG.I.C.S....K.THDIIQYYCQ.LQMSGMPEIRSA.AI..VPVNDA
CPIJ015074  .I.MFSLEQ-EIRLPFMAKE.PYG.I.C.S....K.T.DIVQYYCQ.LQQAGMPEIRSA.AI..VPVNEA
AGAP004711  .IIMFCLEQ-ELRLPFIKRE.PYG.I.C.S....K.THDIIQYYCR.LQEAGMPEIRTV.AI..VPVNDA
CPIJ000951  .LLNW.QG.PKIE.QETADQ.PYAIILA......Q..EEETQKFGQPLG------IRTVVVV..L..EEQ
CPIJ006204  .LLNW.QG.PKIE.QETADQ.PYAIILA......Q..EEETQKFGQPLG------IRTVVVV..L..EEQ
AAEL001769  ..LIH.SN--QPRLL--RGD.PIA..LA......Q..QQ.CNDFGRRMS------IMNTCIF....K.PQ
CPIJ016569  ..LIH.SN--QPRLM--RGD.PIA..LA......Q..QQ.CDDFGRRMS------VMNTCIF....KMGQ
AGAP004912  ..LVH.SN--QPRIA--RGD.PIA..LA......Q..KQ.CDDFGRRMG------IYNTCVF....KYPQ
AAEL002083  ..LVH.TH--QDQLR--RGD.PIA..LA......Q..QQ.ATDFGQRIN------ANNTCVF...PKGPQ
CPIJ014935  ..LVH.QH--QETVH--RGD.PIA.ILA......Q..QQ.ANDFGQRTN------TNNTCVF...PKGPQ
AGAP005652  .SLVH.QH--QATIR--RGD.PIA.ILA......Q..QQ.ATDFGSRVS------ANNTCVF...PKGPQ
AAEL008738  .GIVH.AH--Q..LQ--RGE.PV...LA......Q..QT.VRDFGT.SKP----LIR.TCIF...LKGPQ
CPIJ012512  ..IVH.AH--Q..LQ--RGD.PI...LA......Q..QT.VRDFGT.SKP----NIR.TCIF...LKGPQ
AGAP012045  .GLVH.SH--Q..LS--RGE.PI...LA......Q..QT.VRDFGN.SKP----NIR.TCVF...LKGPQ
AAEL002351  ..LMH.DQ--QSRLR--RGD.PIA.ILA......Q..KQ.TDDFGRAMK------IKNTC.F..GAKRQQ
CPIJ003935  ..MLH.EQ--QSRIR--RGD.PIA.ILA......Q..KQ.AD.FGRPVK------IKNTC.F..GAKRQQ
AGAP005351  ..LIH.DQ--QPRLR--RGD.PIA.ILA......Q..KQ.ADDFGRALK------.KNTC.F..GKKRKQ
AAEL010787  ..IVH.NH--Q..DP--SVR.PL...LA......Q..QQ.ATDFGSSSY------IRNTC.F..S.KGPQ
CPIJ009445  ..IVH.NH--Q..DP--NIR.PL...LA......Q..QQ.AT.FGSSSY------IRNTC.F..S.KGPQ
AGAP003663  ..IVH.NH--Q..DP--SVR.PL...LA......Q..QQ.AT.FGSSSY------IRNTC.F..S.KGPQ
AAEL004978  .MINTLLN--DNADM--VPGNPF.VI.A......L...NEARKFALGT------VLKVCVAY..TATRHQ
CPIJ009286  .IINTLLN--DNDDM--TPGNPF.V.VA......L..SEEARKFARGT------ILKVVVAY..TATRHQ
AGAP008578  .MIHHLLDKEDSLEL--RTRNPYIVIVA......I..HDEGRKFAHGT------KLKVCVSY..TAVQHQ
CPIJ014361  .ILNQM..--HGVTP--PPQNRPFNRRKQYPLGLVLAPTQS.KFCYRS------RMRPCV.Y..NNTQEQ
AGAP003047  ----------------------------------------S.KFCYRS------RMRPAV.Y..NNTQDQ
AAEL010402  ..FIH.EGQPVPRGE--ARG.PN...MA......L..EKEVFKYQFR-------DIKAIC.Y..GD.R.Q
AAEL001317  .MLVH.MDQKELGPG----D.PIG.ILA.....SL..YQEA.KFGKIYN------ISVCCCY..G.KWEQ
AGAP012523  .MLVH.MDQRELGPG----D.PIG.ILA.....SL..YNEA.KFGKVYN------ISICCCY..G.KWEQ
CPIJ019196  .MLVH.MDQKNLGPG----D.PIG.ILA.....SL..YQEA.KFGKVYN------ISVCCCY..G.KWEQ
AAEL013985  .MFRH.LDQPPLEDG----D.PISIIMT.....C...GKDI.KFAKSLN------LRAVCVY..TGISEQ
CPIJ005545  .MFRHVLDQPPLEEA----E.PIAIIM......C...GKDI.KFAKSLN------LRAVCVY..TGISEQ
AGAP010656  .MFRHLLDQPPLEDG----D.PIAIIMT.....C...GKDI.KF.KSLN------LRTACVY..TGISEQ
CPIJ014038  .IIHHLK.PMKCGFR--------A.IVC......K.TQREALR.CEEIN-----LR.HVITKVDENTTDY
dmeRm62     ..IVH.NN--QQ.LQ--RGD.PIA..LA......Q..QQ.AT.FGSSS------YVRNTCVF...PKGGQ

CPIJ010448  NEKLSKGLNIIVATPGRLLDHLKGTP--NFLFKNLQCLIIDECDRILEIG---FEEDMKQIISILPKK--
AGAP008601  ....E..I...............S.--...................---....L..........--
CPIJ007987  EAV.RTNPD.VI......I..I.N..--S.SLDSIEV..L..A..M.DEY---.A.Q..E..QSCS.S--
AAEL010317  EAV.RTNPDVVI......I..I.N..--S.SLDSIEV..L..A..M.DEY---.A.Q..E..QSCS.T--
```

```
AGAP011084  EAV.R.NPDVVI......I..I.N..--S.SLDSIEI..L..A..M.DEY---.A.Q..E..RSCSAT--
AAEL013950  ALH.ARKPH..I......V...EN.K--G.NL.AVKY.VM..A....NLD---..VELEK.LKVI.RE--
CPIJ008842  ALQ.ARKPH..I......V...EN.K--G.NL.AVKY.VM..A....NLD---..VELDK.LKV..RE--
AGAP009808  AIQ.ARKPH..I......V...EN.K--G.SL.AIRY.VM..A....NMD---...EVNK.LKVM.RE--
AAEL008500  IMRIYQKVQV.I.....I..LMD-KE--VANMA.CRM.VL..A.KL.SQD---.KGMLDHV.MK...E--
AGAP009135  IMRIYQKVQV.I.....I..LMD-KE--VANMSQCRM.VL..A.KL.SQD---.KGMLDHV.MR...E--
CPIJ000753  IMRIYQKVQV.I.....I..LMD-KE--VANMA.CRM.VL..A.KL.SQD---.KGMLDHV.MK...E--
AAEL013359  MR..EM.CH.V.G....VH.MIS-RN--VLRPSHIKLFVL..A.EM.SR.---.KDQIQDVFRM..ND--
AGAP009863  MRR.EQ.CH.V.G....VH.MIS-RN--VLQP..IKMFVL..A.EM.SR.---.KDQIQDVFQK..AD--
AAEL014414  IR..DY.QHVVSG....VF.MI.-RR--VLRTRSIKM.VL..A.EM.NK.---.K.QIYDVYRY..PA--
AGAP003089  IR..DY.QHVVSG....VF.MI.-RR--VLRTRSIKM.VL..A.EM.NK.---.K.QIYDVYRY..PA--
AAEL009285  FAAIHTLPD........F.HLCV-EM--DLKLSSV.YCVF..A..LF.M.---.G.QLTETLRR..EA--
CPIJ008599  FAAIHTLPD........F.HLCV-EM--DLKLSAIKYCVF..A..LF.M.---.G.QLTETLAR..SS--
AGAP007511  FAAVHTLPDV.......F.HLCV-EM--DLKLNSV.YCVF..A..LF.M.---.G.QLTET.KR..ES--
AAEL011744  LATIQQ.CH.M.......M.M.D-KK--LLKLDVCRY.CM..A..MIDA.-------..IRT.F.YFKGQ--
CPIJ015074  LAVIQQ.CH.M.......M.M.D-KK--LVKLDVCRY.CM..A..MIDM.-------..VRT.F.YFKGQ--
AGAP004711  IAIIQQ.AH.M.......M.M.D-KK--LVTLDVCRY.CM..A..MIDM.-------..VRT.F.YFKGQ--
CPIJ000951  GFR.RL.CE.VI......I.V.E-NR--YLVLNQCTYIVM..A..MIDM.----..P.VQK.LEYM.VTNL
CPIJ006204  GFR.RL.CE.VI......I.V.E-NR--YLVLNQCTYIVM..A..MIDM.----..P.VQK.LEYM.VTNL
AAEL001769  ADD.RR.VE.VI......I.F.E-SG--TTNLRRTTY.VL..A..M.DM.----..PQIRK...QIRP---
CPIJ016569  AND.RR.VE.VI......I.F.E-SG--TTNLRRTTY.VL..A..M.DM.----..PQIRK...QIRP---
AGAP004912  ESD.RR.VE.VI......I.F.E-RE--TTNLRRCTY.VL..A..M.DM.----..PQIRK...QIRP---
AAEL002083  IRD.ER.AE.VI......I.F.E-RG--ITNLRRCTY.VL..A..M.DM.----..PQIRK.MGQIRP---
CPIJ014935  IRD.ER.AE.VI......I.F.E-RG--ITNLRRCTY.VL..A..M.DM.----..PQIRK.MGQIRP---
AGAP005652  IRD.ER.AE.VI......I.F.E-RG--ITNLRRCTY.VL..A..M.DM.----..PQIRK.MGQIRP---
AAEL008738  VRD.ER.VEVVI......I.F.E-RG--ITNLRRCTY.VL..A..M.DM.----..PQIRK..EQIRP---
CPIJ012512  VRD.ER.VEVVI......I.F.E-RG--ITNLRRCTY.VL..A..M.DM.----..PQIRK..EQIRP---
AGAP012045  VRD.ER.VEVVI......I.F.E-RG--ITNLRRCTY.VL..A..M.DM.----..PQIRK.VEQIRP---
AAEL002351  GDD.KY.VE.VI......I.F.S-SE--HTNLRRCSY.VL..A..M.DM.----..PQIRA..EQIRP---
CPIJ003935  SQD.EY.VE.VI......N.F.S-SN--HTNL.RCSY.VL..A..M.DM.----..PQIRA..GQIRP---
AGAP005351  QDD.EY.VE.VI......I.F.S-SN--QTNLRRCSY.VL..A..M.DM.----..PQIRT..EQIRP---
AAEL010787  ASD.RR.VE.VI......I.F.E-.G--TTTLQRVTY.VL..A..M.DM.----..PQIRK.LEQVRP---
CPIJ009445  ASD.RR.VE.VI......I.F.E-SG--TTTLQRVTY.VL..A..M.DM.----..PQIRK.LEQVRP---
AGAP003663  ARRSAR----SSI.S..IVRF.C-G.--PPGPRRCSVWRVTSSATTCRST---SVRWSCRR.TT.RS---
AAEL004978  MDNIQN.CH.L.........FVD-KQ--AVT.ERVKFVVL..A..M.DM.---.MPSVEKMMNHETMRP-
CPIJ009286  IDNVNN.CH.L.........FVD-RQ--AVT.DRVKFVVL..A..M.DM.---.MPAVEKMMNHETM.S-
AGAP008578  LQLMRG.CHVL.........FID-RG--YVT.E.VNFVVL..A..M.DM.---.LPSIEKVMGHATMPE-
CPIJ014361  MRE.DR.CHLV.......E.MIM-RG--KVGLD.IRF.VL..A..M.DM.----..PQIRR.VEESKMPQ-
AGAP003047  MRD.ER.CHL........E.MIG-RG--KVGLD.IRF.VL..A..M.DM.----..PQIRR.VEESRMPV-
AAEL010402  IN.VKG.VE..I......N.LVA-AN--VIDITSITY.VL..A..M.DM.----..PQIRKLLLDIRP---
AAEL001317  SKA.EQ.AE.V......MI.MV.MK---ATNLQRVTY.VL..A.KMFNL.----..PQVRS.CNHVRPD--
```

```
AGAP012523  SKA.EQ.AE.V......MI.MV.IK---ATNLQRVTY.VL..A.KMFNM.---..PQVRS.CNHIRPD--
CPIJ019196  SKA.EQ.AE.V......MI.MI.MK---ATNLRRVTY.VL..A.KMFNM.---..KQYT-----------
AAEL013985  IAE.KR.AE...C....MI.M.AANSGRVTNLRRVTYVVL..A..MFDL.---..PQVMR..DNIRPD--
CPIJ005545  IAE.KR.AE...C....MI.M.AANSGRVTNLRRVTYVVL..A..MFDM.---..PQVMR..DNIRPD--
AGAP010656  IAE.KR.AE...C....MI.M.AANSGRVTNLR-----------------------RVMR..DNVRPD--
CPIJ014038  GLESR.HYD.L.T..N.VCFLAGHN.P-LIDLS.I.FVVV..A.KLF.ESRNS.R.QLDT.MAACTNP--
dmeRm62     MRD.QR.CE.VI......I.F.S-AG--STNL.RCTY.VL..A..M.DM.---..PQIRK.V.QIRP---

CPIJ010448  -----------------------RQTMLFSATQTSRTEELGKLALKSEPIYVGVDDNKTEATVSGLEQGY
AGAP008601  -----------------------..........S..L.....................K....T......
CPIJ007987  -----------------------.........M.DQVKD.AAVS.SKP---.K.FV.NNQTVAFN.R.EF
AAEL010317  -----------------------.........M.DQVKD.AAVS.TKP---.K.FV.NNQTVAFN.R.EF
AGAP011084  -----------------------.........M.EEVKD.AAVS..KP---.KIFV.NNQTVAFN.R.EF
AAEL013950  -----------------------.R.F.....M.KKVKK.ERAS..DP---.K.EVSSKYQ..EK.L.Y.
CPIJ008842  -----------------------.R.F.....M.KKVKK.ERAS..DP---.K.EVSSKYQ..EK.L.Y.
AGAP009808  -----------------------.R.F.....M.KKVKK.ERAS.RDP---.K.EVSSKYQ..EK.L.Y.
AAEL008500  -----------------------..IL.....FPLSVKNFMEKH.REP---YEINL-ME.L.LK.VT.Y.
AGAP009135  -----------------------..IL.....FPLSVKNFMEKH.RDP---YEINL-ME.L.LK.VT.Y.
CPIJ000753  -----------------------..IL.....FPLSVKNFMEKH.REP---YEINL-ME.L.LK.VT.Y.
AAEL013359  -----------------------V.VI.L...MPAEVL.VSTHFMRDP---IKILVK.E.L.LE.IK.F.
AGAP009863  -----------------------V.VI.L...MPADVL.VSQHFMRNP---.KILVK.E.L.LE.IR.F.
AAEL014414  -----------------------T.VC.I...LPHEIL.MTSKFMTDP---IRILVKRD.L.LE.IK.FF
AGAP003089  -----------------------T.VV.I...LPHEIL.MTSKFMTDP---IRILVKRD.L.LE.IK.FF
AAEL009285  -----------------------..MV.....LPKLMVDFA.AG.SDP---TLIRLDVESKIPEA.DLKF
CPIJ008599  -----------------------..MV.....LPKVMV.FA.AG.SNP---TLIRLDVESKIPET.DLRF
AGAP007511  -----------------------..MV.....LPKLMVDFATAG.CQP---.LIRLDVESKIPDT.DLK.
AAEL011744  -----------------------...L.....MPKKIQNFA.S..VKP---.TINVGRAG.ASMNVT.DV
CPIJ015074  -----------------------...L.....MPKKIQNFA.S..VKP---.TINVGRAG.ASMNVT.DV
AGAP004711  -----------------------...L.....MPKKIQNFA.S..VKP---.TINVGRAG.ASMNVT.DV
CPIJ000951  KPDTEEAEDATKLMENFNTKKKY...VM.T..MPPAV.R.ARTY.RRP---AT.YIGSVGKPTERT..IV
CPIJ006204  KPDTEEAEDATKLMENFNTKKKY...VM.T..MPPAV.R.ARTY.RRP---AT.YIGSVGKPTERT..IV
AAEL001769  ----------------------D..VLMW...WPKEIRK.AEEF.REY---IQINIGSLNLA--AN.NIM
CPIJ016569  ----------------------D..VLMW...WPKEIRK.AEEF.RDY---IQINIGSLNLA--AN.NIL
AGAP004912  ----------------------D..VLMW...WPKEIRK.AEEF.RDY---IQINIGSLNLA--AN.NIL
AAEL002083  ----------------------D..VLMW...WPKEVRN.AEEF.NDY---IQINIGSLNLS--ANHNIL
CPIJ014935  ----------------------D..VLMW...WPKEVRN.AEEF.NDY---IQINIGSLNLS--ANHNIL
AGAP005652  ----------------------D..VLMW...WPKEVRN.AEEF.ADY---IQINIGSLNLS--ANHNIL
AAEL008738  ----------------------D..VLMW...WPKEVQA.AEDF.HDY---IQINIGSLNLS--ANHNIH
CPIJ012512  ----------------------D..VLMW...WPKEVQA.AEDF.HDY---IQINVGSLNLS--ANHNIH
AGAP012045  ----------------------D..VLMW...WPKEVQT.AEDF.RDY---IQINIGSLSLA--ANHNIH
AAEL002351  ----------------------DH..LMW...WPDAVSR.V.DY..DY---IQINVGSLKLA--ANHNIL
```

```
CPIJ003935  ----------------------DH..LMW...WPDAVAR.V.DY..DY---IQINVGSLKLA--ANHNIL
AGAP005351  ----------------------D...LMW...WPDIVAR.V.DY..DY---AQINVGSLKLA--ANHNIL
AAEL010787  ----------------------D..ILMW...WPKEVQR.ARDF.GDY---.QINVGSL.LS--ANHNIT
CPIJ009445  ----------------------D..ILMW...WPKEVQR.ARDY.GEY---.QINVGSL.LS--ANHNIT
AGAP003663  ----------------------TCALLPRRTKTQ.KCTQPAGANGP.R---LELSPGTMFFLSGSNANVF
AAEL004978  --------------------KEE...LM....FPAEIQ..AGQF.NNY---IF.AVGIVGGA--STD-VE
CPIJ009286  --------------------KEE...LM....FPGQIQ..AGQF.NNY---IF.AVGIVGGA--SSD-VE
AGAP008578  --------------------KQQ...LM....FPAEIQ..AGKF.HNY---IC.FVGIVGGA--CAD-VE
CPIJ014361  --------------------TGE...LM....FPKAIQ..ASDF.HNY---IFLAVGRVGS.--SVN-IT
AGAP003047  --------------------TGE...LM....FPKAIQ..ASDF.YRY---IFLAVGRVGS.--SVN-IT
AAEL010402  ----------------------D...IMT...WPPGVRR.AQSYMSNP---.Q.YVGTLDLA--ATHTVT
AAEL001317  -----------------------.........FKK.I.R.ARDV.TDP---.RIMHGDLGEANEDIT.HV
AGAP012523  -----------------------...L.....FKK.V.K.ARDV.TDP---.RIIHGDLGEAN.DVT.RI
CPIJ019196  ------------------------------------VKNF..VSFVQ.---EKWLSGSARR.EP.AS---
AAEL013985  -----------------------...VM....FPRQM.A.ARRI..KP---IEIQIGGRSVVCKDV..HV
CPIJ005545  -----------------------...VM....FPRQM.A.ARRI..KP---.EIQVGGRSVVCKEV..HV
AGAP010656  -----------------------...VM....FPRQM.A.ARRI..KP---IE.QVGGRSVVCKDV..HV
CPIJ014038  ----------------------CKVVAF....V.KEVSAWARDHMPTR---.RFSVGAVNTATDLVD.EL
dmeRm62     ----------------------D...LMW...WPKEVKQ.AEDF.GNY---IQINIGSL.LS--ANHNIR

CPIJ010448  IVCP---SEKRLLVLFTFLKKNR--------KKKVMVFFSSCLSVKFHHELFNYIDLPVNSIHGKQKQAK
AGAP008601  ....---..R.............--------............................M........S.
CPIJ007987  .RIRESRDAD.EPI.AALICRTF--------HDHC...VQTKKTAHRLRI.LGLLGVKAGEL..DLT.SQ
AAEL010317  .RIREGREAD.EPL.AALICRTF--------HDHC...VQTKKTAHRLRI.LGLLGVKSGEL..DLT.SQ
AGAP011084  .RIREGREAD.EAI.AALVCRTF--------HDHC...VQTKRTAHRLRI.LGLLGVKTGEL..DLT..Q
AAEL013950  .FI.---AKYKDVY.VHI.NELA--------GNSF.I.C.T.NNTVRTALMLRALG.AAVPL..QMS.N.
CPIJ008842  VFI.---AKFKDVY.VHI.NELA--------GNSF.I.C.T.NNTVRTALMLRALG.AAVPL..QMS.N.
AGAP009808  LFI.---ARYKNVY.VHV.NELA--------GNSF.I.C.T.NNTVRTALMLRALG.AAVPL..QMT.N.
AAEL008500  AFVQ---ERQKVHC.N.LFS.LQ--------INQSII.CN.TQR.ELLAKKITELGYCCYY..A.MQ..H
AGAP009135  AFVQ---ERQKVHC.N.LFS.LQ--------INQSII.CN.TQR.ELLAKKITELGYCCYY..ARMQ..H
CPIJ000753  AFVQ---ERQKVHC.N.LFS.LQ--------INQSII.CN.TQR.ELLAKKITELGYCCYY..A.MQ..H
AAEL013359  .DVKQ--ENWK.GT.IDLYDTLS--------ITQAVI.CNTRRK.DQLTADMTSQSFT.S.M..DMD.RD
AGAP009863  VDVKY--EDWKIGT.CDLYDTLS--------ITQAVI.CNTRRK.DQLT.QMTEKTFT.SAM..DME.RD
AAEL014414  VAVER--E.WKFDT.CDLYDTLT--------ITQAVI.CNTKRK.DWLT.KMREANFT.S.M..DMP.KE
AGAP003089  VAVER--E.WKFDT.CDLYDTLT--------ITQAVI.CNTRRK.DWLT.KMREANFT.S.M..DMP.KE
AAEL009285  VY.RP--D.RYATL.ILLREVIPR-------SAQTVL.AGTQHH.ELISLILTRAGI.NTYVFSSLDASA
CPIJ008599  .Y.RP--D.RYATL.VLIREVIPK-------NAQIV..AGTQHH.ELISL.LTKSGI.CTHVYSGLDASA
AGAP007511  .Y.RP--A.RYATL.VLLREVIPS-------TAQTVI.AGTQHH.ELISLMLTKAGV.NSHVYSGLDASA
AAEL011744  EYVKQ------EAKVVYL.DCLQK------TPPP.LI.AEKKQD.DAI..YLLLKGVEAVA...GKD.EE
CPIJ015074  EYVKQ------EAKVVYL.DCLQK------TPPP.LI.AEKKQD.DAI..YLLMKGVEAVA...GKD.EE
AGAP004711  EYVKQ------EAKVVYL.ECLQK------TPPP.LI.AEKKQD.DAI..YLLLKGVEAVA...GKD.EE
```

```
CPIJ000951  HIVTE------NEKRKKLMEILSRG-----VEPPCII.VNQKKGADVLAKGLEKLGYNACTL..GKG.EQ
CPIJ006204  HIVTE------NEKRKKLMEILSRG-----VEPPCII.VNQKKGADVLAKGLEKLGYNACTL..GKG.EQ
AAEL001769  QIIECCEEYEKETR..KL.TELSQ--QGDSK---SII.VETKRK.DQITNVIKRNGWRCDG...DKT.KD
CPIJ016569  QIIECCQEYEKESR..KL.AEIGK--QGDNK---AI..VETKRK.DQIAGIIKRNGWRADG...DKT.KD
AGAP004912  QIIDCCEEYEKENR..KL.EQISS--QNDGK---TII.VETKRK.DKIVNVIRRQGWRADG...DKS.KD
AAEL002083  QIVDVCEDYEKDQK.MKL.TEISA--ENETK---TII.VETKRR.DDITRNI.RNGWRAV....DKS.QE
CPIJ014935  QIVDVCEDYEKDQK.MKL.TEISA--EAETK---TI..VETKRR.DDITRSICRNGWRAV....DKS.QE
AGAP005652  QIVDVCEDYEKDQK.MKL.TEISA--EPDTK---TII.VETKRR.DDITRIV.RNGWRAVA...DKS.QE
AAEL008738  QIVDVCEEGEKEGK.LSL..EISS--DVNSK---III.VETKKK.EDLLKNIVRDGYGAT....DKS.SE
CPIJ012512  QIVDICEENEKEGK.LSL..EIAS--DVNNK---III.VETKKK.EDLLKNIVRDGYGAT....DKS.SE
AGAP012045  QIVDVCEENEKEGK.LKL..EIATS-DATNK---III.VETKKK.DDLLKNIVRDGYGAT....DKS.TE
AAEL002351  QIIDVCQEHEKEAK.SIL.REIMA--EKECK---TII.IETKKR.DDITRKVLRDGW.AMC...DKS.RE
CPIJ003935  QIIDVCQEFEKEAK.SIL.REIMA--EKECK---TII.IETKKR.DDITRKVTRDGW.AMC...DKT.RD
AGAP005351  QIIDVCQEYEKESK.SIL.REIMA--EKECK---TII.IETKKR.DDITRKVKRDGW.ARC...DKS.NE
AAEL010787  QYVKVIEEHEKNEQ.GKL.DNLSAR-GPAGK---ILI.STTKRKCDQITSYLRRYGQDAVGM..DKS.QE
CPIJ009445  QYVKVIEEHEKNEQ.GKL.DNLQSR-GNPGK---ILI.TTTKRKCDQISTYLRRFGQDSVGM..DKS.QE
AGAP003663  LFVFSSPLFSLST..GKL.EELYHE-GNPGK---ILI.TTTKRQCDRISMQIKRYGYDSV.M..DKS.QE
AAEL004978  QTIHQVSKFQKRKK.EEL.E--ADDPTG------TL..VETKRNADYLAS.LSETKF.TT....DRL.RE
CPIJ009286  QNIYEVTKFQKRKK.EEI.E--SNDPKG------TL..VETKRNADYLAS.LSETKF.TT....DRL.RE
AGAP008578  QTIHLVEKF.KRKK.EEI.N--GGNPKG------TL..VETKRNADYLAS.MSETQF.TT....DRL.RE
CPIJ014361  QSIFWVEENDKRSH.LDL.SNIKDQNDGDEKDCLTLI.VETKK.ADALEDFLYNYNH..T....DRT.KE
AGAP003047  QTIFWVEENIKRSH.LDL.SNITKQNDGDDENCLTLI.VETKKAADSLE.FLYNHNF..T....DRT..E
AAEL010402  QQIEVIDE.DKYMRVMN.VTN-MGPSDK------.II.CGRKTRADDLSSE.VLSGINCT.L..DRE..D
AAEL001317  ..MNNPAHKWNW.LAKMVELLSEGT---------.LI.VTKKADAEQVANNLRLKEYDPVLL..DMD..D
AGAP012523  .LL.TVQ.KWNW.LTNLVKMLSEGS---------.LI.VTKKADAEETANNLRLK.ND.VLL..DMD.SE
CPIJ019196  --------------------LLEEV---------WLAYLWKR----------------------------
AAEL013985  V.LEEDAKFFK..E.LG-.YQELGS---------II..VDKQENADILLKDLMKASY.CL.L..GID.FD
CPIJ005545  V.LEEDAKFFK..E.LG-.YQELGS---------II..VDKQENADILLKDLMKASY.CM.L..GID.FD
AGAP010656  V.LDDEAKFFK..E.LG-IYQEQGS---------II..VDKQENADSLLKDLMKASY.CM.L..GID.FD
CPIJ014038  LFVGN-----ESGK.LA.REAVHKG-----LTPP.L..VQ.KDRAQQLFTELL.DG.N.DV..ADRS.RE
dmeRm62     Q.VDVCDEFSKEEK.K.L.SDIYDTSESPGK---III.VETKRR.DNLVRFIRSFGVRCGA...DKS.SE

CPIJ010448  RTSVFFQFCNAESGILLCTDVAARGLDIPAVDWIVQYDPPNDTKEYIHRVGRTARGDNLCGHALLLLRPE
AGAP008601  ............T.........................................ED.......M....
CPIJ007987  .LESLK..KDEQIDV.VA..........SG.KTVINFVM.ATMEH..........AGKAGVSVS.AGEL.
AAEL010317  .LESLK..KDEQVDV.IA..........SG.KTVINFVM.ITMEH..........AGKAGVSVS.AGEL.
AGAP011084  .LESLKE.KDEQVD..IA..........ST.KTVINFVM.ATLEH..........AGKAGVSVS.AGEQ.
AAEL013950  .LAALNK.KGKNRQ..IS....S......H..VVLNF.I.THS.D..........AGRAGKAVTFVTQYD
CPIJ008842  .LASLNK.KGKDRQ..IS....S......H..VVLNF.I.THS.D..........AGRAGKAVTFVTQYD
AGAP009808  .LAALNK.KSQARQ..IS....S......H..VVLNL.I.MHS.D..........AGRAGQAITFVTQYD
AAEL008500  .NR..HD.RSGLCRN.V.S.LFT..I.VQ..NVVINF.F.KMAET.L..I..SG.FGH.GIAIN.ITYED
```

```
AGAP009135  .NR..HD.RSGLCRN.V.S.LFT..I.VQ..NVVINF.F.KMAET.L..I..SG.FGH.GIAIN.ITYED
CPIJ000753  .NR..HD.RSGLCRN.V.S.LFT..I.VQ..NVVINF.F.KMAET.L..I..SG.FGH.----------D
AAEL013359  .DLIMK..RTGS.RV.IT..LL...I.VQQ.SLVIN..L.TLREN....I..GG.FGRKGVAINFVTDVD
AGAP009863  .DLIMK..RTGS.RV.IT..LL...I.VQQ.SLVIN..L.TLREN....I..GG.FGRKGVAINFVTEQD
AAEL014414  .DEIMKE.RSGQ.RV.IT...W...I.VQQ.SLVIN..L..NREL....I..SG.FGRKGVAINFVKSDD
AGAP003089  .DEIMKE.RSGQ.RV.IT...W...I.VQQ.SLVIN..L..NREL....I..SG.FGRKGVAINFVKSDD
AAEL009285  .KINTAK.TMKKVNV.VV..I........SL.YV.NVHF.GKP.LFV.....C..AGRSGTAYNIFSNDD
CPIJ008599  .KINTAK.TMKKVNV.VV..I........TL.YV.NVHF.GKP.LF......C..AGRSGTAYNIFSNDD
AGAP007511  .KINTAK.THRKVNV.VV..I........TL.FVINLHF.GKP.LF......C..AGRSGMAYTIFSNDD
AAEL011744  .YRSVEG.R.Q.KDV.VA....SK...F.D.QHVIN..M.D.IEN.V..I...G.SGSKGLATTFINKAT
CPIJ015074  .YRSVEG.RTQ.KDV.VA....SK...F.D.QHVIN..M.D.IEN.V..I...G.SGSKGLATTFINKAT
AGAP004711  .YRSVES.R.Q.KDV.VA....SK...F.D.QHVIN..M.D.IEN.V..I...G.SGSKGLATTFINKAT
CPIJ000951  .EYALASLK.GSKD..VA....G..I..KD.SLVIN..MAKTIED.T..I...G.AGKTGCAISFCTKD-
CPIJ006204  .EYALASLK.GSKD..VA....G..I..KD.SLVIN..MAKTIED.T..I...G.AGKTGCAISFCTKD-
AAEL001769  .DY.LNT.RRLR....VA....S....VDD.KYVINF.F..N.ED....I...G.ST.KGTSYTFFT-.A
CPIJ016569  .DY.LNT.RRMNN...VA....S....VDD.KYVINF.F..N.ED....I...G.ST.KGTAYTFFT-.A
AGAP004912  .DY.LNT.RRSTN...VA....S....VDD.KFVINF.F..N.ED.V..I...G.ST.KGTSYTFFT-.A
AAEL002083  .DY.LNA.R.GRQ...VA.........VED.KFVIN..Y.SNSED.V..I...G.SN.TGTAYT.FT-NS
CPIJ014935  .DY.LNA.R.GRQ...VA.........VED.KFVIN..Y.SNSED.V..I...G.SN.TGTAYT.FT-NS
AGAP005652  .DY.LST.R.GRQ...VA.........VED.KFVIN..Y.SNSED.V..I...G.SN.TGTAYT.FT-NS
AAEL008738  .DY.LQD.RHGK.T..VA.........VED.KYVINF.Y..SSED....I...G.CSSYGTAYTFFT-.G
CPIJ012512  .DY.LQD.RHGK.T..VA.........VED.KYVINF.Y..SSED....I...G.CSSFGTAYTFFT-.G
AGAP012045  .DY.LQD.RHGK.T..VA.........VED.KYVINF.Y..SSED....I...G.CSQYGTAYTFFT-.N
AAEL002351  .EYTLNS.RSGKNP..IA.........VDD.KFVINF.Y.TTSED....I...G.SN.TGTAYTFFT-.D
CPIJ003935  .DNTLKS.RSGKTP..IA........EAHNKTSALR----------------------------------
AGAP005351  .DATLN--------------------YVDD.KFVINF.F.TTSED....I...G.CN.TGTAYTFFT-.N
AAEL010787  .ERALNR.R.SN.C..VA.........VDGIKVVIN..Y.QQ.ED.V..I...G.SNATGEAYTFFT-SN
CPIJ009445  .ERALNR.R.SN.C..VA........EAG-----------------TF.D.DSQKTVRVAPQWPVDD-TS
AGAP003663  .ERALGR.R.SS.C..VA.........VDGIKVVIN..Y.QQ.ED.V..I...G.SNATGVAYTFFT-MA
AAEL004978  .EEALRD.KSGKMF..IA.S........KN.AHV.N..L.KSIDD.V..I...G.VG.KGKATSFYDMEA
CPIJ009286  .EEALRD.KSGKMY..IA.S........RN.AHVIN..L.KGIDD.V..I...G.VG.KGRATSFFDMEN
AGAP008578  .EMALYD.KSGRMDV.IA.S........KN.NHV.N..L.KSIDD.V..I...G.VG.KGRATSFYDPEA
CPIJ014361  .EEALKF.RSGRCPV.VA.A.........N.KHVINF.L.AEVE..V..I...G.MG..GTATSFFN-DK
AGAP003047  .EEALRL.RCGRCP..VA.A.........N.KQVINF.L.AEVE..V..I...G.MG..GTATSFFN-EK
AAEL010402  .EQALEDIKSGDVRV.IA....S.....EDISHV.N..F.RNIE..V......G.AGRSGVSLSFFT-RG
AAEL001317  .NI.ITR.RKR.VE.MVA...........HIKNVIN..IAR.IDTHT......G.AGEKGTAYT.VVDKD
AGAP012523  .NF.ITR.KRKDVD.MVA...........HIRTV.N..IAR.IDTHT..I...G.AGEKGTAYT.ITDKD
CPIJ019196  --------RRR------------------------------------.TEPSTP.GIKKAATS.SCSGG
AAEL013985  .D.TIID.KQGRVKL.IA.S.......VKQLILV.N..C..HYED.V..C...G.AG.KGFAWTF.THEQ
CPIJ005545  .D.TIID.KQGRVKL.IA.S.......VKQLILV.N..C..HYED.V..C...G.AG.KGFAWTF.THEQ
AGAP010656  .D---------------------------------------------------------DYGFAWTF.TPEQ
```

```
CPIJ014038  .DN.VRS.REGKIW..I..ELMS..I.FKG.NLV.N..F.PS.IS.V..I...G.AGRRGRAVTYFTKDD
dmeRm62     .DF.LRE.RSGK.N..VA.........VDGIKYVINF.Y.QNSED....I...G.SNTKGTSFAFFT-KN

CPIJ010448  EVAFLKYLKQAKVPLN-----------------------------------EFEFSWNKIADIQLQLETL
AGAP008601  ..G.............-----------------------------------......S..........N.
CPIJ007987  RKIVKDII.N.VN.VKNR----------------------------IIPNEIV.KYRK.VQALEEEI.NV
AAEL010317  RKIVKDII.N.VS.VKNR----------------------------IIPTEIVDKYRK.VLALEEEI.KV
AGAP011084  RKIVKEIV.N.VSSVKNR----------------------------IIPLDII.KYR..V.ALEPEIDRV
AAEL013950  ----------------------------------------------------V.LYQRIEHLLGKK.PQF
CPIJ008842  ----------------------------------------------------V.LYQRIEHLLGKK.PEF
AGAP009808  ----------------------------------------------------V.LYQRIEHLLGKK.PEY
AAEL008500  RFDLHRIE.ELG-----------------------------------TEIKPIPKVIDPALYVPRPEDQN
AGAP009135  RFDLHRIE.ELG-----------------------------------TEIKPIPKVIDPALYVPRPDDPN
CPIJ000753  LHSIHANNSHRH-----------------------------------LEALALPSFGDP--FLRWLT.RE
AAEL013359  RRVLQDIE.HYN-----------------------------------TKIE.MPANLADML---------
AGAP009863  KRVLADIE.HYN-----------------------------------TTIE.MPANLADMI---------
AAEL014414  IRILRDIEQYYS-----------------------------------TQID.MPMNVADLI---------
AGAP003089  IRILRDIEQYYS-----------------------------------TQID.MPMNVADLI---------
AAEL009285  IAHMIDLHMFLTR..VLSDARCIGVAPPDMVEAEHQLVLDYVKHIDLATAFRISNNAY.QYIVTRPAASA
CPIJ008599  IAHMIDLHMFLTR..DVTDSRNMGRAPPEAVESEHQIVLERIKHIDLATAFRVSNNAY.QYIVTRPAASA
AGAP007511  VAHLIDLHMFLNR..DVADRKTMGIVPPDMQETEHLLVQEYVRHVDLATAYRVSNNAY.QYIVTRPAASA
AAEL011744  .QFV.LD..HLL----------------------------------------I.AKQKVPPFLGELCSET
CPIJ015074  .QYV.LD..HLL----------------------------------------L.AKQKVPPFLAELCSET
AGAP004711  .QFV.LD..HLL----------------------------------------I.AQQKVPPFLGELCSET
CPIJ000951  DSHLFYD...II----------------------------------------VASPVSSCPPELMNHPDA
CPIJ006204  DSHLFYD...II----------------------------------------VASPVSSCPPELMNHPDA
AAEL001769  NG.KAGD.IGVLR----------------------------------------.ANQFVNPELEQYAR--
CPIJ016569  NSSKAND.I.VLK----------------------------------------TANQYVNPEL.EYAR--
AGAP004912  NSSKAPD.ITVLQ----------------------------------------DANQYINPELHEYARGS
AAEL002083  NANKAGD.INVLR----------------------------------------.ANQVINPKLAEMAKPG
CPIJ014935  NANKAGD.INVLR----------------------------------------.ANQVINPKLVEMTKHG
AGAP005652  NANKAND.INVLR----------------------------------------.ANQVINPRLVELAKPS
AAEL008738  NGRQARE.LSVLE----------------------------------------.AGQQPT.QLIDLAKQA
CPIJ012512  NGRQARE.LSVLE----------------------------------------.AGQQPTPELISMAKSM
AGAP012045  NGRQARE.LSVLE----------------------------------------.AGQQPTVELVEMAKQA
AAEL002351  NAGRARE.IDVLK----------------------------------------.AKQVINPKLLDMTTMR
CPIJ003935  ----------------------------------------------------------------------
AGAP005351  NASKARD.IDVLK----------------------------------------.AKQVINPKLVELASMK
AAEL010787  .RKMA.E.VAILE----------------------------------------.AKQDVPPELLKWRHMG
CPIJ009445  LF.AAAAVAARLP----------------------------------------CAEH-------------
AGAP003663  .RKQARE.VNILQ----------------------------------------.AKQ-------------
```

```
AAEL004978  DA.IAPD.VKILT----------------------------------------QAGQQVPDFLEGLST--
CPIJ009286  DS.IAGD.VKILT----------------------------------------QAGQQVPDFL.GMSG--
AGAP008578  DR.MASD.VKILT----------------------------------------QAGQSVPDFLKDAG---
CPIJ014361  NRNVATG.VRLLT----------------------------------------.TQQEIPGFLEDMTTDR
AGAP003047  NRNVANG.VRLLA----------------------------------------.TGQEIPGFLEEMTNSR
AAEL010402  DW.VASD.IKILE----------------------------------------.ADQEVPEE.RQMA.RF
AAEL001317  KEFAGHLVRNLEG-----------------------------------------ANQEVPEELMKLAMQS
AGAP012523  KEFSGHLVRNLEG-----------------------------------------ANQDVPE.LMKLAMQS
CPIJ019196  RISGRIFAEELE----------------------------------------------------------
AAEL013985  GRYSGDIIRALEL-----------------------------------------SGGTVPD.LRSLWD.Y
CPIJ005545  GRYSGDIIRALEL-----------------------------------------SGGTIPD.LRQLW..Y
AGAP010656  GRYSGDIIRALEL-----------------------------------------SGGTVPE.LRNLWD.Y
CPIJ014038  TTNLRGIAQLIRK-----------------------------------------SGGTVPEYMLKLKQSS
dmeRm62     NAKQA.A.VDVLR----------------------------------------.ANQEINPALENLARNS

CPIJ010448  LAKN-------------------------------------YFLNQSGKLAFKSYVRAYEGHHMKDVFNV
AGAP008601  M...-------------------------------------............T...............I
CPIJ007987  .QEEHAEKLLRQTEQQLSKTENKLKGIS-------NGPSREW.Q.SHERHQE.ERLSIKTDEEKAEKKKQ
AAEL010317  .CEERAEKMLLQTEQQLNRTERKLKGVV-------SGPSREW.QTHHERKEE.ERLSVKN.DE-NEKRKK
AGAP011084  ..EERAEKLLRQTEQQLTSAERKLLGNAGKAAKQDVPPPREW.QT.HERREE.NRLAG---EEQGEKKKA
AAEL013950  KCEE------------------------------------DEVMALQERVGEAQRTARL.LKDID.RKGS
CPIJ008842  KCEE------------------------------------DEVMALQERVGEAQRTARL.LKDIEERKGM
AGAP009808  KCEQ------------------------------------DEVMALQERV.EAHRTARI.QRDIEERKAS
AAEL008500  STQEEQNISK------------------------------------------------------------
AGAP009135  STQEEQNVSK------------------------------------------------------------
CPIJ000753  VLQLYRQAPP------------------------------------------------------------
AAEL013359  ----------------------------------------------------------------------
AGAP009863  ----------------------------------------------------------------------
AAEL014414  ----------------------------------------------------------------------
AGAP003089  ----------------------------------------------------------------------
AAEL009285  S.NKKAKQFKIGELQVLEDFGKVMEKLDE--------------DKKAD--------KQKKADAAQK--KQ
CPIJ008599  SSNKKAKRFKISELKVLDEFAAAMEKENE--------------AKKKD..LG.KAKKQKKALTA.QEEKK
AGAP007511  ASNKRAKQFKIDELGVLEDFQQEKAEPEAGARGKRWKKGKPIS.KKKD.KEEEKKEQQEK.KTESSN-SK
AAEL011744  EKYADLG------------------------------------DGC.YCGGLGHRITECPKLEAIQSKQA
CPIJ015074  EQYADLG------------------------------------DGC.YCGGLGHRITECPKLEAIQSKQA
AGAP004711  EKYADLG------------------------------------DGC.YCGGLGHRITECPKLEAVQSKQA
CPIJ000951  QH.P------------------------------------------------GTV.TKKRREEKIFA---
CPIJ006204  QH.P------------------------------------------------GTV.TKKRREEKIFA---
AAEL001769  ---SGG---RNRS---GGRGRYGGGGGGRQ--MRNDGKGRR--GDD-YRSGNGDDRGPKFARRDDYGSGN
CPIJ016569  ---FGGGGGRNRGRGGNGGGRFGGGGGRDRGSFRNDAKGRR--GDD-FGR-NGDDRGPKFARRDDYGGG-
AGAP004912  GRYRGSGGGRSRGNMSGGRGMGGRMGGGSR--FSDSRSGRP--NDGGYRNGNDGRGG.SYPRRDDFGRS-
```

```
AAEL002083  M----NRHGQRHNR-----YGNRYGGQQNRPPRDG-GYGGQ--R.DG.HRFGGMNNGNK---FGGPRGDA
CPIJ014935  M----RGGGRSRYG-----NNNRYG--QNRPPRDNNGYGGQ--R.DG.NRFG--AGGNK---FGGGGGGG
AGAP005652  M----GKGRQRYN-------NHRFGGQQNRPPRDGPYGGGP--RHDG.-RFGGQRDGGAPPKYGGGGGGM
AAEL008738  P---GGKGGRSRYN----VRGALTSGGYNR-DQNGFGGNR---MF.KKP-FENRFGGPPVNGMGPNR..N
CPIJ012512  P---GGKGGRSRYN----VRGALTSGGYGRMDQGGFGGPRGGGGF.KKP-FENRFGGPPG-GMGGNR.GS
AGAP012045  P---GGKGGRLRYSTQTGYRGSSGMGSYQR--RPPFNGGG---GFGGPPKYGGGMQGGMM.GMQN-K.GG
AAEL002351  IK---GRGKRTFISSRYPRERRSRSFDRRGVFRRSSY-----SRSR.RSRSRSRSRSRSASPRFSKRDVY
CPIJ003935  ----------------------------------------------------------------------
AGAP005351  VK---GKGNR-HMTTRYPRERRSRSRSKSRSPVRSRRPAGLSDRRR.HSRSRSRSRSPVRRSPVGGRRD.
AAEL010787  G---------------------------------------------------------------------
CPIJ009445  ----------------------------------------------------------------------
AGAP003663  ----------------------------------------------------------------------
AAEL004978  --GGGSFGG-----------GSQFGGRDIRS-RD------------------------------------
CPIJ009286  --GGGSYGG-----------PSQFGARDIRGGRD------------------------------------
AGAP008578  --GSGSYMG-----------SSQFGGKDIRD---------------------------------------
CPIJ014361  GWGSRGRGGGGGRNQRYGGQSSSFGSRDYRTQGGNRNNNTR-----------------------------
AGAP003047  SFGGNRRG----RVPRGGG-GSTFGSRDYRQQN-------------------------------------
AAEL010402  T..KEREGR----------EKSAFGRR-------------------------------------------
AAEL001317  AWFRNSRFKHNNKGKNLNVGGAGLGFRCRPVPSGPLKPS---------DG--------------------
AGAP012523  SWFRNSRFKHANKGKNLNVGGAGLGFRQRAIQRGPMGRSLDASKVLVEDGD-------------------
CPIJ019196  --------------------------------RGSIN---------------------------------
AAEL013985  K.AQEAEGKKVHTGGGFSGKGFKFDEQEAAAVNERKKLQKAALGL.DSDDDDDLEQDIDQHIESMFATKR
CPIJ005545  KTTQEEQGKKVHTGGGFSGKGFKFDEQEAAAVNERKKMQKAALGL.DSDDDEDLEQDIDQHIESMFATKR
AGAP010656  K.AQEAEGKKVHTGGGFSGKGFKFDAQEAAAVNERKKMQKAALGL.DSDDEEDLEQDIDQQIENMFATKR
CPIJ014038  KKDR------------------------------------------------------------------
dmeRm62     RY--DGGGGRSRYG---------GGGGGGR-----FGGGG----FKK.S---------------------

CPIJ010448  GNLDLLQVARNFGFTQPP----------------------------------------------------
AGAP008601  A....V...K........----------------------------------------------------
CPIJ007987  NKNKRKRGGDDSEDEFD.IKYQQEKKQKVAKKAKAEAAKTPKQLAKERALQELTKVSMVQAKLSKIKSRP
AAEL010317  NK-KRKRG-DDSDDEFD.IKYQQEKKQKVAKK-KAEVHKTPSQLAKERALQELTKVSLVQAKLAKIKNRP
AGAP011084  AK-KRKRRDDDDEEDFD.VRYHEEKR-KQQKAAAGGGGATAKQLARERALEEVRKVSMVQAKLAKIRNRP
AAEL013950  KGKKRGRGGDSDDD--------------------------------------------------------
CPIJ008842  KRKGRGKGGDSDDD--------------------------------------------------------
AGAP009808  KGGKRG-ANDSDEE--------------------------------------------------------
AAEL008500  ----------------------------------------------------------------------
AGAP009135  ----------------------------------------------------------------------
CPIJ000753  ----------------------------------------------------------------------
AAEL013359  ----------------------------------------------------------------------
AGAP009863  ----------------------------------------------------------------------
AAEL014414  ----------------------------------------------------------------------
```

```
AGAP003089  ----------------------------------------------------------------------
AAEL009285  STVNPDEFRSS.LAQMKNYRPQSTIFELNPKNNAKEVVVMAEKRNKDTIKIEKHKQKQLELELEEQ--KK
CPIJ008599  PAV.ADEFRTS.LAQMKSYRPQTTIFELNPKSNAKRMLIMNEKREKDTIKIEKHKRKLAELEEEEE--KK
AGAP007511  PTV.ADAFRND.LARMKNYRPNATIFELNPKAHARELVAMTQKREADEAKIEKHKRKLAELEQEEQEKQK
AAEL011744  S.IGRRDYLS.TAADY------------------------------------------------------
CPIJ015074  S.IGRRDYLS.TAADY------------------------------------------------------
AGAP004711  S.IGRRDYLS.TAADY------------------------------------------------------
CPIJ000951  ----------------------------------------------------------------------
CPIJ006204  ----------------------------------------------------------------------
AAEL001769  R.DYGSRNGFGGDRDG.R-GGGFGG---------------------------------------------
CPIJ016569  ---FGAKSGGGGLDSGFKSGGAFGS---------------------------------------------
AGAP004912  ----DRDHRDGGSRSGAS-YGATGA---------------------------------------------
AAEL002083  .---GDKFGSKPAQNGGY----------------------------------------------------
CPIJ014935  A---GNGYG-------------------------------------------------------------
AGAP005652  .RS.GDKYGVASRAAGDNRLSMYGV---------------------------------------------
AAEL008738  --PGPNKYGGPG.-YRSENSWNKN---------TGGYQAVPSHQTQNGTPQQSPPQQQLYEPHQQMRFHS
CPIJ012512  .PNGPNKFGGAG.GYRGADSWNKNG--------AGGYQQQQPYQAQN--PQSGGQQQQLYEPHQQMRYQP
AGAP012045  .-GMQNKFR-DN.MYRGENNWNKGPNGPAMMGGPGGYQSHSPQSPQAAAVASGPQMYEAQQAQLRAAYHP
AAEL002351  R---------------------------------------------------------------------
CPIJ003935  ----------------------------------------------------------------------
AGAP005351  R---------------------------------------------------------------------
AAEL010787  ----------------------------------------------------------------------
CPIJ009445  ----------------------------------------------------------------------
AGAP003663  ----------------------------------------------------------------------
AAEL004978  ----------------------------------------------------------------------
CPIJ009286  ----------------------------------------------------------------------
AGAP008578  ----------------------------------------------------------------------
CPIJ014361  ----------------------------------------------------------------------
AGAP003047  ----------------------------------------------------------------------
AAEL010402  ----------------------------------------------------------------------
AAEL001317  ----------------------------------------------------------------------
AGAP012523  ----------------------------------------------------------------------
CPIJ019196  ----------------------------------------------------------------------
AAEL013985  IVKEVEAPVITQS---------------------------------------------------------
CPIJ005545  IVKEVEAPVITQT---------------------------------------------------------
AGAP010656  IVKEVEPP.S.KHEQ.NN----------------------------------------------------
CPIJ014038  ----------------------------------------------------------------------
dmeRm62     ----------------------------------------------------------------------

CPIJ010448  ----------------------------------HVDFGKSYKIQNSDRRAGNRGLGHFKSLNKDKKGEM
AGAP008601  ----------------------------------Y......F.LH..E..P....M....T...E.E.KL
```

```
CPIJ007987  SRVTATEDEKEPP----KGGKQFKQKKR---SRFER.LTDVS.KGVKKL.YDATKKQKMDKFD.K.MSN.
AAEL010317  SRVTAAADDDDFG----KGKNNSRQKNKKRLSRFEK.LTDTS.KGVKKL.YDATKKQKMDKFD.G.MSN.
AGAP011084  GRVNAAEEEHGNGPADGRAGGKSNKKRSKNFSRFEH.LTDVGSKNVKKL.YDASKKQRMDK.D.R.QSG.
AAEL013950  ---------------------------------------DTEQFNGVRK.IK-----------.GM..G-
CPIJ008842  ---------------------------------------DTEQFNGARK.IK-----------.NGA.GG
AGAP009808  ---------------------------------------DTEQFNGVRK.LKPGNAKGGDRGGRGGR.GR
AAEL008500  ----------------------------------------------------------------------
AGAP009135  ----------------------------------------------------------------------
CPIJ000753  ----------------------------------------------------------------------
AAEL013359  ----------------------------------------------------------------------
AGAP009863  ----------------------------------------------------------------------
AAEL014414  ----------------------------------------------------------------------
AGAP003089  ----------------------------------------------------------------------
AAEL009285  ASAENVPDEDEYP---KKKKRKPVKDEENFISYQSK.AVEDDGYAINSFAKEANSAELSVIGDTAADQRS
CPIJ008599  AAQAVKADEE------SKDKKSAVKDDENYISYQAK.HVEEDGYAINSFAKEASNAELSVIGDTAADQRQ
AGAP007511  SKATTVEDEEEQKGAGGKRRKGPVRDEEHFIAYQAK.AIDEDGYAIDNFTRQANSAELSVVGDTAEGQR.
AAEL011744  ----------------------------------------------------------------------
CPIJ015074  ----------------------------------------------------------------------
AGAP004711  ----------------------------------------------------------------------
CPIJ000951  ----------------------------------------------------------------------
CPIJ006204  ----------------------------------------------------------------------
AAEL001769  ---------------------NKDRDGGFKSNGYSGSNDRGGYGSRDTAKG.SGFGSSSDKSGAYGTSNE
CPIJ016569  ---------------------GGGRDGGFRSGGTSG---LGGFGSSNSSSVRPLNGTSS---GGYGADRH
AGAP004912  ---------------------ARAPNG-VGTNGYGASGTPAAYGSDDRGYG.-HYGSVSQPQAHYGASG-
AAEL002083  ------------------------------QSRDQ-NRIPNGSGPP.GA.PSRFSAPSNAPPSFPSG---
CPIJ014935  ------------------------------QQRDQGNRMANGSGPP---.PSRFSAAPPSGASSYGGSNG
AGAP005652  -----------------GAGLPPQTLGGLAASTAMGAKPPGVGGGVVGQ.QSRFSGPPSSATAAAAAAAF
AAEL008738  KNTMYQNPNQYEDFSNQMVGGAPQAFNGHSGTRFYSNKPHQGTGGMQQTQL.GP.GRYNPNN-QYNGDVG
CPIJ012512  KN-MYQN--QYEDFSNQMVGGAPQAFNGHQGTRFYQGKPHQGQGGLQQTQP.GP.GRYQPNN-QYG--AD
AGAP012045  KN-IYPG--QFDDYTALFAGGATAAAPG-GGMRYYQNKAHQGGPGAMQPG-SQP.GRYNPNGGQFNMDGT
AAEL002351  ---------------------------------PVKRSVSRGSRSD.SDSRSDNRRKKNSRRVSRSVSRS
CPIJ003935  ----------------------------------------------------------------------
AGAP005351  ---------------------------------TRGSSPGRRS.SR.P..S.SGSRRRLLPPAAR.ASRS
AAEL010787  ----------------------------------------GGINRYGSGGGNRF.TFKGGRGGDFGR.GG
CPIJ009445  ----------------------------------------AGIRPH-----HLWDTLR------------
AGAP003663  ---------------------------------------------------DIPSEL-------------
AAEL004978  -----------------------------------------TGGSRV.AQPKQMEPEEDWD---------
CPIJ009286  -----------------------------------------AEGSRM.AQPSALEPDEEWN---------
AGAP008578  -----------------------------------------SYGSRV.AQPVALEPEEEWE---------
CPIJ014361  --------------DQRSGGGGGGGGGAGRSSYGGGGGSYGGGGSRD.GGYYRN.G.SSGGYGGSYNNNS
AGAP003047  ---------------------------SGRS----------GGGSRDH.PM.GG.G.SRGGYGG-----G
```

```
AAEL010402  -----------------------------------------DGGGRGGGGG.R..G.RW-----------
AAEL001317  -----------------------------------PSSSSAGGSGWKGPVTDRLTAMRDTFKAQYNAQFK
AGAP012523  -----------------------------------PSGPSPGSS--KGPATDRLSAMRETFRAHYNAQFK
CPIJ019196  -----------------------------------------------GPQPWRLCDRGDCVNE.LYRV--
AAEL013985  ----------------------VSTPAP-TTTNSDKLELAKRLASRINLQKNLGLEAKGATQQAAEAILK
CPIJ005545  ----------------------VTVPTPPVATNSDKLELAKRLASRINLQKSLGFEAKGVTQQAAEAILK
AGAP010656  -------------------PPPAPAPPVPSVTHSDKLELAKRLASKINLQKTLGTDSKGATQQAAEAILK
CPIJ014038  -----------------------------------KKLLQKAPKRAAI.TLPAFEMQERARKK.LIAQSK
dmeRm62     ---------------------------------LSNGR.FGGGGG------.GGEGR.------------

CPIJ010448  KFKHIKNRKELKKVDYK-----------------------------------------------------
AGAP008601  .I...TD..Q....K.E-----------------------------------------------------
CPIJ007987  .MSAD.F.NKKGLNKQNKGKSFGP---RKGKR--------------------------------------
AAEL010317  .MSAD.F.NKKGLNKSNKGKSFGPKQSRKGKH--------------------------------------
AGAP011084  .MSTE.L.NKHGLNKQTKGKSFGQKAPRRKPTDG------------------------------------
AAEL013950  ---KG.FK.-------------------------------------------------------------
CPIJ008842  GKGKG.FK.-------------------------------------------------------------
AGAP009808  GGRGG.RGRRN-----------------------------------------------------------
AAEL008500  ----------------------------------------------------------------------
AGAP009135  ----------------------------------------------------------------------
CPIJ000753  ----------------------------------------------------------------------
AAEL013359  ----------------------------------------------------------------------
AGAP009863  ----------------------------------------------------------------------
AAEL014414  ----------------------------------------------------------------------
AGAP003089  ----------------------------------------------------------------------
AAEL009285  HQRLQ.WDRKK..MVNVVNPKAGKIRTEHGVWIAASYKTGRYDKWKERTKVDE---QMARQRQDSDASDG
CPIJ008599  HQRLQ.WDRKK..MVNVVNPKAGKIRTEHGVWIAASYKTGRYDKWKERTKVDE---QERARQNDSDEEDS
AGAP007511  HRQLQ.WDRKK..MVNVENPKAGKIRTEHGVWIAASYKTGRYDKWKERTKLDEKLLAEQAQQSDEEDGDG
AAEL011744  ----------------------------------------------------------------------
CPIJ015074  ----------------------------------------------------------------------
AGAP004711  ----------------------------------------------------------------------
CPIJ000951  ----------------------------------------------------------------------
CPIJ006204  ----------------------------------------------------------------------
AAEL001769  SRSSGYTSRQNGT---NAGTFSGGSVPLPNFSKPPPSFAAAPPTGGFTAPPPSRRPEERQRPPLASTVGS
CPIJ016569  VPSIPSAAS---------------------FSKPPPSFGSGPPSG-FSAPPPSRGTDLRSRPPLASTVGS
AGAP004912  YPAQGSVAR-----------------------RPESDRSRVPASAAIGLPVTPAMPSAIAYAHPGMALSA
AAEL002083  -ATGGN--SYN.G---------------AYQNGSAASGNSAPTSNGYG----SYKPYTTAPPTQSATGAS
CPIJ014935  SAGGGY--GAKSG---------------GYQNGGGASNGSAAPANGYG----SYKPYSSAPPSQSAAPAT
AGAP005652  ATSYHQQQSKYPG---------------AGSVGGAGLAGAHPSAAAYGASAASYKPQSAPGSDLYGGSGG
AAEL008738  PKPAGGRPPYQP.QQQFNNSYAAAGTANPYPAQLAAAAAAAAAAVGGGQTIPAAGFA--TGFDPLAGVQG
CPIJ012512  PKSAGGRPPYQP.QQQYNNSYAAAGTAAAYPAQLAAAAAAAAAAVGGGQTIPAAGFA--TGFDPLAGVQG
```

```
AGAP012045  PKPQNGRGTYPP.--QYNNSYAAG---PANPSHQYPQLAAAAAAAAAGQTIPTGAPAGFTGFDPTGGLQ-
AAEL002351  PAP-RGAATNKSG----------------------SAGDKKRNGKSRSPTTRRHRSRSRSRSRSRS----
CPIJ003935  ----------------------------------------------------------------------
AGAP005351  PPPSRSPVASSRR----------------------VNGREQKNGRSLSRERERDRDRERERDRDRGDRDR
AAEL010787  DRYGGGD.GAKRS----------------------------------------------YGGSSSYGGST
CPIJ009445  ----------------------------------------------------------------------
AGAP003663  ----------------------------------------------------------------------
AAEL004978  ----------------------------------------------------------------------
CPIJ009286  ----------------------------------------------------------------------
AGAP008578  ----------------------------------------------------------------------
CPIJ014361  GGGGGGGGGHDWWNE-------------------------------------------------------
AGAP003047  GGGGGGYGGK------------------------------------------------------------
AAEL010402  ----------------------------------------------------------------------
AAEL001317  ASSDRTWENT.PEGGVFAKPNLSGFVPASSGEAGDQPQRNDEHDQSYTGEED-RPRKKKSRWN-------
AGAP012523  ASSDRTWENTVPEGGVFIKP------------AQPELESSSKSDSNLTSKIDTMPRKKKSRWN-------
CPIJ019196  ----------------------------------------------------------------------
AAEL013985  GGTTQQLITAKTVAEQLAAKLNTKLNYQPKDEDESPVESNETVFRKYEEELEINDFPQQARWKVTSKEAL
CPIJ005545  GSSTQQLITAKTVAEQLAAKLNTKLNYQPKDEEEAPVETNEQVFRKYEEELEINDFPQQARWKVTSKEAL
AGAP010656  GANTQQLIAAKTVAEQLAAKLNNKLNYQPKDEEEPVVETNEQVFRKYEEELEINDFPQQARWKVTSKEAL
CPIJ014038  AK.EG.SK.-------------------------------------------------------------
dmeRm62     -----------------------------------------------------------SRFD-------

CPIJ010448  ----------------------------------------------------------------------
AGAP008601  ----------------------------------------------------------------------
CPIJ007987  ----------------------------------------------------------------------
AAEL010317  ----------------------------------------------------------------------
AGAP011084  ----------------------------------------------------------------------
AAEL013950  ----------------------------------------------------------------------
CPIJ008842  ----------------------------------------------------------------------
AGAP009808  ----------------------------------------------------------------------
AAEL008500  ----------------------------------------------------------------------
AGAP009135  ----------------------------------------------------------------------
CPIJ000753  ----------------------------------------------------------------------
AAEL013359  ----------------------------------------------------------------------
AGAP009863  ----------------------------------------------------------------------
AAEL014414  ----------------------------------------------------------------------
AGAP003089  ----------------------------------------------------------------------
AAEL009285  E-EAVSVQKHDYPHTHWGRHNAKLDQKKMRDLG--LKSADQIVKERIQKETKQAKEKVARLRNLARKKKT
CPIJ008599  FGARPTVTRMDFPHTHWGRHNAKVAAKKQREGTSELRSVEQIAKTRLDKEEKLAREKVARLKNLARKKRS
AGAP007511  AAPSAPIVQRHYPTTHWGRHNAKADLRKLRDLD--LKTPEQIVKKRMEKETKMAREKAARLKNIQRKKRA
AAEL011744  ----------------------------------------------------------------------
```

```
CPIJ015074  ----------------------------------------------------------------------
AGAP004711  ----------------------------------------------------------------------
CPIJ000951  ----------------------------------------------------------------------
CPIJ006204  ----------------------------------------------------------------------
AAEL001769  SYGSMSTASSYQS--TNQFPSSQSRPYGSRPNVPSAVGSGV-----------------------------
CPIJ016569  SFGTLNGSAGYGAGGAGGFSSSTS----ARPY--------------------------------------
AGAP004912  APYGAGVGYQYAAPPPTAMPRTDG--YPPRGVIPSMATGGM-----------------------------
AAEL002083  -----FGGYQRSTPAVPSAYPSFGGALPQPGQTFAFPPPAVALN--------------------------
CPIJ014935  GAAPSYGAYPRPAPALAS--YQFASMPPQGGQAFAFPPPPVAMN--------------------------
AGAP005652  YSARSQSSALPSAAAAAVAAAAYQYAANGATPAFAYPPPQVVLN--------------------------
AAEL008738  AMGTFTSSYQIPAAAA-YYSYPAAAAPPPPPQTQAPPVVPPVQQ--------------------------
CPIJ012512  AMGTFASSYQIPAAAA-YYSYPAAAAPPPPPQTQTPQVVPPVQQ--------------------------
AGAP012045  ----YQASYPITAATATYYPYPAATAPPPPPQAQAAPVVPPVQQ--------------------------
AAEL002351  --------------RSRSREGRFRSARNGGTNGTSYDRH-------------------------------
CPIJ003935  ----------------------------------------------------------------------
AGAP005351  DRGRESNRDRDRRRRSRSRSRSRSRSRNGNGRASSYDRY-------------------------------
AAEL010787  SNGYSGSSNGYGNSSSSYRNGTSGGSSNTKHIKFDD----------------------------------
CPIJ009445  ----------------------------TPVIAVG-----------------------------------
AGAP003663  ----------------------------------------------------------------------
AAEL004978  ----------------------------------------------------------------------
CPIJ009286  ----------------------------------------------------------------------
AGAP008578  ----------------------------------------------------------------------
CPIJ014361  ----------------------------------------------------------------------
AGAP003047  ----------------------------------------------------------------------
AAEL010402  ----------------------------------------------------------------------
AAEL001317  ----------------------------------------------------------------------
AGAP012523  ----------------------------------------------------------------------
CPIJ019196  ----------------------------------------------------------------------
AAEL013985  AQISEYSEAGLTVRGTYVPTGKTPPEGERKLYLAIESCSELAVTKAKREITRLIKEELLKLQASSHHIIN
CPIJ005545  AQISEYSEAGLTVRGTYVPPGKNPPEGERKLYLAIESCSELAVTKAKREITRLIKEELLKLQAS-HHIVN
AGAP010656  AQISEYSEAGLTVRGTYVPPGKNPPDGERKLYLAIESCNELAVTKAKREITRLIKEELLKLQASSHHVIN
CPIJ014038  ----------------------------------------------------------------------
dmeRm62     ----------------------------------------------------------------------

CPIJ010448  -----------
AGAP008601  -----------
CPIJ007987  -----------
AAEL010317  -----------
AGAP011084  -----------
AAEL013950  -----------
CPIJ008842  -----------
```

```
AGAP009808  -----------
AAEL008500  -----------
AGAP009135  -----------
CPIJ000753  -----------
AAEL013359  -----------
AGAP009863  -----------
AAEL014414  -----------
AGAP003089  -----------
AAEL009285  LGKKMAKSKGK
CPIJ008599  INKKMKG-KAK
AGAP007511  LAKRQKKGK--
AAEL011744  -----------
CPIJ015074  -----------
AGAP004711  -----------
CPIJ000951  -----------
CPIJ006204  -----------
AAEL001769  -----------
CPIJ016569  -----------
AGAP004912  -----------
AAEL002083  -----------
CPIJ014935  -----------
AGAP005652  -----------
AAEL008738  -----------
CPIJ012512  -----------
AGAP012045  -----------
AAEL002351  -----------
CPIJ003935  -----------
AGAP005351  -----------
AAEL010787  -----------
CPIJ009445  -----------
AGAP003663  -----------
AAEL004978  -----------
CPIJ009286  -----------
AGAP008578  -----------
CPIJ014361  -----------
AGAP003047  -----------
AAEL010402  -----------
AAEL001317  -----------
AGAP012523  -----------
CPIJ019196  -----------
AAEL013985  KSRYKVL----
```

```
CPIJ005545  KSRYKVI----
AGAP010656  KARYKVV----
CPIJ014038  -----------
dmeRm62     -----------
```

Additional File 1G.

Gene Name	Super Contig	Exon 1	Exon 2	Exon 3	Exon 4	Exon 5	Exon 6	Exon 7	Exon 8	Exon 9	Exon 10	Exon 11
cpiAGO1	3.109	93014-93157	91873-92490	91724-91819	91441-91664	91134-91379	90905-91071	86737-87076	86505-86675	85929-86450	81397-81569	81183-81323
cpiAGO2-1A	3.588	269256-269417	269139-269196	268644-269025	266797-267884	265891-266666						
cpiAGO2-1B	3.588	269256-269417	269138-269196	268644-269079	267941-268029	266710-267886	265891-266656	265739-265833				
cpiAGO2-2	3.25	3957-4121	4176-4388	4452-5001	24595-25738	25798-26560						
cpiAGO3	3.94	527023-529364	523095-523245	522692-523031								
cpiPIWI1	3.32	1094385-1094516	1090324-1092838									
cpiPIWI2	3.32	1107424-1107510	1106246-1107360	1100557-1101498	1100222-1100488							
cpiPIWI3A	3.32	1094414-1094516	1090324-1092815									
cpiPIWI3B	3.32	1094389-1094516	1090324-1092838									
cpiPIWI4A	3.379	409199-410432	408939-409152	407954-408889	407633-407898							
cpiPIWI4B	3.379	409199-410432	408939-409152	408768-408889	407954-408742	407633-407898						
cpiPIWI5A	3.3997	123988-125150	123722-123928	122228-123164	121902-122168							
cpiPIWI5B	3.3997	123988-125150	123722-123928	123047-123164	122618-122915	122228-122590	121902-122168					
agaAGO2	Chrom. 3L	26931045-26931563	26916538-26916649	26912993-26914153	26912153-26912906	26911901-26912065						

Alternate Gene structures submitted to Vector Base. Exon values indicate location on genome.

Additional File 1. Mosquito orthologs compared to D. melanogaster and ClustalW alignments using Gonnet weight matrix. Dots indicate amino acid identity to that of the reference sequence. 1A. Mosquito and drosophilid SRRP ortholog accession numbers. 1B. Ago1 orthologs. 1C. Ago2 orthologs. 1D. Ago3 orthologs. 1E. PIWI orthologs. 1F. Rm62-like proteins. 1G. Alternate Gene structures.

Additional File 2. Argonaute Protein Family synapomorphies.

Gene family	Synapomorphy	Explanation
Ago1	VFDEPVI	*1st catalytic residue, exception- Drosophila
Ago2	GAD	1st catalytic residue (underlined)
Ago3	GID	1st catalytic residue (underlined)
All PIWIs	GFD	1st catalytic residue (underlined), distinguishes PIWI-like proteins from Ago1, Ago2, and Ago3, but does not differentiate Ago4 group from Ago5 group
Ago4-like	ETGIQVLNLILRRAMNGLNL<br>QLVGRNLY	agaAgo4, aa 229-255, no mismatches<br>aaePIWI4, aa 200-226, 2 mismatches<br>aaePIWI1, aa 14-31, 1 mismatch<br>aaePIWI2, aa 215-242, 2 mismatches<br>aaePIWI3, aa 222-249, 2 mismatches<br>cpiPIWI3A, aa 205-232, 2 mismatches<br>cpiPIWI3B, aa 223-250, 2 mismatches<br>cpiPIWI1, aa 221-248, 2 mismatches<br>cpiPIWI2, aa 210-237, 2 mismatches<br>dmPIWI, aa 191-218, 8 mismatches<br>dmAub, no matches
Ago5-like	Variable	

* See Figure 2 and Additional File 1 for sequence information.
