# Supplementary material for: Comparative genomics of small RNA regulatory pathway components in vector mosquitoes
Source: BMC Genomics. 2008 Sep 18;9:425. doi: 10.1186/1471-2164-9-425 (PMC2566310; doi:10.1186/1471-2164-9-425)
Supplement: Additional File 2 — Argonaute Protein Family synapomorphies. Signature protein features of protein classes. [file 1471-2164-9-425-S2.pdf]

Additional File 2. Argonaute Protein Family synapomorphies.

Gene family	Synapomorphy	Explanation
Ago1	VFDEPVI	*1st catalytic residue, exception- Drosophila
Ago2	GAD	1st catalytic residue (underlined)
Ago3	GID	1st catalytic residue (underlined)
All PIWIs	GFD	1st catalytic residue (underlined), distinguishes PIWI-like proteins from Ago1, Ago2, and Ago3, but does not differentiate Ago4 group from Ago5 group
Ago4-like	ETGIQVLNLILRRAMNGLNL QLVGRNLY	agaAgo4, aa 229-255, no mismatches<br>aaePIWI3, aa 200-226, 2 mismatches<br>aaePIWI1-1, aa 14-31, 1 mismatch<br>aaePIWI1-2, aa 215-242, 2 mismatches<br>aaePIWI2, aa 222-249, 2 mismatches<br>cpiPIWI1-3A, aa 205-232, 2 mismatches<br>cpiPIWI1-3B, aa 223-250, 2 mismatches<br>cpiPIWI1-1, aa 221-248, 2 mismatches<br>cpiPIWI1-2, aa 210-237, 2 mismatches<br>dmPIWI, aa 191-218, 8 mismatches<br>dmAub, no matches
Ago5-like	Variable	

* See Figure 2 and Additional File 1 for sequence information.
