# Supplementary figures and images for: Comparative genomics of small RNA regulatory pathway components in vector mosquitoes
Source: BMC Genomics. 2008 Sep 18;9:425. doi: 10.1186/1471-2164-9-425 (PMC2566310; doi:10.1186/1471-2164-9-425)

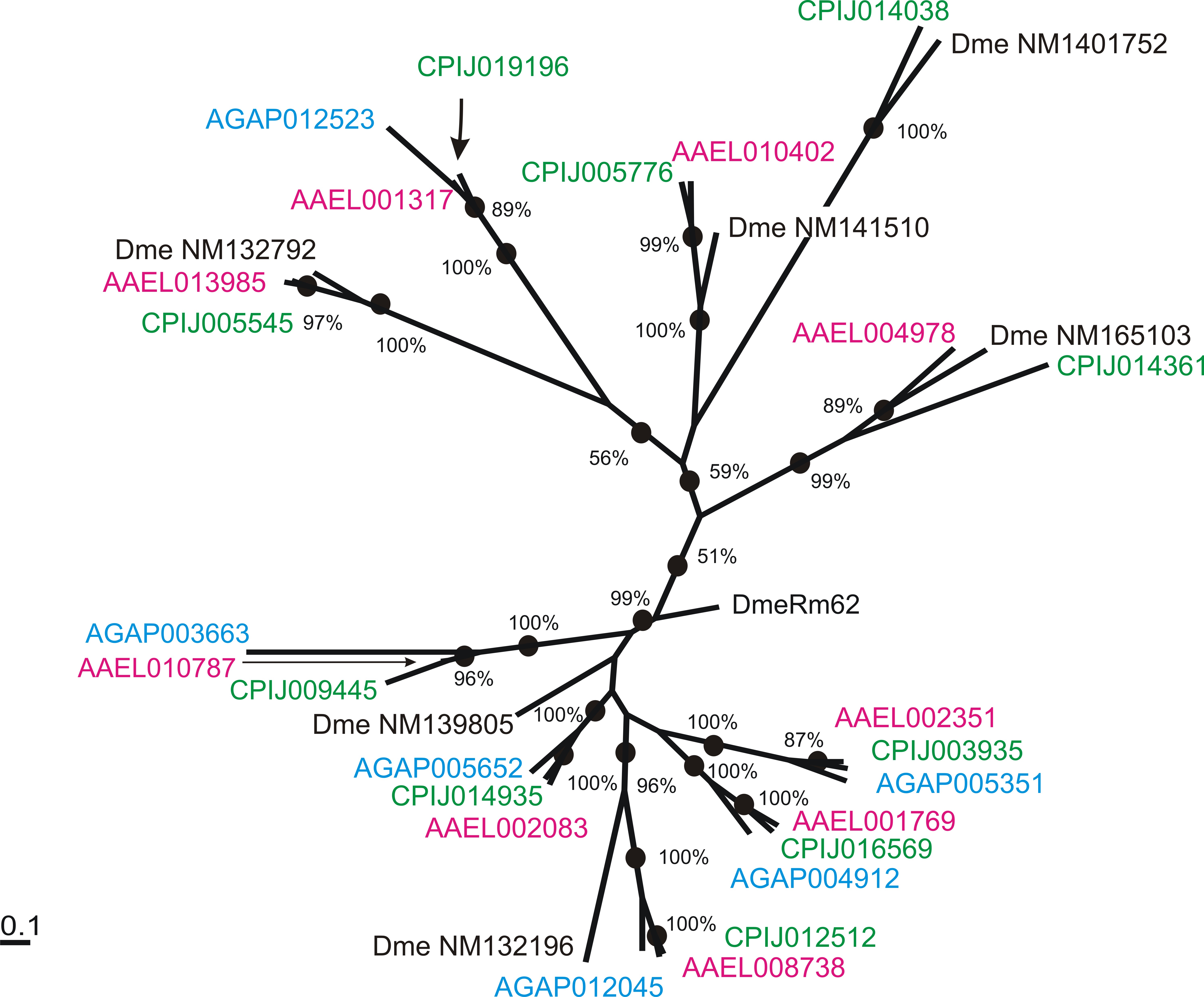

Supplement: Additional File 3 — Rm62-like Protein Tree. Maximum Likelihood tree of Mosquito Rm62-like proteins compared to Drosophila Rm62-like helicases. [file 1471-2164-9-425-S3.jpeg]
